# Natural Products from Actinobacteria Associated with Fungus-Growing Termites

**DOI:** 10.3390/antibiotics7030083

**Published:** 2018-09-13

**Authors:** René Benndorf, Huijuan Guo, Elisabeth Sommerwerk, Christiane Weigel, Maria Garcia-Altares, Karin Martin, Haofu Hu, Michelle Küfner, Z. Wilhelm de Beer, Michael Poulsen, Christine Beemelmanns

**Affiliations:** 1 Leibniz Institute for Natural Product Research and Infection Biology—Hans-Knöll-Institute, Beutenbergstraße 11a, 07745 Jena, Germany; rene.benndorf@hki-jena.de (R.B.); huijuan.guo@hki-jena.de (H.G.); e.sommerwerk@gmail.com (E.S.); christiane.weigel@leibniz-hki.de (C.W.); maria.garcia-altares@leibniz-hki.de (M.G.-A.); karin.martin@leibniz-hki.de (K.M.); michelle.kuefner@gmx.de (M.K.); 2 Section for Ecology and Evolution, Department of Biology, University of Copenhagen, 2100 Copenhagen East, Denmark; Haofu.Hu@bio.ku.dk (H.H.); MPoulsen@bio.ku.dk (M.P.); 3 Department of Microbiology and Plant Pathology, Forestry and Agriculture Biotechnology Institute, University of Pretoria, Pretoria 0001, South Africa; wilhelm.debeer@fabi.up.ac.za

**Keywords:** actinobacteria, symbiosis, secondary metabolites, drug discovery, chemical ecology

## Abstract

The chemical analysis of insect-associated Actinobacteria has attracted the interest of natural product chemists in the past years as bacterial-produced metabolites are sought to be crucial for sustaining and protecting the insect host. The objective of our study was to evaluate the phylogeny and bioprospecting of Actinobacteria associated with fungus-growing termites. We characterized 97 Actinobacteria from the gut, exoskeleton, and fungus garden (comb) of the fungus-growing termite *Macrotermes natalensis* and used two different bioassays to assess their general antimicrobial activity. We selected two strains for chemical analysis and investigated the culture broth of the axenic strains and fungus-actinobacterium co-cultures. From these studies, we identified the previously-reported PKS-derived barceloneic acid A and the PKS-derived rubterolones. Analysis of culture broth yielded a new dichlorinated diketopiperazine derivative and two new tetracyclic lanthipeptides, named rubrominins A and B. The discussed natural products highlight that insect-associated Actinobacteria are highly prolific natural product producers yielding important chemical scaffolds urgently needed for future drug development programs.

## 1. Introduction

Historically, natural products of microbial origin have been a rich source of drug-like lead structures and until today, almost 35% of all drugs are based on structures of naturally occurring small molecules [1,2,3]. Despite this prevalence, natural product chemistry has faced declining enthusiasm and dwindling investments for decades as bioactivity-guided screening programs resulted mostly in the rediscovery of already known compounds. The low success rates of industrial antibiotic drug discovery programs worldwide resulted in todays’ eminent lack of new antibiotic drug leads. At the same time, increasing numbers of multiresistant human-pathogenic microbes causing non-treatable infections in clinics are reported. These eminent health threats led to the recent realization that new natural product derived scaffolds are urgently needed to combat the life threating infections caused by multidrug resistant pathogens.

The revolutionary developments in genome sequencing and analytical technologies in the last decade have dramatically changed the field of natural product discovery [4]. Particularly, ecology-driven natural product discovery approaches including the chemical analyses of symbiotic microorganisms, in combination with omics-based dereplication strategies, have become highly efficient approaches to identify new natural products with unique chemical scaffolds and bioactivities [5,6,7,8]. Most notably, the analysis of insect-microbe symbioses, and more specifically insect-Actinobacteria interactions, have been the focus of a series of recent natural product discovery studies, as bacterial symbionts are required to communicate with the host or participate in host defense using small molecules. The importance of defensive secondary metabolites in insect-Actinobacteria symbiosis is evident, as exemplified in firebugs (Pyrrhocoridae: *Pyrrhocoris apterus*) [9] or the European beewolf (Crabronidae: *Philanthus*) that harbors antibiotic-producing *Streptomyces* in their antennae to help protect wasp larvae from fungal infections [10]. Similarly, fungus-growing ants (*Attini* species) carry symbiotic *Pseudonocardia* that help protect the ants’ fungal gardens against specialized parasites [11,12,13]. Insect-associated Actinobacteria have also been reported from other insects, including Ambrosia beetles [14], dung beetles (Scarabaeidae: *Copris tripartitus*) [15,16], and fungus-growing termites [17,18].

We have recently focused efforts on the chemical analyses of the delicate interplay between fungus-growing termites (Termitidae: Macrotermitinae), their fungal mutualist *Termitomyces* (Basidiomycota: Agaricales: Lyophyllaceae), and bacteria residing within termite guts and fungus gardens (fungus combs). Fungus-growing termites cultivate the fungal mutualist in subterranean monoculture fungus gardens as their main food source [19]. The maintenance of such a monoculture in a nutritionally-rich environment is expected to make the fungal garden prone to exploitation by competitors and disease, such as mites, nematodes, and co-occurring fungi. In addition to antimicrobial and behavioral defense mechanisms of the termites themselves [20], it has been hypothesized that bacteria are employed as defensive symbionts [17,21].

**Figure 1:**
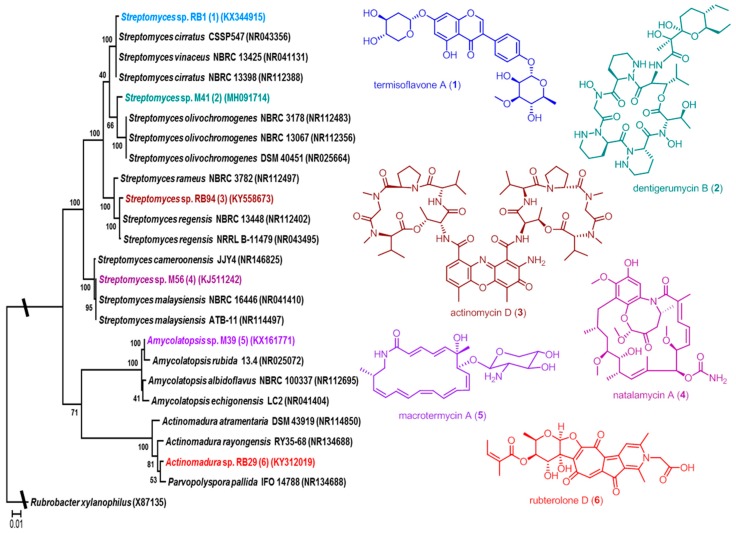
Phylogenetic placement Actinobacteria that have previously been reported from fungus-growing termites and isolated natural products: termisoflavone A (**1**) from *Streptomyces* sp. RB1, dentigerumycin B (**2**) from *Streptomyces* sp. M41, actinomycin D (**3**) from *Streptomyces* sp. RB94, natalamycin A (**4**) from *Streptomyces* sp. M56, macrotermycin A (**5**) from *Amycolatopsis* sp. M39, and rubterolone D (**6**) from *Actinomadura* sp. RB29.

Using bacteria-fungus interaction assays, we have demonstrated that Actinobacteria associated with *Macrotermes natalensis* secrete secondary metabolite mixtures that are active against co-occurring fungi and the weed fungus *Pseudoxylaria* sp. (Ascomycota: Xylariales: Xylariaceae) [22]. Subsequent analysis of single species resulted in the isolation of a new geldanamycin derivative, named natalamycin (**4**), from *Streptomyces* sp. M56 (Figure 1) [23]. In a follow-up study, activity-based analysis of *Amycolatopsis* sp. M39 identified several new macrolactams named macrotermycins (macrotermycin A, **5**) [24], and comparative genome and metabolomic analysis of *Streptomyces* sp. M41 yielded, amongst others, the novel depsipeptide dentigerumycin B (**2**) [25]. Recently, activity and NMR-guided analysis of *Streptomyces* sp. RB1 led to the isolation of termisoflavones A–C (termisoflavone A, **1**) [26], and co-cultivation studies of *Actinomadura* sp. RB29 yielded a new group of tropolone derivatives named rubterolones (e.g., rubterolone D, **6**) [27].

Here, we present a comprehensive phylogenetic and bioactivity survey of Actinobacteria associated with the fungus-growing termite *M. natalensis*. In summary, our study shows that bioprospecting for secondary metabolites in Actinobacteria associated with this termite species leads to the identification of novel natural with unique chemical scaffolds and provides a foundation for gaining a better understanding of the general sanitary role of Actinobacteria in fungus-growing termites.

## 2. Results

### 2.1. Phylogenetic Diversity

**Figure 2:**
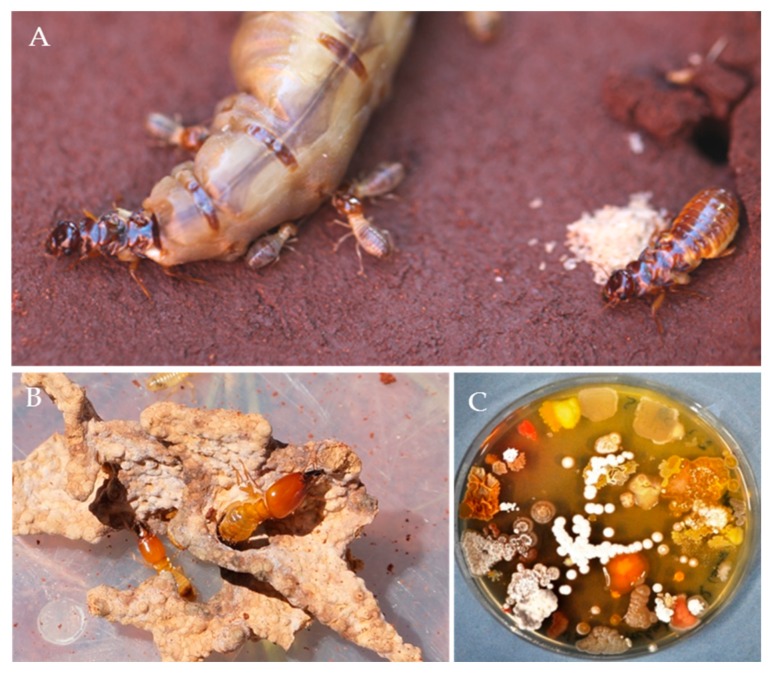
(**A**) Royal chamber of *Macrotermes natalensis* containing the queen, the king, and workers. (**B**) Fungus comb with a major and a minor soldier. (**C**) A plate exemplifying the diversity of culturable bacteria that can be isolated from the gut of a fungus-growing termite worker.

**Figure 3:**
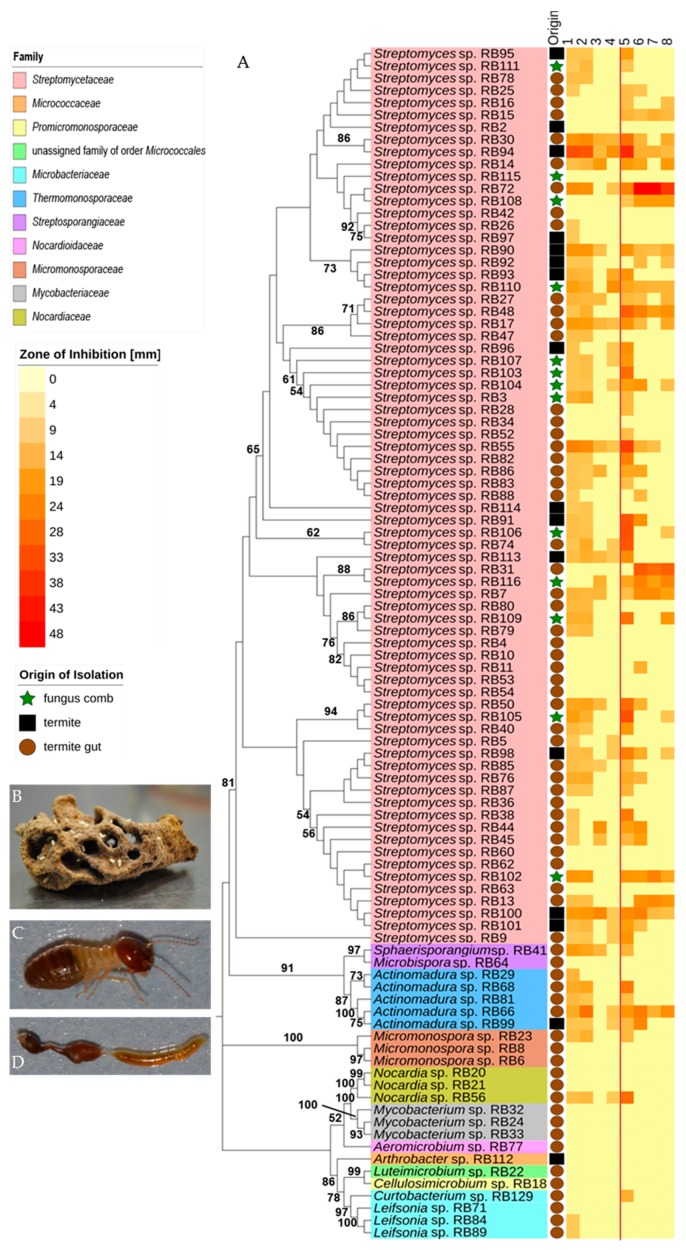
Phylogeny and antimicrobial activity of newly isolated Actinobacteria: (**A**) Phylogenetic analysis based on near full-length 16S rRNA sequences of isolated Actinobacteria including phylogenetic placement to the family level. An unrooted neighbor-joining distance tree is shown with branch values indicating bootstrap support (>50 are given) of 1000 pseudoreplicates, tree was constructed with Mega 7.0 and edited with iTOL v3. Middle: Origin of isolation: termite abdomen: black box, termite gut: brown circle, fungus comb: green star. Right: activity heatmap against test strains *Bacillus subtilis* ATCC 6633 (1), *Staphylococcus aureus* IMET 10760 (2), *Escherichia coli* SG 458 (3), *Pseudomonas aeruginosa* K799/61 (4), *Mycobacterium vaccae* IMET 10670 (5), *Sporobolomyces salmonicolor* SBUG 549 (6), *Candida albicans* BMSY 212 (7) and *Penicillium notatum* JP36 (8). Representative picture of (**B**) fungus comb, (**C**) major worker, (**D**) dissected gut of major worker.

**Figure 4:**
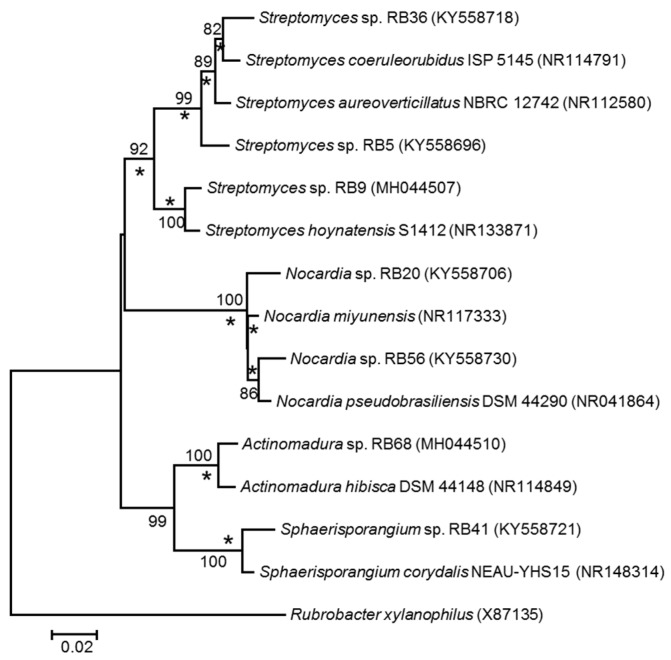
Rooted neighbor-joining tree based on near-complete 16S rRNA gene sequences showing relationship between putative new Actinobacteria strains (based on 98.65% similarity threshold) and closest relatives. Stars (*) indicate branches that were also recovered in maximum-likelihood tree. Only bootstrap values above 50% (based on 1000 pseudoreplicates) are shown. *Rubrobacter xylanophilus* was used as an outgroup. The scale bar indicates 0.02 substitutions per nucleotide position.

To assess the culturable actinobacterial diversity, we chose three different sample origins (fresh fungus comb material and the termite worker exoskeleton and gut content) from eleven different *M. natalensis* termite colonies collected in South Africa (Table S1). We focused on the isolation of Actinobacteria capable of living on cellulose or chitin as a sole C-source (Figure 2), as these bacterial isolates are likely adapted to living within the cellulose-rich comb material [28]. Actinobacteria (97) with unique morphotypes were isolated from the termite gut (68), termite abdomen (13), and fungus comb material (16) (Table 1, Table S2). Subsequent phylogenetic analysis of all isolates using 16S rRNA sequencing was conducted, showing that the characterized isolates did not form a monophyletic group (Figure 3, Figure S1) but were interspersed within the Actinobacterium phylum [17,29]. Interestingly, 73 isolates belonged to the genus *Streptomyces* and covered most of the reported phylogenetic diversity of this widespread genus. The remaining 24 isolates belonged to 12 genera within the Actinobacteria (Table 1). For species delineation a threshold of <98.65% sequence similarity was applied revealing seven putative new Actinobacteria species (Figure 4, Table S3) [30,31].

### 2.2. Antimicrobial Activities Against Test Strains

**Figure 5:**
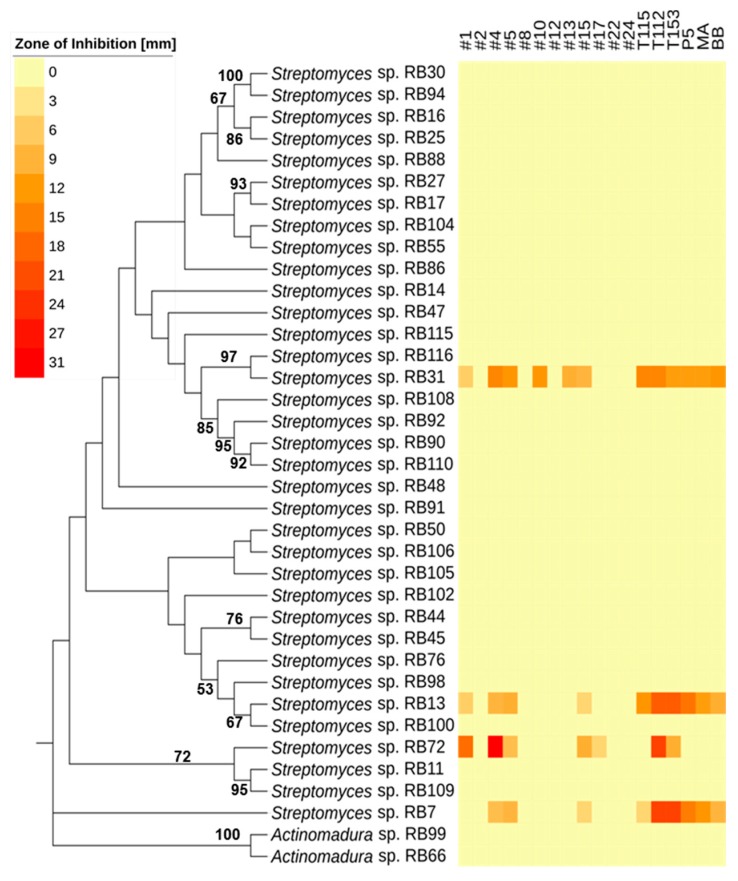
Phylogeny and antifungal activity of isolated Actinobacteria. Left: Phylogenetic analysis based on near full-length 16S rRNA sequences of isolated Actinobacteria. An unrooted maximum-likelihood distance tree is shown with branch values indicating bootstrap support (>50 are given) of 1000 pseudoreplicates; tree was constructed with Mega 7.0 and edited with iTOL v3. Right: Antifungal activity assays of 37 different culture extracts against ecologically relevant fungi: #1: *Cladosporium* sp., #2: *Cladosporium* sp., #4: *Pleosporales* sp., #5: *Fusarium* sp., #8: *Coriolopsis* sp., #10: *Fusarium* sp., #12: *Cunninghamella* sp., #13: *Cladosporium* sp., #15: *Alternaria* sp., #17: *Trichoderma* sp., #22: *Trichoderma* sp., #24: *Hypocrea* sp., T115: *Termitomyces* sp., T112: *Termitomyces* sp., T153: *Termitomyces* sp., P5: *Termitomyces* sp., MA: *Metarhizium anisopliae* ATCC 24942, and BB: *Beauveria bassiana* ST 17960.

We first assessed the bioactivities of standardized culture extracts (1 mg/mL) of all 97 isolates against a panel of test strains, including human-pathogens (the Gram-positive bacterium *Staphylococcus aureus*, the Gram-negative bacterium *Pseudomonas aeruginosa*, and the fungus *Candida albicans*) (Figure 3, Table S5). While most *Streptomyces* strains produced compounds with antibacterial and antifungal properties, we observed varying intensities of activity. On average, *Streptomyces* extracts (73) inhibited four test strains, but individual strains varied substantially in the number of test strains they suppressed. Several *Streptomyces* extracts showed only antibacterial activity (e.g., *Streptomyces* sp. RB74, *Streptomyces* sp. RB106 and *Streptomyces* sp. RB113), while others exhibited only antifungal activity (e.g., *Streptomyces* sp. RB31). Most notably, *Streptomyces* sp. RB94 and RB100 showed strong antibacterial activity. In contrast, 11 out of 73 *Streptomyces* strains (15%) inhibited none of the tested strains. Isolates belonging to the genera *Actinomadura*, *Sphaerisporangium* and *Micromonospora* inhibited on average three bacterial test strains and only two strains showed antifungal activity (*Actinomadura* sp. RB66 and *Actinomadura* sp. RB99). In contrast, extracts obtained from isolates belonging to *Arthrobacter*, *Cellulosimicrobium*, *Aeromicrobium, Luteimicrobium* and *Mycobacterium* showed almost no inhibitory activity against any of the investigated test strains. Thirty-seven extracts with moderate to strong antifungal activity were subjected to a second antifungal assay against ecologically-relevant co-occurring fungi derived from termite nests, four different fungal cultivar isolates (two species) and two entomopathogenic fungi (*Beauvaria bassiana* ST 17960 and *Metarhizium anisopliae* ATCC 24942) [32,33]. As depicted in Figure 5, the majority of culture extracts inhibited none of the representative competing or mutualistic fungi. Extracts of four strains (*Streptomyces* sp. RB7, RB72, RB13, and RB31) did, however, inhibit on average ten of the ecologically-relevant fungal test strains, including the entomopathogenic fungi and *Termitomyces* (Table S6). Interestingly *Streptomyces* sp. RB116 and *Streptomyces* sp. RB31 have the same closest type strains (Table S3) in the blast search in NCBI (https://blast.ncbi.nlm.nih.gov/Blast.cgi, last visit 26th of July, 2018, 00:58 AM) [34], but show variation in antifungal activity (Figure 5, Table S8). 

### 2.3. Chemical Analysis

As gene expression of important biosynthetic clusters often is under the control of promoters that respond to certain external factors, cultivation using standard laboratory conditions is likely to lead to limited amount of secondary metabolite production. To activate so-called “cryptic” gene clusters, it is often necessary to mimic natural key stress factors such as limited nutrient availability or the presence of other potentially competing organisms, as exemplified in a recent study of *Amycolatopsis* sp. M39 [24]. We therefore selected *Actinomadura* sp. RB29 and *Streptomyces* sp. RB108, and subjected both strains to co-cultivation set-ups against co-isolated fungi to stimulate the production of cryptic metabolites. 

**Figure 6:**
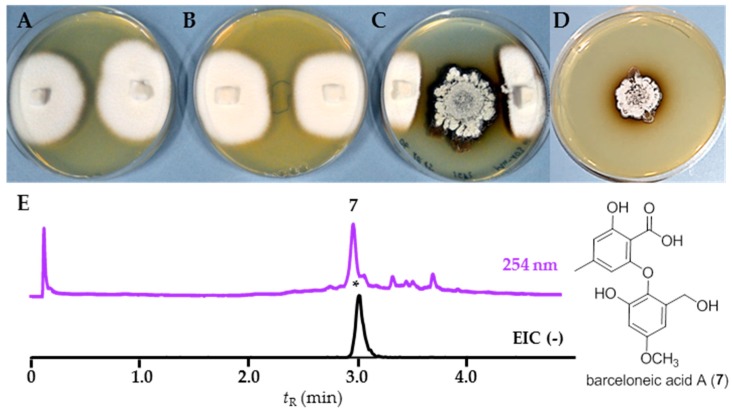
Co-cultivation studies of strain RB108 with *Pleosporales* sp. #4: (**A**) negative control: axenic *Pleosporales* sp. #4; (**B**) positive control: *Pleosporales* sp. #4 in the presence of amphotericin B (8 mg/mL, middle); (**C**) interaction assay of *Streptomyces* sp. RB108 (middle) with *Pleosporales* sp. #4 (edge of the plate); (**D**) axenic *Streptomyces* sp. RB108; (**E**) structure of barceloneic acid A (**7**); and representative UHPLC-MS analysis (254 nm) of zone of inhibition extracts: EIC (–) of barceloneic acid A (**7**) at *m/z* 319.0.

**Figure 7:**
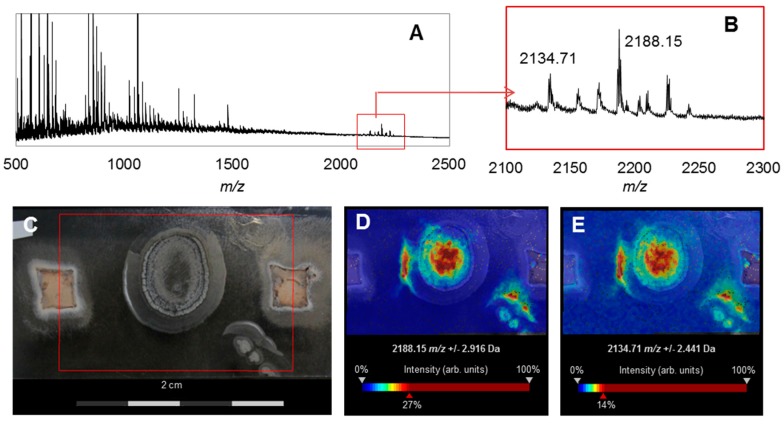
MALDI imaging of a co-cultivation study of *Streptomyces* sp. RB108 with *Pleosporales* sp. #4: (**A**) average MS spectra of the MALDI Imaging MS analysis (TIC normalization) and red rectangle defines the region of the spectra zoomed in; (**B**) extended view of the region from 2100 to 2300 *m*/*z* showing the upregulated RiPPs; (**C**) photograph of co-culture set up: RB108 grown in the middle and right lower side; *Pleosporales* sp. #4 grown on agar plugs on the right and left edge of the plate. Area covered by the MALDI Imaging MS analysis defined in red; (**D**) visualization of the most intense peak ion *m*/*z* 2188.15 (TIC normalization, weak denoising); and (**E**) visualization of the second most intense peak ion *m*/*z* 2134.71 (TIC normalization, weak denoising).

First, we tested strain RB108, as comparative phenotypical and phylogenetic analyses indicated it to be a novel *Streptomyces* species (Figure S1, Table S3). Although standardized extracts of RB108 showed only moderate antifungal activity (Figure 3, Table S5), co-cultivation induced a strong antifungal activity against almost all tested fungal strains (Table S9) and an increased brownish pigment production. Due to the large inhibition zone, we selected co-cultivation set-up RB108/*Pleosporales* sp. #4 for in-depth analysis. We performed comparative ultra-performance-liquid chromatography-mass spectrometry (UHPLC-MS, Shimadzu, Japan) analysis of concentrated extracts obtained from the zone of inhibition (ZOI). In addition to several other upregulated signals of minor intensity, a distinct UV-detectable metabolite (*m*/*z* = 303.1/321.1) was found to be only produced in co-cultivation with *Pleosporales* sp. #4. Mass spectrometry (MS)-guided high pressure liquid chromatography (HPLC, Shimadzu, Japan) purification resulted in the isolation of barceloneic acid A (**7**) (Figure 6), a fungal metabolite acting as a farnesyl-protein transferase inhibitor [35]. We then performed Matrix Assisted Laser Desorption Ionization Imaging MS (MALDI Imaging MS, Bruker Daltonics) to resolve the spatial distribution of **7** and to identify possible antifungal candidates from RB108. However, due to low ionization capacity of this compound class, a clear spatial location of barceloneic acid A (**7**) was not observable. Instead, the detailed analysis of the co-culture assay revealed a cluster of ions with *m/z* values between 2000 Da and 2500 Da (*m/z* 2188.15 and *m/z* 2134.71 being the most intense) that were upregulated and accumulated in the center and on the edges of the colonies facing the fungus *Pleosporales* sp. #4 (Figure 7). This *m/z* range is typical for ribosomally-synthesized peptides (RiPPs) often associated with high antimicrobial activities. We currently hypothesize that *Pleosporales* sp. #4 modulates the interaction with strain RB108 using barceloneic acid A (**7**) and stimulates the production of RiPPs of yet unknown composition. 

**Figure 8:**
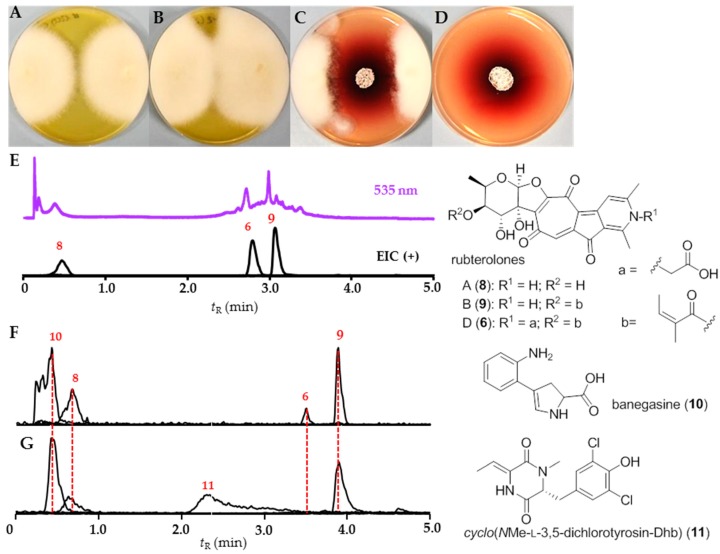
Co-cultivation studies of strain RB29 with *Trichoderma* sp. #22: (**A**) negative control: axenic *Trichoderma* sp. #22; (**B**) positive control: *Trichoderma* sp. #22 in the presence of AmpB; (**C**) co-cultivation of *Actinomadura* sp. RB29 (middle) and *Trichoderma* sp. #22 (edge of the plate); (**D**) axenic culture of *Actinomadura* sp. RB29; (**E**) Representative UHPLC-MS analysis (535 nm) of zone of inhibition extracts: EIC (+) of rubterolone A, B, and D at *m*/*z* 414.1, 496.1, and 554.1 and structures of isolated rubterolone A (**8**), B (**9**), and D (**6**); (**F**) production of banegasine (**10**) on ISP2 agar plate and extract with 1% AcOH containing MeOH; and (**G**) production of cyclo(*N*Me-L-3,5-dichlorotyrosine-Dhb) (**11**) in Soya Broth (here represent from XAD16 80% MeOH eluate).

In a second study, we pursued a comparative UHPLC-MS analysis of *Actinomadura* sp. RB29 as co-cultivation against, for example, *Trichoderma* sp. #22, which induced strong antifungal activities (Figure 8). In contrast, no antifungal properties were originally observed in standard culture extracts (Figure 3).

By tracing back the antifungal agents, comparative analysis of culture extracts resulted in the detection of predominant rubterolone derivatives (**6**, **8**, and **9**) [27] and a unique *m/z* signal pattern indicative for a dichlorinated natural product (**11**). Subsequent purification of co-cultivation and liquid culture extracts by semipreparative HPLC led to the isolation of the natural product banegasine (**10**) and the chlorinated natural product cyclo(*N*Me-L-3,5-dichlorotyrosine-Dhb) (**11**) (Table S7) [36].

We also analyzed standard culture extracts using LC-HRMS and detected, in addition to several of the previously reported rubterolone derivatives, a RiPP-type MS^2^ pattern of two parent ions *m*/*z* at 957.8 ([M + 2H]^2+^) and 993.5 ([M + 2H]^2+^) within the SPE 70% and 80% MeOH C18-SPE eluates. Purification by Sephadex LH20 resin and semipreparative HPLC purification resulted in the identification of two tetracyclic lanthipeptides rubrominin A (**12**) and B (**13**). 

The molecular formulas of **12** and **13** were established to be C_80_H_115_N_21_O_28_S_3_ and C_83_H_120_N_22_O_29_S_3_ based on the exact mass analysis of protonated ion **12** (*m/z* 1914.73938 [M + H]^+^, calcd. 1914.7 Δ = −3.21 ppm) and **13** (*m/z* 1985.77539 [M + H]^+^, calcd. 1985.77141 Δ = 2.01 ppm). The mass difference of 71.03601 between **1**2 and **13** suggested an additional alanine residue from the *N*-terminus. The MS^2^ spectra were recorded, submitted to Global Natural Product Social Molecular Networking (GNPS) and processed by RiPPquest [37]. The combined analysis led to the identification of a putative candidate peptide ([A]CSSTCTSGPFTFACDGTTKG), which is presumably modified by dehydrations and oxidation reactions. However, the estimate *p*-value of PSM (peptide-spectrum matches) did not allow an assignment of the modified positions. 

**Figure 9:**
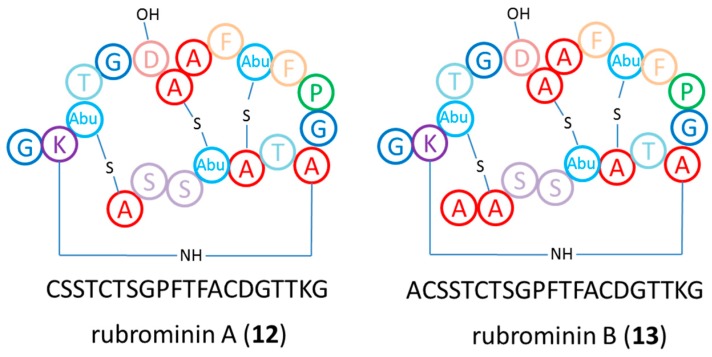
Proposed structures of lanthipeptides rubrominin A (**12**) and B (**13**).

Genome analysis of *Actinomadura* sp. RB29 using antiSMASH [38] and Blast resulted in the identification of a cinnamycin-homolog gene cluster [39,40], which we named *rum*. It contains 21 open reading frames (ORF) and homolog genes to *cinA*, *cinM*, *cinX*, and *cinorf7* from *Streptomyces cinnamoneous* (Figure S6, Table S11). The candidate peptide sequence ([A]CSSTCTSGPFTFACDGTTKG) is located at the *N*-terminus of RumA. Furthermore, a precursor peptide sequence and an AYA (AXA motif) between the *C*-terminus leader sequence and the core region of RumA was identified; both of which are likely be recognized by a type I signal peptidases of the general secretory (sec) pathway. The RumM sequence shows high homology with other class II LanM (lanthipeptide synthetase) enzymes and is likely to catalyze the dehydration of Thr4, Ser7, Thr11, and Thr18 in the precursor peptide and the subsequent addition/cyclization reaction of three Cys residues to form the three methyllanthionine bridges. In addition, RumX, a homolog Nif11 family protein, might hydroxylate Asp15 of the precursor peptide. The Cinorf7 homolog named RumN is presumably involved in the formation of the cross-link between Lys19 and dehydroalanine7 to form a lysinoalanine bridge. We then performed isotope labeling experiments using L-serine-2,3,3-D_3_ and DL-cysteine-3,3-D_2_, which showed the incorporation of both, serine and cysteine, into the core peptide. Subsequent Marfey’s derivatization of both compounds revealed the partial amino acid composition in the matured peptide as L-Phe, L-Ala, Gly, L-Ser, L-Thr, and L-Pro. ^1^H NMR spectra and COSY correlation of **12** and **13** recorded in D_2_O revealed amino acid-like chemical shifts, and 10% D_2_O/90% H_2_O deduced the amide chemical shifts from peptide bonds, in particular phenylalanine and alanine spin systems. Due to the dominant rubterolone formation and low production titers of **12** and **13**, full NMR assignment and evaluation of the bioactivities is the topic of current investigations. Based on the acquired genomic data, MS^2^-analysis and Marfey analysis, we are confident propose the tetracyclic structure of rubrominins A (**12**) and B (**13**) as depicted in Figure 9.

## 3. Discussion

### 3.1. Phylogenetic and Ecological Relevance of the Actinobacteria

Termites forage for a broad range of organic material to manure the mutualistic food fungus and this substrate is expected to contain a broad diversity of fungal and bacterial isolates. After predigestion of the harvested material during a first gut passage [20], the termites deposit the resulting feces as fresh comb material for fungal growth. However, it is a conundrum that active combs lack any signs of the presence of fungal contaminates or diseases. This suggests that the gut and comb environment, including the microbial community, provides effective defenses against any incoming potentially parasitic and competitive species. To test the hypothesis that Actinobacteria provide have the potential to be a line of defense against invading fungal species, and to identify novel antimicrobial metabolites produced by these bacteria, we investigated the culturable actinobacterial diversity of guts, combs, and exoskeletons of the fungus-growing termite species *M. natalensis*. Here, we acknowledge the fact that due to the limited numbers of tested culture conditions, we miss “unculturable” and extremophilic members. 

Overall, Actinobacteria were found throughout all samples and colonies of *M. natalensis* with 97 representatives, covering twelve genera, ten families, and two orders of the known actinobacterial diversity [29]. We found a dominance of diverse *Streptomyces* species (75%) and a low abundances of other genera, such as *Actinomadura*, *Microbispora*, *Micromonospora*, and *Nocardia* consistent with previous findings [17,29,41]. Here, it is interesting to note that *Actinomadura* are frequently isolated from soil [42] and from other social insects such as bee hives [43], similar to members of the genera *Microbispora*, *Micromonospora*, and *Nocardia* [44,45].

Based on the threshold of 98.65% sequence similarity of the 16S rRNA gene for species delimination, we defined seven isolates as putative novel species (Figure 4), and which are currently investigated for their physiological properties and biosynthetic potential.

The dominant isolation rates of *Streptomyces* from all biological samples and the lack of phylogenetic specificity between Actinobacteria genera and the different biological samples (exoskeleton, gut fluids, and fungus comb), may suggest that Actinobacteria are transient microbes. They are presumably taken up as spores or vegetative mycelium present within digested soil particles and then incorporated as part of the fecal deposits within the comb material; a similar strategy allows the introduction and propagation of the fungal mutualist *Termitomyces.*

In particular, the high isolation rate of Actinobacteria from anaerobic gut fluids was intriguing as previous metagenomic studies indicate that the microbial gut community is dominated by members of Firmicutes, Bacteroidetes, Spirochaetes, Proteobacteria, and Synergistetes, with the most abundant genera being anaerobic or microaerophilic, such as *Alistipes*, *Treponema, Desulfovibrio*, *Paludibacter*, and a member of the Synergistaceae [45]. 16S rRNA sequences affiliated with Actinobacteria appear to account for only a minor component of the gut bacterial microbiota. Similar results were obtained in a related study of the fungus-growing termite *Odontotermes formosanus*, with four phylogenetic groups, Firmicutes, the Bacteroidetes/Chlorobi group, Proteobacteria, and Actinobacteria dominating [46]. Considering the anaerobic or microaerophilic environment of the gut compartments, it is likely that only a small fraction of Actinobacteria are actively growing (Table S10) [47,48], and most of our isolates might originate from germination of spores present within the gut fluids [49]. 

In contrast to gut microbial communities, taxonomic analyses of the comb microbiota of different termite genera showed a clear shift in microbial composition to a more dynamic microbiota of about 33 different phyla, with Firmicutes, Bacteroidetes, Proteobacteria, and Actinobacteria (47 families of four different classes) as the most abundant phyla [50]. It has been hypothesized that this shift would allow for a second microbe-assisted aerobic decomposition, detoxification of plant substrates, and defense against invading and potentially non-beneficial microbes. In particular, Actinobacteria have a multitude of enzymatic capabilities to break down polysaccharides (cellulose, chitin, xylan, and agar) [29,41,51], and to detoxify microbial metabolites, metabolic capacities that most likely contribute to the optimal growth conditions for *Termitomyces*.

### 3.2. Bioactivities and Natural Products

Using two standardized bioassays, we explored the antimicrobial activities of associated Actinobacteria. More than three quarters of the actinobacterial isolates produced compounds with antimicrobial activity against one or more test strains (human-pathogenic), but only four of the generated extracts revealed antifungal activity against fungal garden weeds, in addition to inhibitory activity against the fungal cultivar. Here, we acknowledge the possibility that metabolite secretion of isolated bacterial strains is strongly depended on the culture environment. We also noticed that even very closely related strains showed a high variability amongst activities. In subsequent actinobacterium-fungus co-cultivation studies we showed that metabolite production was stimulated in the presence of a fungal species and that strains which previously secreted no antifungal metabolites were stimulated and produced compounds with strong antifungal activity. We decided to analyze two co-culture case studies in detail. In the first case study, we analyzed the interaction zone of a co-culture between *Streptomyces* sp. RB108 and *Pleosporales* sp. #4 using LC-MS and MALDI Imaging to visualize the production of potential “cryptic” metabolites. UHPLC-MS-based analysis of the co-culture between *Streptomyces* sp. RB108 and *Pleosporales* sp. #4 revealed that the fungal metabolite barceloneic acid A (**7**), a known farnesyl-protein transferase inhibitor, was strongly upregulated. Although barceloneic acid A did not reveal any antimicrobial activity, it is likely that the molecule modulates the bacterium-fungal interactions using yet unknown mechanisms. We then used a MALDI imaging approach to identify the origin of the antifungal activities and found an increased production of RiPP-like metabolites, which are known for antimicrobial activities and their detailed structural analysis is topic of recent investigations. Upregulations of RiPP-like metabolites having different *m/z* ranges were also observed in other co-cultivation studies; revealing a glimpse into the plethora of metabolites present within complex multipartner interactions. 

In a second case study, we analyzed the metabolome of *Actinomadura* sp. RB 29 in more detail, as growth studies on different media and co-culture assay induced strong metabolomic shifts and inducible antifungal activity. Comparative analysis revealed that in addition to the previously reported rubterolones, the natural product banegasine (**10**) and dichlorinated diketopiperazine derivative **11** were produced. Banegasine (**10**) displayed moderate antimicrobial activities against Gram-positive bacteria, including *Mycobacteria*, and is known to potentiate the antimicrobial activity of, for example, pyrrolnitrin [52]. Although the dichlorinated diketopiperazine derivative **11** has been previously reported as part of a screening library, its origin has been undisclosed [36]. In general, diketopiperazine derivatives are common secondary metabolites from bacteria and fungi and the combination of natural and modified amino acids produces diverse structural and bioactivity diversity. The structure of compound **11** is unique as it contains two modified amino acids. First, it contains a 2,3-dehydro-2-aminobutyric acid (Dhb), which presumably originates from the dehydration of threonine [53], and is frequently found in bioactive natural products family like nonribosomal peptides [54] and lanthipeptides [55]. Secondly, it contains a 3,5-dichlorotyrosine moiety, which is a building block of several natural products like chloropeptin [56] from *Streptomyces lavendulae*, and cyclo(13,15-dichloro-L-Pro-L-Tyr) from fungi *Leptoxyphium* sp. In addition, the modified amino acid has been detected in cuticles from several insect species, where they might play important roles in the sclerotization process [57]. A similar diketopiperazine *cyclo*(L-*N*-MePhe-Dhb) was identified from *Streptomyces globisporus*, exhibiting interesting morphogenic and biosynthetic regulator effects [58]. Additionally, we also identified two ribosomally synthesized and post-translationally modified peptides, named rubrominins A (**12**) and B (**13**). The post-translational modification reactions include the dehydration of Ser residue to dehydroalanine and a cyclization step that includes the addition of Cys residues to the dehydrated Ser residues yielding the lanthionine and thioether cross-link. The resulting polycyclic peptides, named lanthipeptides, have constrained conformations that often confer their biological activities. Due to the very low production titers of rubrominins A (**12**) and B (**13**) in co-cultures and axenic cultures, their biological activities have not yet been elucidated and is a topic of current investigations. 

Overall, the chemical analysis of *Actinomadura* sp. RB 29 revealed growth-condition dependent metabolite production, and although the origin of the antifungal activity within co-cultures remains to be fully elucidated, the identified natural products (rubterolones, banegasine a chlorinated diketopiperazine derivative **11**, and two lanthipeptides **12** and **13**) exhibit interesting chemical features that are key in many pharmacologically important compound classes.

## 4. Materials and Methods

General procedures: NMR measurements were performed on a Bruker AVANCE III 500 MHz and 600 MHz spectrometer, equipped with a Bruker Cryoplatform. The chemical shifts are reported in parts per million (ppm) relative to the solvent residual peak of DMSO-*d*_6_ (^1^H: 2.50 ppm, quintet; ^13^C: 39.52 ppm, heptet). LC-ESI-HRMS measurements were carried out on an Accela UPLC system (Thermo Scientific) coupled with an Accucore C18 column (100 mm × 2.1 mm, particle size 2.6 µm) combined with a Q-Exactive mass spectrometer (Thermo Scientific) equipped with an electrospray ion (ESI) source. UHPLC-MS measurements were performed on a Shimadzu LCMS-2020 system equipped with single quadrupole mass spectrometer using a Phenomenex Kinetex C18 column (50 mm × 2.1 mm, particle size 1.7 μm, pore diameter 100 Å). The column oven was set to 40 °C; scan range of MS was set to *m*/*z* 150–2000 with a scan speed of 10,000 u/s and event time of 0.25 s under positive and negative mode. The DL temperature was set to 250 °C with an interface temperature of 350 °C and a heat block temperature of 400 °C. The nebulizing gas flow was set to 1.5 L/min and dry gas flow to 15 L/min. Semipreparative HPLC was performed on a Shimadzu HPLC system using a Phenomenex Luna C18(2) 250 mm × 10 mm column (particle size 5 μm, pore diameter 100 Å). IR spectra were recorded on an FT/IR-4100 ATR spectrometer (JASCO). Optical rotations were recorded in methanol on a P-1020 polarimeter (JASCO). Solid phase extraction was carried out using Chromabond C18ec cartridges filled with 2 g and 10 g of octadecyl-modified silica gel (Macherey-Nagel, Düren, Germany). Open column chromatography was performed on Sephadex LH20 (GE Healthcare, Hamburg, Deutschland). Chemicals: Methanol and acetonitrile LC-MS grade (VWR International GmbH, Dresden); water for analytical and preparative HPLC (Millipore, Darmstadt, Germany); formic acid (Carl Roth, Karlsruhe, Germany); acetonitrile (VWR as LC-MS grade); media ingredients (Carl Roth, Karlsruhe, Germany).

Sample collections and isolation procedures: Biological material (soldiers, workers, and fungus comb) was collected from eleven *M. natalensis* nests (stored in 50% glycerol) and one *M. natalensis* and one *Odontotermes* sp. nest for transcriptomic analysis (Table S1) (stored in RNAlater^@^, Sigma Aldrich, St. Louis, MO, USA). Samples were kept on ice immediately after collection and stored at −80 °C within one day. Frozen termite workers (gut and cuticle) and fresh fungus comb material were used for the isolation of Actinobacteria and each sample was processed separately. First, termites and fungus comb samples were individually washed with ddH_2_O (250 µL) and the wash water collected separately for subsequent isolation procedure. Then, major termite workers were surface sterilized with 70% ethanol and washed in sterile Ringer solution (7.5 g/L NaCl, 0.35 g/L KCl, 0.21 g/L CaCl_2_). Termites were dissected using sterile, fine tipped forceps and intact guts were immediately removed and stored in 500 µL PBS on ice until further use (5 guts per sample). Dissected guts were crushed using a sterile pestle and a series of dilution (up to 10^−6^ in PBS) was produced. Bacteria from each sample were isolated by plating 100 µL of each dilution series (10^−4^–10^−6^) on two different selective low-nutrient media: chitin and microcrystalline medium supplemented with 0.05 g/L cycloheximide (Table 2) [17]. Isolates with Actinobacteria-like morphology were transferred to the nutrient-richer medium ISP2 and subcultured. A total of 68 isolates were obtained from gut compartment, 16 isolates from termite cuticle, and 13 isolates from fungus comb (Table S2).

DNA extraction, PCR amplification, pairwise sequence similarities and phylogenetic analysis: Actinobacteria were grown in nutrient-rich ISP2 broth for 5 to 7 days at 30 °C (150 rpm). Cells were harvested, and genomic DNA was extracted using the GenJet Genomic DNA Purification Kit (Thermo Scientific, Waltham, MA, USA, #K0721) following the manufacturer’s instructions with slight changes (lysozyme incubation time 40 min, protein kinase K treatment 40 min). DNA was quantified spectrophotometrically using Nanodrop (Thermo Scientific, Waltham, MA, USA). 16S rRNA gene was amplified using the primers pair 27F/1492R [17]. The amplification reaction was prepared in 25 µL final volume containing: 7.25 µL dH_2_O, 5.0 µL HF buffer, 5.0 µL of each primer (2.5 µM), 0.5 µL dNTPs (10 µM), and 0.25 µL Phusion High Fidelity DNA Polymerase (New England Biolabs). PCR was performed with the conditions: 98 °C for 38 s, followed by 32 cycles of 98 °C for 30 s, 52 °C for 45 s, 72 °C for 1 min 20 s, and a final extension of 72 °C for 8 min. PCR products were visualized by agarose gel electrophoresis. PCR reactions were purified using the PCR Purification Kit (Thermo Scientific, Waltham, MA, USA, #K0702) and sequenced at GATC (Konstanz).

Sequences were checked for purity and mismatches using BioEdit [59]. Forward and reverse sequences of each sequence were assembled with BioEdit and tested for chimeras using DECIPHER [60]. For strains RB9, RB54, RB74, RB85, and RB129 only reverse or forward sequences were generated (Table S3). Resulting sequences were used for a BLASTn search in GenBank using “refseq_rna” database [61]. Pairwise sequence similarities were calculated using the method recommended by Meier-Kolthoff [30] for 16S rRNA gene available via the GGDC web server available at http://ggdc.dsmz.de/. Sequence similarities were calculated for all strains with first three hits (Table S3). A phylogenetic analysis was done with the 16S rRNA sequences (GenBank accession numbers KX344916-KX344918, KY312017-KY312022, KY558669-KY558746, and MH044507-MH044516) and the first hit from the BLASTn search (Figure S1, Table S3). Sequences were aligned with muscle [62] and trimmed using MEGA 7.0.26 [63]. Two different phylogenetic trees were reconstructed with neighbor-joining [64] and maximum likelihood algorithms (Figure S1) [65].

The evolutionary distance model of Kimura or Tamura and Nei was used to generate evolutionary distance matrices for the maximum likelihood [66,67], and neighbor joining algorithm with deletion of complete gaps and missing data. For the maximum-likelihood algorithm, a discrete Gamma distribution was used (+G) and the rate variation model allowed for some sites to be evolutionarily invariable (+I). For the neighbor-joining algorithm rate variation among sites was modeled with a gamma distribution. For all constructed trees the confidence values of nodes were evaluated by bootstrap analysis based on 1000 resamplings [68]. For graphic design, iTOL v3 (https://itol.embl.de/, 31st of July, 2018) was used with the following settings: leaf sorting = none, branch length = ignore, scaling factors: Hor = 0.3, Vert: = 0.8) [69].

Phylogenetic comparison of strain RB108: Near-complete 16S rRNA sequences (1365 bp, GenBank accession number KY558675) were used for a search in NCBI database (reference RNA sequences). The first three hits were *Streptomyces pulveraceus* NBRC 3855, *Streptomyces atratus* NRRL B-16927, and *Streptomyces gelaticus* NRRL B-2928 with an Ident value of 99%. All three hits were phenotypically compared using the Wink compendium [70]. Strain RB108 exhibited a different phenotype compared to the above listed *Streptomyces*. Therefore, the full-length 16S rRNA sequence (1514 bp, GenBank accession number MH828334) was extracted of the genome of strain RB108 and used for comparison. The first three hits of the reference RNA sequence database were *Streptomyces fulvissimus* DSM 40593, *Streptomyces caviscabies* ATCC 51928, and *Streptomyces luridiscabiei* S63. Virtual DDH estimation of strain RB108 and *Streptomyces fulvissimus* DSM 40593 was performed, resulting in a value of 26.20% (23.8–28.7%). According to the DDH threshold of <70% both strains can be regarded as distinct two separate strains [30,31].

Culture extracts: Actinobacteria were cultivated in 25 mL ISP2 or PDB for 4 days at 30 °C at 150 rpm, after 4 days additional 25 mL ISP2 or PDB broth were added and cultivation was continued for another 3 to 5 days. Cultures were centrifuged (6000 rcf, 10 min) and the resulting cell pellets were lysed using MeOH (9 mL). The resulting methanolic cell extracts were combined with the culture supernatant to yield a 20% MeOH culture supernatant. Metabolites from the supernatant were concentrated using an activated (20% MeOH) Chromabond C18ec cartridges filled with 500 mg of octadecyl-modified silica gel (Macherey-Nagel, Düren, Germany). (Unless stated otherwise: %MeOH refers to a mixture of MeOH and dH_2_O). Metabolites were eluted using 100% MeOH (5 mL) and 100% acetone (2 mL) and pooled and concentrated in vacuo. The resulting extracts (E) were adjusted with MeOH to 1 mg/mL and used for bioactivity assays.

Antimicrobial activities against test strains: Antimicrobial assays against *Bacillus subtilis* ATCC 6633, *Staphylococcus aureus* IMET 10760, *Escherichia coli* SG 458, *Pseudomonas aeruginosa* K799/61, *Mycobacterium vaccae* IMET 10670, *Sporobolomyces salmonicolor* SBUG 549, *Candida albicans* BMSY 212, and *Penicillium notatum* JP36 were done using the broth dilution method according to the NCCLS (National Committee for Clinical Laboratory Standards) (Table S5).

Antifungal activity assay against co-isolated fungi: All fungal isolates (Table S4) were cultivated on PDA plates for a maximum of six weeks (23 °C) and subcultured by plating mycelium-containing agar pieces (1 cm × 1 cm) onto fresh PDA. To evaluate antifungal activity, a filter paper disk (d = 6 mm) was soaked with 10 µL extract (1 mg/mL) and dried (sterile air flow). Depending on the growth behavior of each fungus, two different assays were applied. Method A (fast-growing fungi), mycelium covered agar pieces were placed in the middle of a PDA plate (standard petri dish 92 mm × 16 mm) and sterile filter paper discs were placed at a distance of 1–2 cm from each agar plug. Method B (slow to medium-fast growing fungi): Fungi were grown in 25 mL PDB for 10 to 14 days at 30 °C (150 rpm) and 500 µL of actively growing culture or a spore solution (*M. anisopliae*) was used to inoculate a PDA plate (standard petri dish 92 mm × 16 mm). Distribution of mycelium or spores on plates was performed using sterile glass beads. Plates were dried and filter discs soaked with equal amounts (10 µL) of extracts were put onto the PDA plate. Plates were checked daily and the diameter of the zone of inhibition (ZOI) (no growth, mycelium free) was recorded as a measure of inhibition (Tables S6 and S8). Amphotericin B (8 mg/mL in DMSO) and cycloheximide (50 mg/mL in MeOH) were used as positive controls, 100% MeOH was used as a negative control. All combinations were prepared in duplicate.

Co-cultivation studies: *Streptomyces* sp. RB108 was grown for 14 days at 30 °C in ISP2, 25 µL of liquid culture were used to inoculate ISP2 and PDA plates centrally. Plates were incubated for 7 days at 30 °C until a clear colony (1 cm in diameter) was apparent. Then, plates were inoculated at the edge of the agar plate with two agar pieces covered with fungal mycelium (Table S9). All combinations were prepared in triplicate. Amphotericin B (8 mg/mL in MeOH) was used as positive control; ddH_2_O was used as negative control. Plates were incubated for 10 days at room temperature Plates were checked daily for the formation of a zone of inhibition (ZOI). When a clear, stable ZOI was detectable (normally after 10 days), the ZOI was cut out and extracted with 100% methanol (overnight). Methanol extracts were dried and stored at −20 °C until further use and then subjected to comparative LC-MS and HPLC analysis. Due to significant upregulation of metabolites during co-cultivation, the combination of *Streptomyces* sp. RB108 and fungus #4 was selected for subsequent experiments.

Isolation and structural elucidation of barceloneic acid A (7): For isolation of upregulated metabolites, large scale co-cultivation of *Streptomyces* sp. RB108 and *Pleosporales* sp. #4 was accomplished by inoculation of 30 PDA agar plates (92 mm × 16 mm, 20 mL agar/plate) as described above. Each ZOI was excised from the plate then pooled and extracted with MeOH overnight and solvent removed by evaporation. The resulting crude extract (adjusted to 10% MeOH) was loaded on an activated and equilibrated SPE C18 column and fractionated by step-gradient from 10% MeOH to 100% MeOH (100 mL each). The 50% MeOH eluate was separated by semipreparative HPLC to yield pure compound 7 (*m*/*z* 319.19 [M − H]^−^; 302.95 [M + H − H_2_O]^+^). The molecular formula of barceloneic acid A (**7**) was assigned as C_16_H_16_O_7_ based on ESI-HRMS (*m*/*z* 321.0964 [M + H]^+^, calcd. 321.0969 Δ = −1.46 ppm) (Figure S11). The ^1^H NMR analysis (Table S12) revealed 13 protons as sharp signals, suggesting additional three invisible exchangeable protons. Two *meta*-substituted aromatic moieties could be deduced according to the ^1^H chemical shifts and multiplicity of protons at δ_H_ 6.23 ppm (1H, doublet, *J* = 2.7 Hz, H-11) and δ_H_ 6.44 ppm (1H, doublet, *J* = 2.7 Hz, H-13); δ_H_ 5.95 ppm (1H, broad singlet, H-4) and δ_H_ 6.24 ppm (1H, broad singlet, H-6), respectively. The methyl group at δ_H3-8_ 2.07 ppm/δ_C-8_ 21.2 ppm was deduced to attach on aromatic ring based on the HMBC correlations of H_3_-8 to C-4/C-5/C-6 and H-4 to C-1/C-3/C-5/C-6/C-8 and H-6 to C-1/C-4/C-7/C-8. The oxygenated methyl moiety at δ_H3-16_ 3.68 ppm/δ_C-16_ 54.9 ppm was suggested to connect on second aromatic ring based on the HMBC correlation of H_3_-16 to C-12. The oxygenated methylene moiety at δ_H-15_ 4.51 ppm (2H, doublet, *J* = 1.2 Hz, H-15)/δ_C-15_ 58.3 ppm was deduced to connect on the same aromatic ring based on the HMBC correlations of H_2_-15 to C-9/C-13/C-14, H-13 to C-12/C-14/C-15, and H-11 to C-9/C-10/C-12/C-13. Finally, the proposed structure of barceloneic acid A (**7**) matched the literature reported HRMS and 2D NMR data (Table S13) [35]. NMR spectra are shown in Figure S7–S10.

MS Imaging: An indium tin oxide (ITO) coated glass slide was used for Imaging MS (Bruker Daltonics, Billerica, MA, USA) and covered with 1 mL of ISP2 and PDA medium, respectively. The dried agar glass slide was then inoculated with 10 μL of a *Streptomyces* sp. RB108 liquid culture (middle of the cover slide) and incubated for 7 d at 30 °C. Two square agar pieces (0.3 cm × 0.3 cm) covered with mycelium of fungus # 4 were arranged at a distance of 1cm on the cover slide (Figure S2). After an incubation period of 7 days at RT, the slides were dried for an additional half hour next to an open flame and then sprayed with a saturated solution (7 g/L) of universal MALDI matrix (1:1 mixture of 2,5-dihydroxybenzoic acid and α-cyano-4-hydroxy-cinnamic acid; Sigma Aldrich) in acetonitrile HPLC grade, using the automatic system ImagePrep device 2.0 (Bruker Daltonics, Bremen, Germany). The sample was analyzed in an UltrafleXtreme MALDI TOF/TOF (Bruker Daltonics, Bremen, Germany), which was operated in positive reflector mode using flexControl 3.0 (Bruker, Bremen, Germany). The analysis was performed in the 100–3000 and 400–4000 Da ranges, with 40% laser intensity (laser type 3), accumulating 1000 shots by taking 50 random shots at every raster position. Raster width was set at 150 µm. Spectra were processed with baseline subtraction in flexAnalysis 3.3 (Bruker, Bremen, Germany). Processed spectra were uploaded in flexImaging 3.0 for visualization and SCILS Lab 2015b for analysis and representation. Chemical images were obtained after peak alignment on the dataset using Median normalization and weak denoising.

Isolation and structure elucidation of banegasine (**10**): Large-scale cultivation of *Actinomadura* sp. RB29 was performed by inoculation of 100 ISP2 agar plates (standard 150 mm × 20 mm, 40 mL ISP2 agar/plate) at 30 °C for 10 days. Whole agar plates were cut into pieces and extracted twice with 2 L MeOH (1% AcOH) at 4 °C overnight. MeOH/AcOH extracts were filtered and concentrated under reduced pressure. The crude extract was dissolved using 10% MeOH and loaded on an activated and equilibrated SPE C18 column (10 g) and fractionated by step-gradient from 10% MeOH to 100% MeOH (100 mL each). The eluent using 30% MeOH was first purified by Sephadex LH20 using 50% MeOH to obtain subfractions Fr.3.1–Fr.3.6 (20 mL/Fr.). Concentrated violet band Fr.3.6 was further separated by semipreparative HPLC to yield pure banegasine (**10**, 2.0 mg, *t*_R_ = 6.74 min) using the following gradient: 0–5 min, 50% B; 5–20 min, 50–100% B; 20–25 min, 100% B (A: dd H_2_O + 0.1% formic acid; B: MeOH) with a flow rate of 2.0 mL/min. The molecular formula of banegasine (**10**) was assigned as C_11_H_12_O_2_N_2_ based on ESI-HRMS (*m*/*z* 205.0972 [M + H]^+^, calcd. 205.0972 Δ = 0.13 ppm) and λ_max_ 217,.278 nm (MeCN/H_2_O/FA) (Figure S13). The ^1^H NMR analysis (Table S14) revealed nine protons as sharp signals, suggesting additional three invisible exchangeable protons. The *ortho*-substituted aromatic moiety was deduced from the ^1^H chemical shifts and multiplicity of protons at δ_H_ 7.35 ppm (1H, doublet, *J* = 8.1 Hz, H-3′); δ_H_ 7.06 ppm (1H, triplet, *J* = 7.8 Hz, H-4′); δ_H_ 6.97 ppm (1H, triplet, *J* = 7.2 Hz, H-5′); δ_H_ 7.56 ppm (1H, doublet, *J* = 7.9 Hz, H-6′). The dihydropyrrole moiety was deduced from the aliphatic protons at δ_H_ 3.48 ppm (1H, doublet of doublet, *J* = 8.6, 3.9 Hz, H-2) and δ_H_ 2.99 ppm (1H, doublet of doublet, *J* = 15.1, 8.9 Hz, H-3a) and δ_H_ 3.31 ppm (1H, doublet of doublet, *J* = 15.1, 3.7 Hz, H-3b), and olefinic proton at δ_H_ 7.22 ppm (1H, doublet, *J* = 1.2 Hz, H-5). ^1^H NMR spectrum of banegasine is shown in Figure S12.

Isolation and structure elucidation of *cyclo*(*N*Me-L-3,5-dichlorotyrosine-Dhb) (**11**): Large-scale liquid cultivation of *Actinomadura* sp. RB29 was performed in a 50 L fermenter (20 L Soya Broth liquid media, pH 6.8) for 5 days at 28 °C (stirring). The culture supernatant was separated from biomass by separator and collected and loaded onto activated XAD16 resin (1 kg). The resin was first washed by water (2 L) and then eluted by MeOH/H_2_O mixture in a step gradient manner, with 1 L 10% MeOH, 30% MeOH, 50% MeOH, 80% MeOH, 100% MeOH, respectively. The corresponding eluates were concentrated under reduced pressure and redissolved into 50% MeOH or 100% MeOH as 5 mg/mL for standard metabolomic LCMS analysis. The interesting ion with *m*/*z* at 328.9 ([M+H]^+^) was redetected from 80% MeOH eluate and allowed us to submit this 80% MeOH eluate onto Sephadex LH20 resin and eluted by 100% MeOH for further purification. Subfractions containing the ion *m*/*z* at 328.9 ([M+H]^+^) were concentrated under reduced pressure and finally purified by semipreparative HPLC (Nucleodur C18 250 mm × 10 mm) to yield compound **11** (2.0 mg, *t*_R_ = 13.33 min) using the following gradient: 0–5 min, 30% B; 5–25 min, 30–83% B; 25–30 min, 100% B (A: dd H_2_O + 0.1% formic acid; B: MeCN) with a flow rate of 2.0 mL/min. The molecular formula of compound (**11**) was determined as C_14_H_14_O_3_N_2_Cl_2_ based on ESI-HRMS analysis (*m*/*z* 329.04520 [M + H]^+^, calcd. 329.04542 Δ = −0.68 ppm) and the isotope abundance of chlorine, and further confirmed by the observation of 14 carbon atoms from ^13^C-NMR and DEPT135 spectra analysis (Figure S15 and S16). Detailed analysis of 1D and 2D NMR spectra (Figure S14, S17–S20) indicated two carbonyl groups, one four-substituted benzyl moiety, one olefinic moiety, one methylene and one methine group, two methyl groups, and one visible OH/NH signal. The presence of two amide groups (δ_C-1_ 165.5 ppm/δ_C-9_ 159.0 ppm) suggested the amino acids origin. Methylated olefin moiety was deduced from the ^1^H-^1^H COSY correlation of H-11 (δ_H_ 5.41 ppm/δ_C_ 112.0 ppm) to H_3_-12 (δ_H_ 1.46 ppm/δ_C_ 10.7 ppm) and the HMBC correlations of H-11 to C-12, and H_3_-12 to C-10 and C-11. The further HMBC correlations of H-11 and H_3_-12 to C-9 (δ_C_ 159.0 ppm), OH/NH (δ_H_ 9.92 ppm) to C-9 and C-10 suggested the possible dehydroaminobutyric acid (dhAbu or Dhb) moiety. Second spin system C-2–C-3 from COSY correlation of H-2 to H_2_-3 and the aromatic protons (δ_H-5_ 6.94 ppm/δ_C-5_ 129.7 ppm), and *N*-methyl group (δ_H3-8_ 2.94 ppm/δ_C3-8_ 32.1 ppm) were deduced to belong to the substituted tyrosine skeleton based on the deeper observation of HMBC correlations of H-2 to C-1/-3/-4/-8, and H_2_-3 to C-1/-2/-4/-5, and H-5 to C-2/-3/-6/-7, and H_3_-8 to C-2. Based on the ^13^C chemical shift compared with the literature [70] and demand of elemental composition, two chlorine atoms were suggested to attach on the C-6 individually, leading to the build-up of *N*-methyl 3,5-dichlorotyrosine moiety. The diketopiperazine structure condensed from Dhb and *N*-methyl 3,5-dichlorotyrosine residue was deduced by the HMBC correlations of *N*H to C-1/-2, and H-2 to C-9, and H_3_-8 to C-9. Furthermore this conclusion was confirmed by the observation of MS fragmentation of *m*/*z* 329.04520 ([M+H]^+^) (Figure S21) and prediction by Mass Frontier 7.0 (Thermo, Figure S22). The fragment ion at *m*/*z* 301.05118 (C_13_H_15_N_2_O_2_Cl_2_^+^) might originate from the diketopiperazine ring opening and loss of carbonyl group; ion at *m*/*z* 218.01393 (C_9_H_10_NOCl_2_^+^) might derive from the diketopiperazine ring opening and loss of carbonyl group and Dhb moiety; and ion at *m*/*z* 174.97168 (C_7_H_5_OCl_2_^+^) might represent the (3,5-dichloro-4-hydroxyphenyl)methylium and the ion at *m*/*z* 127.08707 (C_6_H_11_N_2_O^+^) might deduce from the Dhb moiety and *N*-methyl group with rearrangement (e.g., 1-(2-iminobutanoyl)aziridin-1-ium). The NOESY correlation of H_3_-12 to NH suggested the configuration of double bond from Dhb moiety as *Z* form. Finally the planar structure of **11** was suggested as *cyclo*(dehydroaminobutyric acid- *N*-methyl 3,5-dichlorotyrosine). Comparison of the specific optical rotation of **11** with reported tyrosine or phenylalanine containing diketopiperazine [71,72], the stereochemistry of *N*-methyl 3,5-dichlorotyrosine moiety (–104°) was deduced to be L-configurated. 

*cyclo*(*N*-Me-L-3,5-dichlorotyrosine-Dhb) (**11**): light yellow solid; [α${]}_{D}^{25}$ –104.0 (c 0.1 *w*/*v*%, MeOH); UV (MeCN/H_2_O/FA) λ_max_ 230 nm; IR (ATR) ν_max_ 3857, 3738, 3650, 2925, 2855, 1745, 1683, 1650, 1539, 1512, 1460, 674 cm^–1^; NMR spectral data, see Table S15; ESI-HRMS [M+H]^+^ *m*/*z* 329.04520 (calcd for C_14_H_15_O_3_N_2_Cl_2_, 329.04542). A SCIFinder search indicated the commercial availability from Aurora Screening Library (CAS 1798281-02-3) however the natural origin of the compound is unassigned.

Antimicrobial activities of *cyclo*(*N*-Me-L-3,5-dichlorotyrosine-Dhb) against test strains: Antimicrobial assays against *Bacillus subtilis* ATCC 6633, *Staphylococcus aureus* IMET 10760, *Escherichia coli* SG 458, *Pseudomonas aeruginosa* SG137, *Pseudomonas aeruginosa* K799/61, MRSA *Staphylococcus aureus* 134/93, VRSA *Enterococcus faecalis* 1528, *Mycobacterium vaccae* IMET 10670, *Sporobolomyces salmonicolor* SBUG 549, *Candida albicans* BMSY 212, and *Penicillium notatum* JP36 were done using the broth dilution method according to the NCCLS (National Committee for Clinical Laboratory Standards) (Table S7).

Isolation and structure elucidation of rubromidin A (**12**) and B (**13**): Large-scale liquid cultivation was performed as mentioned above. SPE 70% and 80% MeOH C18-SPE eluates, which containing ion with *m*/*z* at 957.8 ([M+2H]^2+^) and 993.5 ([M+2H]^2+^).

Those two fractions were concentrated under reduced pressure and pooled and resubmitted to Sephadex LH20 resin and eluted by 100% MeOH for further purification. The subfractions containing ion with *m*/*z* at 957.8 ([M + 2H]^2+^) and 993.5 ([M + 2H]^2+^) were concentrated under reduced pressure and finally purified by semipreparative HPLC (Nucleodur C18 250 mm × 10 mm) to yield rubromidin A (**12**, 0.5 mg, *t*_R_ = 10.02 min) and rubromidin B (**13**, 0.5 mg, *t*_R_ = 10.80 min) using the following gradient: 0–20 min, 30% B (A: dd H_2_O + 0.1% formic acid; B: MeCN) with a flow rate of 2.0 mL/min. ESI-HRMS analysis of purified lanthipeptide **12** revealed the protonated molecular ion at *m*/*z* 1914.73938, as well as the doubly protonated ion at *m*/*z* 957.87408 (Figure S23). The MS^2^ spectra at *m*/*z* 1914.73938 and 957.87408 under positive mode were recorded and submitted to GNPS and processed by RiPPquest, a tandem mass spectrometry database search tool for identification of microbial RiPPs [73]. The exact mass of protonated ion of compound **13** was assigned with *m*/*z* 1985.77539, by the observation of the doubly protonated ion at *m*/*z* 993.39203 (Figure S24). The mass difference of 71.03601 between **12** and **13** suggested an additional alanine residue from the *N*-terminus. Similarly, the MS^2^ spectra at *m*/*z* 1985.77539 and 993.39203 were recorded and submitted to GNPS and processed by RiPPquest, and led to the identification of the candidate peptide (ACSSTCTSGPFTFACDGTTKG), including an additional alanine which might due to the alternative cleavage of RumM.

Marfey’s reaction [74]: Compounds **12** and **13** (0.1 mg each) were hydrolyzed separately by 6 N HCl (1.0 mL) at 110 °C for 15 h. Then HCl was removed using a SpeedVac and 20 μL FDAA (1-fluoro-2,4-dinitrophenyl-5-l-alanine amide, 10 mg/mL in acetone) and 100 μL NaHCO_3_ (1 N aqueous solution) were added. The reaction was heated at 80 °C for 10 min, and the reaction quenched by addition of 50 μL 2 N HCl. L- and D-phenylalanine and L- and D-alanine, glycine, L- and D-serine, L- and D-theorine, and L- and D-proline were converted accordingly. After centrifugation for 10 min at 13,000 rpm, the reaction mixture was analyzed by UHPLC-MS (Figures S29 and S30). Five μL of the reaction mixture were injected and analyzed using the following gradient: 0–1 min, 10% B; 1–7 min, 10–100% B; 7.1–10 min, 100% B (A: dd H_2_O with 0.1% formic acid; B: MeCN with 0.1% formic acid) at a flow rate of 0.7 mL/min.

Ser/Cys labeling: *Actinomadura* sp. RB29 was first incubated in 50 mL ISP2 at 30 °C (150 rpm shaking) for one week. Then, biomass was collected by centrifugation (4000 rpm, 10 min, rt), washed twice using autoclaved minimal media and transferred into 100 mL minimal media containing L-serine-2,3,3-D_3_ (100 mg) and DL-cystein-3,3-D_2_ (100 mg), respectively. The cultures were incubated for one week at 30 °C (150 rpm). The culture broth was collected by filtration and extracted with activated HP20 resin (20 g/L) at 4 °C overnight. The resin was washed by H_2_O firstly then eluted by 100% MeOH. The MeOH eluate was concentrated under reduced pressure and resuspended into MeOH for LCMS analysis and ESI-HRMS analysis (Figure S24–27).

Gene cluster identification: The putative biosynthetic gene cluster of rubromidin (*rum*) was predicted using antiSMASH and compared with already described lantipeptide gene cluster *cin* (from *Streptomyces cinnamoneus*) and putative biosynthetic gene cluster of cinnamycin B from *Actinomadura atramentaria* [39,40,75] (Figure S6, Table S11). The peptide sequence of lantibiotic cinnamycin precursor (predicted with antiSMASH) was used for al BLASTp search in GenBank using “refseq protein” or “nonredundant protein sequence” database. A phylogenetic analysis was performed using lantibiotic cinnamycin precursor peptide sequence (GenBank accession number WP 103565569) and LanM lanthipeptide synthetase (GenBank accession number WP 103565568) and related hits from the BLASTp search. Phylogentic trees are shown in Figures S3 and S4. Comparative sequence alignment of precursor peptide sequence is shown in Figure S5. Sequences were aligned with Muscle [62]. Two different phylogenetic trees were reconstructed with neighbor-joining and maximum-likelihood algorithms [63,64]. The evolutionary distance model of Jones, Taylor, and Thorton [76] was used to generate evolutionary distance matrices for the maximum likelihood and neighbor joining algorithm with deletion of complete gaps and missing data. For the maximum-likelihood algorithm, a discrete Gamma distribution was used (+G). For the neighbor-joining algorithm, the rate variation among sites was modeled with a gamma distribution. For all constructed trees the confidence values of nodes were evaluated by bootstrap analysis based on 1000 resamplings. 

## 5. Conclusions

This study provides an extended taxonomical and chemical analysis of Actinobacteria isolated from the fungus-growing termite *M. natalensis*. Our findings clearly verify that a high diversity of Actinobacteria can be found with these termites, and most notably the termite gut. The high recovery rates from gut fluids suggest that most species survive the relatively short passage and are inoculated together with the fungal mutualists into the fresh fungus comb. Although the high phylogenetic diversity denotes a certain lack of specificity between bacteria and origin of isolation, it simultaneously ensures the presence of diverse chemistry and biochemical capacity, which may be necessary for protection against alien species and the assisted breakdown of complex plant material. It also needs to be noted that antibiotics at higher concentrations might be deployed as weapons against competing microbes; they also have been found to elicit changes in the global bacterial transcription patterns and metabolism at sub-inhibitory concentrations [77,78]. Therefore, it could be speculated that natural products, including antibiotics, serve as signaling molecules between different microorganisms within the termite symbiosis and contribute to the overall unique stability of the system. Consequently, the structural diversity of metabolites produced within the mutualistic agricultural system might provide a net benefit for the fungal mutualist and farming termites; however, documenting the individual compounds and costs and benefits involved in this potential mutualism are still required. 

An intriguing feature of studying the defensive symbiosis paradigm is its parallel to human medicine as it both deploys mediating or antagonistic molecules to suppress pathogens. Insect defensive symbioses, such as the fungus-growing termite systems, probably offer the clearest window into antibiotic use in nature, and the presence of natural product factories, such as Actinobacteria, represent an untapped chemical treasure trove of novel chemical scaffolds, a fact that is underlined by the impressive amount of new natural product scaffolds derived from termite-associated bacteria reported in the last decade. However, key to the discovery of new natural products is the ability to mimic the natural environment and the dynamics present within the system. As exemplified in our two case studies, co-culture assays are a first step towards mimicking natural habitats and identifying key secondary metabolites from both bacteria and fungi. However, more efforts are clearly needed to steadily increase the levels of complexity and more holistically describe the metabolic network within the symbiosis.

## Figures and Tables

**Table 1:** The number of Actinobacteria isolated within this study and by Visser et al. [17], and their origin of isolation by genera and family.

Genus	Family	This Study: Origin of Isolation (Number of Isolates)			Visser et al. [17] Origin of Isolation (Number of Isolates)	
		Termite Gut	Termite Exoskeleton	Fungus Comb	Termite Exoskeleton	Fungus Comb
*Streptomyces*	Streptomycetaceae	46	14	13	13	2
*Kitasatospora*	Streptomycetaceae	0	0	0	2	0
*Actinomadura*	Thermomonosporaceae	4	1	0	1	0
*Leifsonia*	Microbacteriaceae	3	0	0	0	0
*Curtobacterium*	Microbacteriaceae	1	0	0	0	0
*Arthrobacter*	Micrococcaceae	0	1	0	0	0
*Micromonospora*	Micromonosporaceae	3	0	0	1	1
*Nocardia*	Nocardiaceae	3	0	0	0	0
*Aeromicrobium*	Nocardioidaceae	1	0	0	0	0
*Cellulosimicrobium*	Promicromonosporaceae	1	0	0	0	0
*Mycobacterium*	Mycobacteriaceae	3	0	0	0	0
*Sphaerisporangium*	Streptosporangiaceae	1	0	0	0	0
*Microbispora*	Streptosporangiaceae	1	0	0	0	0
*Luteimicrobium*	family of order *Micrococcales*	1	0	0	0	0

**Table 2:** Media compositions used for initial isolations and subsequent growth assays and large-scale cultivation.

Medium (Abbreviation)	Content Per L
Potato Dextrose Broth (PDB)	26.5 g potato extract glucose (6.5 g potato extract, 20 g glucose)
Potato Dextrose Agar (PDA)	26.5 g potato extract glucose, 20.0 g agar
ISP2 Broth	4.0 g yeast extract, 10.0 g malt extract, 4.0 g glucose
ISP2 Agar	4.0 g yeast extract, 10.0 g malt extract, 4.0 g glucose, 20.0 g agar
Chitin Agar	4.0 g chitin, 0.7 g K_2_HPO_4_, 0.3 g KH_2_PO_4_, 0.57 g MgSO_4_·7H_2_O, 0.01 g FeSO_4_·7H_2_O, 0.0018 g ZnSO_4_·7H_2_O, 0.0016 g MnCl_2_·4H_2_O
Minimal Media	2.0 g Na-acetate, 2.0 g NH_4_Cl 0.7 g K_2_HPO_4_, 0.3 g KH_2_PO_4_, 0.57 g MgSO_4_·7H_2_O, 0.01 g FeSO_4_·7H_2_O, 0.0018 g ZnSO_4_·7H_2_O, 0.0016 g MnCl_2_·4H_2_O
MC Agar	5.0 g microcrystalline cellulose, 20.0 g agar
Soya Broth	20.0 g soya flour, 20.0 g glucose, 5.0 g NaCl, 3.0 g CaCO_3_, 0.25 mL desmophen

## Supplementary Materials

The following are available online at http://www.mdpi.com/2079-6382/7/3/83/s1: Table S1: Colonies information of *M. natalensis* for Actinobacteria isolation and metatranscriptomic analysis, Table S2: Actinobacteria IDs of isolates from fungus-growing termites, Table S3: Identities of isolated Actinobacteria strains, Table S4: Identities of ecologically-relevant fungal strains used as targets in the bioactivity tests, Table S5: Antimicrobial assay results of extracts of isolated bacteria against eight medically relevant bacteria and fungi, Table S6: Antifungal assay results of extracts of isolated bacteria against ecologically-relevant fungi, Table S7: Antimicrobial assay results of *cyclo*(*N*-Me-L-3,5-dichlorotyrosine-Dhb) (**11**), Table S8: Representative images of antifungal assay of extract against ecologically relevant fungi, Table S9: Representative interactions between *Streptomyces* sp. RB108 and co-cultivated fungi, Table S10: Presence/absence of transcripts identified in gut microbiome metatranscriptome data from major old worker guts of *M. natalensis* Mn156 and Odontotermes sp. Od127, Table S11: Rubromidin biosynthetic protein annotations based on sequence homology, Tables S12–S15: NMR tables, Figure S1: Unrooted Neighbor-joining tree based on near-complete 16S rRNA gene sequences showing relationship**s** between isolated Actinobacteria and closest relatives, Figure S2: MALDI-TOF imaging of co-cultivation of RB108 and *Pleosporales* sp. #4, Figure S3: Neighbor-joining tree based on peptide sequence of precursor peptide sequence (RumA), Figure S4: Neighbor-joining tree based on type 2 lantipeptide synthetase RumM, Figure S5: Comparative sequence alignment of precursor peptide sequence, Figure S6: Comparative gene maps of *rum* and *cin* and proposed cinnamycin B biosynthetic clusters, Figures S7–S30: NMR spectra and LCMS of Marfey’s reaction.
