# Supplementary material for: Natural Products from Actinobacteria Associated with Fungus-Growing Termites
Source: Antibiotics (Basel). 2018 Sep 13;7(3):83. doi: 10.3390/antibiotics7030083 (PMC6165096; doi:10.3390/antibiotics7030083)
Supplement: Supplementary file 1 [file antibiotics-07-00083-s001.pdf]

Natural products from Actinobacteria associated with fungus-growing termites

René Benndorf,[1] Huijuan Guo,[1] Elisabeth Sommerwerk,[1] Christiane Weigel,[1] Maria Garcia-Altares,[1] Karin Martin,[1] Haofu Hu,[2] Michelle Küfner,[1] Z. Wilhelm de Beer,[3] Michael Poulsen,[2] Christine Beemelmanns[1*]

[1]Leibniz Institute for Natural Product Research and Infection Biology – Hans-Knöll-Institute, Beutenbergstraße 11a, 07745 Jena, Germany; rene.benndorf@leibniz-hki.de, huijuan.guo@leibniz-hki.de, e.sommerwerk@gmail.com, christiane.weigel@leibniz-hki.de, huijuan.guo@leibniz-hki.de, maria.garcia-altares@leibniz-hki.de, karin.martin@leibniz-hki.de michelle.kuefner@gmx.de

[2]Section for Ecology and Evolution, Department of Biology, University of Copenhagen, 2100 Copenhagen East, Denmark; Haofu.Hu@bio.ku.dk, MPoulsen@bio.ku.dk

[3]Department of Microbiology and Plant Pathology, Forestry and Agriculture Biotechnology Institute, University of Pretoria, 0001 Pretoria, South Africa; wilhelm.debeer@fabi.up.ac.za

*Correspondence: christine.beemelmanns@hki-jena.de; Tel.: +49 3641 532-1525

Table of Content

TABLE S1. INFORMATION OF COLONIES OF *MACROTERMES NATALENSIS* (MN) USED FOR ISOLATION OF ACTINOBACTERIA FOR METATRANSCRIPTOME DATA (INCLUDING ONE *ODONTOTERMES* COLONY [OD]), ALONG WITH THEIR GEOGRAPHIC LOCATIONS AND YEAR OF COLLECTION. 4

TABLE S2. STRAINS ISOLATED FROM FUNGUS-GROWING TERMITES, INCLUDING THE MEDIUM THEY WERE INITIALLY ISOLATED ON, THEIR ID AND THE ID OF THE CHEMICAL EXTRACT. 5

TABLE S3. IDENTITIES OF ISOLATED ACTINOBACTERIAL STRAINS, INCLUDING THE TOP THREE HITS RESULTING FROM BLASTN SEARCHES AGAINST THE NCBI DATABASE (HTTPS://BLAST.NCBI.NLM.NIH.GOV/BLAST.CGI, LAST VISIT 26.07.2018, 00:58 AM). 8

TABLE S4. ECOLOGICALLY RELEVANT FUNGAL S USED AS TARGETS IN THE BIOACTIVITY TESTS. 17

TABLE S5. ANTIMICROBIAL ASSAY RESULTS OF EXTRACTS OF ISOLATED BACTERIA AGAINST EIGHT MEDICALLY RELEVANT BACTERIA AND FUNGI (AVERAGE ZONE OF INHIBITION GIVEN IN MM). 18

TABLE S6. ANTIFUNGAL ASSAY RESULTS OF EXTRACTS OF ISOLATED BACTERIA AGAINST ECOLOGICALLY-RELEVANT FUNGI # 1 - #24 (T115: *TERMITOMYCES* SP. FROM *MACROTERMES NATALENSIS* (MN) COLONY MN115, T112: *TERMITOMYCES* SP. FROM *ODONTOTOTERMES BADIUS* COLONY OD112, T153: *TERMITOMYCES* SP. FROM MN COLONY MN153, P5: *TERMITOMYCES* SP. FROM MN COLONY MN105, MA - *METARHIZIUM ANISOPLIAE* ATCC 24942, BB *BEAUVERIA BASSIANA* ST 17960- (I= VERY WEAK INHIBITION, WITHOUT MEASURABLE ZONE OF INHIBITION SI= STRONG INHIBITION, INHIBITION ZONE NOT MEASURABLE; AVERAGE IN MM GIVEN). 22

TABLE S7. ANTIMICROBIAL ACTIVITY OF *CYCLO*(*N*ME-L-3,5-DICHLOROTYROSINE-DHB) (11, 0.1 MG/ML IN MEOH), CIPROFLOXACIN (CIP.) AND AMPHOTERICIN B (AMP.) TOWARDS GRAM (+) AND GRAM (–) BACTERIA AND FUNGI.[A] 23

TABLE S8. REPRESENTATIVE IMAGES OF ANTIFUNGAL PAPER DISC ASSAY AGAINST CO-ISOLATED FUNGI, THE FUNGAL CULTIVAR AND INSECTPATHOGENIC FUNGI. 24

TABLE S9. REPRESENTATIVE CO-CULTIVATION OF *STREPTOMYCES* SP. RB108 AGAINST CO-ISOLATED FUNGI. 27

TABLE S10. PRESENCE/ABSENCE OF TRANSCRIPTS IDENTIFIED IN GUT MICROBIOME METATRANSCRIPTOME DATA FROM MAJOR OLD WORKER GUTS OF *M. NATALENSIS* MN156 AND *ODONTOTERMES* SP. OD127.[A] 32

TABLE S11. RUBROMIDIN BIOSYNTHETIC PROTEIN ANNOTATIONS BASED ON SEQUENCE HOMOLOGY. 34

TABLE S12. NMR DATA (500 MHZ, DMSO-*D*$_6$, AT 300 K) FOR BARCELONEIC ACID A (**7**). 37

TABLE S13. NMR DATA COMPARISON WITH REPORTED AND ISOLATED BARCELONEIC ACID A (**7**). 37

TABLE S14. NMR DATA COMPARISON WITH REPORTED AND ISOLATED BANEGASINE (**10**). 38

TABLE S15. NMR DATA (DMSO-*D*$_6$, AT 300 K) FOR *CYCLO*(*N*ME-L-3,5-DICHLOROTYROSINE-DHB) (11).[A] 38

Figure S1. Unrooted Neighbour-joining tree based on near-complete 16S rRNA gene sequences showing relationships between isolated Actinobacteria and closest relatives. Hashtags indicate branches that were also recovered in the maximum-likelihood tree. Only bootstrap values above 50% (based on 1000 pseudoreplicates) are shown. The scale bar indicates 0.01 substitutions per nucleotide position. 31
Figure S2. Indium tin oxide (ITO) coated glass slide with 1 mL of ISP2 medium and inoculated with A) *Streptomyces* sp. RB108 (after 7 days); B) *Pleosporales* sp. #4; C). co-culture of *Streptomyces* sp. RB108 and *Pleosporales* sp. #4 ; D) optical picture of the challenge assay between *Streptomyces* sp. RB108 and *Pleosporales* sp. #4 after drying. E) Average MS spectra of the MALDI Imaging MS analysis (TIC normalization). F) Zoom in the region of the average MS spectra between *m/z* 1700-2500 showing the up-regulated RiPPs. G) Zoom in the region of the average MS spectra between *m/z* 2100-2300 showing the up-regulated RiPPs. 33
Figure S3. Neighbour-joining tree based on peptide sequence of precursor peptide sequence (RumA, WP 103565569).Star indicate branches that were also recovered in the maximum-likelihood tree. Only bootstrap values above 50% (based on 1000 pseudoreplicates) are shown. The scale bar indicates 0.2 substitutions per amino acid position. 35
Figure S4. Neighbour-joining tree based on type 2 lantipeptide synthetase sequence RumM. Star indicates branches that were also recovered in the maximum-likelihood tree. Only bootstrap values above 50% (based on 1000 pseudoreplicates) are shown. The scale bar indicates 0.5 substitutions per amino acid position. 35
Figure S5. Comparative sequence alignment of precursor peptide sequence (RumA, WP_103565569). 36
Figure S6. Comparative gene maps of *rum* and *cin* biosynthetic clusters and proposed cinnamycin B biosynthetic cluster.. 36
Figure S7. ^{1}H NMR spectrum of barceloneic acid A (**7**) (DMSO-*d*$_6$, 300K, 500 MHz). 39
Figure S8. COSY spectrum of barceloneic acid A (**7**) (DMSO-*d*$_6$, 300K, 500 MHz). 39
Figure S9. HSQC NMR spectrum of barceloneic acid A (**7**) (DMSO-*d*$_6$, 300K, 500 MHz). 40
Figure S10. HMBC NMR spectrum of barceloneic acid A (**7**) (DMSO-*d*$_6$, 300K, 500 MHz). 40
Figure S11. ESI-HRMS spectrum of barceloneic acid A (**7**) under the positive mode. 41
Figure S12. ^{1}H NMR spectrum of banegasine (**10**) (DMSO-*d*$_6$, 300K, 600 MHz). 41
Figure S13. ESI-HRMS spectrum of banegasine (**10**) under the positive mode. 42
Figure S14. ^{1}H NMR spectrum of *cyclo*(*N*Me-L-3,5-dichlorotyrosin-Dhb) (**11**) (600 MHz, DMSO-*d*$_6$, 300 K). 42
Figure S15. ^{13}C NMR spectrum of *cyclo*(*N*Me-L-3,5-dichlorotyrosin-Dhb) (**11**) (150 MHz, DMSO-*d*$_6$, 300 K). 43
Figure S16. DEPT135 spectrum of *cyclo*(*N*Me-L-3,5-dichlorotyrosin-Dhb) (**11**) (600 MHz, DMSO-*d*$_6$, 300 K). 43
Figure S17. COSY spectrum of *cyclo*(*N*Me-L-3,5-dichlorotyrosin-Dhb) (**11**) (600 MHz, DMSO-*d*$_6$, 300 K). 44
Figure S18. HSQC spectrum of *cyclo*(*N*Me-L-3,5-dichlorotyrosin-Dhb) (**11**) (600 MHz, DMSO-*d*$_6$, 300 K). 44
Figure S19. HMBC spectrum of *cyclo*(*N*Me-L-3,5-dichlorotyrosin-Dhb) (**11**) (600 MHz, DMSO-*d*$_6$, 300 K). 45
Figure S20. NOESY spectrum of *cyclo*(*N*Me-L-3,5-dichlorotyrosin-Dhb) (**11**) (600 MHz, DMSO-*d*$_6$, 300 K). 45
Figure S21. ESI-HRMS spectrum of *cyclo*(*N*Me-L-3,5-dichlorotyrosin-Dhb) (**11**). 46
Figure S22. ESI-HRMS^2 spectrum of *cyclo*(*N*Me-L-3,5-dichlorotyrosin-Dhb) (**11**). 46
Figure S23. ESI-HRMS spectrum of rubrominin A (**12**). 47
Figure S24. ESI-HRMS spectrum of rubrominin B (**13**). 48
Figure S25. ESI-HRMS spectrum of rubrominin A (**12**) labled with L-Serine-2,3,3-d3. 48
Figure S26. ESI-HRMS spectrum of rubrominin B (**13**) labled with L-serine-2,3,3-D3. 49

FIGURE S27. ESI-HRMS SPECTRUM OF RUBROMININ A (**12**) LABLED WITH DL-CYSTEIN-3,3-D2. 49
FIGURE S28. ESI-HRMS SPECTRUM OF RUBROMININ B (**13**) LABLED WITH DL-CYSTEIN-3,3-D2. 50
FIGURE S29. UHPLC-MS CHROMATOGRAM OF MARFEY'S REACTION (1): A) EXTRACTED ION CHROMATOGRAMS OF MARFEY'S DERIVATIVE OF STANDARD L-ALANINE (M/Z 342.00, TR = 3.83 MIN), D-ALANINE (M/Z 342.00, TR = 4.17 MIN); B) EXTRACTED ION CHROMATOGRAMS OF MARFEY'S DERIVATIVE OF STANDARD GLYCINE (M/Z 328.00, TR = 3.60 MIN); C) EXTRACTED ION CHROMATOGRAMS OF MARFEY'S DERIVATIVE OF STANDARD L-PHENYLALANINE (M/Z 418.00, TR = 4.73 MIN), D-PHENYLALANINE (M/Z 418.00, TR = 4.93 MIN); D) EXTRACTED ION CHROMATOGRAMS OF MARFEY'S DERIVATIVE OF STANDARD L-SERINE (M/Z 358.00, TR = 2.94 MIN), D-SERINE (M/Z 358.00, TR = 3.15 MIN). 51
FIGURE S30. UHPLC-MS CHROMATOGRAM OF MARFEY'S REACTION (2): E) EXTRACTED ION CHROMATOGRAMS OF MARFEY'S DERIVATIVE OF STANDARD L-THEORINE (M/Z 372.00, TR = 3.26 MIN), D-THEORINE (M/Z 372.00, TR = 3.87 MIN); F) EXTRACTED ION CHROMATOGRAMS OF MARFEY'S DERIVATIVE OF STANDARD L-PROLINE (M/Z 368.00, TR = 3.98 MIN), D-PROLINE (M/Z 368.00, TR = 4.12 MIN); G) EXTRACTED ION CHROMATOGRAMS OF PROPOSED MARFEY'S DERIVATIVE OF LANTHIONINE (M/Z 461.0); H) EXTRACTED ION CHROMATOGRAMS OF PROPOSED MARFEY'S DERIVATIVE OF METHYLLANTHIONINE (M/Z 475.0) . 52

Table S1. Information of colonies of *Macrotermes natalensis* (Mn) used for isolation of actinobacteria for metatranscriptome data (including one *Odontotermes* colony [Od]), along with their geographic locations and year of collection.

colony ID	geographic location	GPS location	year	use
Mn101	ARC	S25 43 41.9 E28 14 06.1	2010	Bacteria isolation
Mn102	ARC	S25 43 45.2 E28 14 05.8	2010	Bacteria isolation
Mn103	ARC	S25 43 45.9 E28 14 08.9	2010	Bacteria isolation
Mn104	ARC	S25 43 44.8 E28 14 20.6	2010	Bacteria isolation
Mn105	Mookgophong	S24 40 30.5 E28 47 50.4	2010	Bacteria isolation
Mn106	Mookgophong	S24 40 30.5 E28 47 50.4	2010	Bacteria isolation
Mn107	Mookgophong	S24 40 30.5 E28 47 50.4	2010	Bacteria isolation
Mn110	ARC	S25 43 53.2 E28 14 12.0	2010	Bacteria isolation
Mn156	Experimental farm	S25 44.623 E28 15.655	2016	metatranscriptomic
Mn160	Experimental farm	S25 44 34.7 E28 15 38.7	2015	Bacteria isolation
Mn162	Mookgophong	S24 40 30.5 E28 47 50.4	2015	Bacteria isolation
Mn163	ARC	S25 43.761 E28 14.167	2015	Bacteria isolation
Od127	Experimental farm	S25 44.562 E28 15.391	2016	metatranscriptomic

Table S2. Strains isolated from fungus-growing termites, including the medium they were initially isolated on, their ID and the ID of the chemical extract.

Colony	Termite species	Colony part	Bacteria genus	Isolation media	Bacteria ID	Extract ID
Mn101	*Macrotermes natalensis*	abdomen	*Streptomyces*	chitin	RB90	E77
Mn101	*Macrotermes natalensis*	abdomen	*Streptomyces*	chitin	RB91	E49
Mn101	*Macrotermes natalensis*	abdomen	*Streptomyces*	chitin	RB92	E144
Mn101	*Macrotermes natalensis*	abdomen	*Streptomyces*	chitin	RB93	E148
Mn101	*Macrotermes natalensis*	abdomen	*Streptomyces*	MC	RB94	E61
Mn102	*Macrotermes natalensis*	comb	*Streptomyces*	chitin	RB102	E143
Mn103	*Macrotermes natalensis*	comb	*Streptomyces*	MC	RB3	E47
Mn103	*Macrotermes natalensis*	comb	*Streptomyces*	chitin	RB108	E45
Mn103	*Macrotermes natalensis*	comb	*Streptomyces*	MC	RB111	E154
Mn104	*Macrotermes natalensis*	abdomen	*Streptomyces*	MC	RB2	E58
Mn104	*Macrotermes natalensis*	abdomen	*Streptomyces*	chitin	RB95	E149
Mn104	*Macrotermes natalensis*	abdomen	*Streptomyces*	chitin	RB96	E150
Mn104	*Macrotermes natalensis*	abdomen	*Streptomyces*	chitin	RB97	E151
Mn104	*Macrotermes natalensis*	comb	*Streptomyces*	chitin	RB103	E153
Mn105	*Macrotermes natalensis*	abdomen	*Streptomyces*	MC	RB98	E57
Mn105	*Macrotermes natalensis*	abdomen	*Actinomadura*	chitin	RB99	E60
Mn105	*Macrotermes natalensis*	abdomen	*Streptomyces*	chitin	RB100	E48
Mn105	*Macrotermes natalensis*	abdomen	*Streptomyces*	chitin	RB101	E152
Mn105	*Macrotermes natalensis*	comb	*Streptomyces*	chitin	RB104	E59
Mn105	*Macrotermes natalensis*	comb	*Streptomyces*	chitin	RB109	E147
Mn106	*Macrotermes natalensis*	comb	*Streptomyces*	chitin	RB105	E145
Mn106	*Macrotermes natalensis*	comb	*Streptomyces*	chitin	RB110	RB142
Mn106	*Macrotermes natalensis*	abdomen	*Arthrobacter*	chitin	RB112	E155
Mn107	*Macrotermes natalensis*	comb	*Streptomyces*	chitin	RB106	E146
Mn110	*Macrotermes natalensis*	comb	*Streptomyces*	chitin	RB107	E46
Mn160	*Macrotermes natalensis*	gut	*Streptomyces*	chitin	RB4	E74
Mn160	*Macrotermes natalensis*	gut	*Streptomyces*	chitin	RB5	E1
Mn160	*Macrotermes natalensis*	gut	*Micromonospora*	chitin	RB6	E4
Mn160	*Macrotermes natalensis*	gut	*Streptomyces*	chitin	RB7	E3
Mn160	*Macrotermes natalensis*	gut	*Micromonospora*	chitin	RB8	E5
Mn160	*Macrotermes natalensis*	gut	*Streptomyces*	chitin	RB9	E103
Mn160	*Macrotermes natalensis*	gut	*Streptomyces*	chitin	RB10	E8
Mn160	*Macrotermes natalensis*	gut	*Streptomyces*	chitin	RB11	E9
Mn160	*Macrotermes natalensis*	gut	*Streptomyces*	chitin	RB13	E11
Mn160	*Macrotermes natalensis*	gut	*Streptomyces*	chitin	RB14	E12/E13
Mn160	*Macrotermes natalensis*	gut	*Streptomyces*	chitin	RB15	E65
Mn160	*Macrotermes natalensis*	gut	*Streptomyces*	chitin	RB16	E66
Mn160	*Macrotermes natalensis*	gut	*Streptomyces*	chitin	RB17	E38
Mn160	*Macrotermes natalensis*	gut	*Cellulosimicrobium*	chitin	RB18	E68
Mn160	*Macrotermes natalensis*	gut	*Nocardia*	chitin	RB20	E16
Mn160	*Macrotermes natalensis*	gut	*Nocardia*	chitin	RB21	E17

Mn160	*Macrotermes natalensis*	gut	*Luteimicrobium*	chitin	RB22	E18/E19
Mn160	*Macrotermes natalensis*	gut	*Micromonospora*	chitin	RB23	E50
Mn160	*Macrotermes natalensis*	gut	*Mycobacterium*	chitin	RB24	E20/E21
Mn160	*Macrotermes natalensis*	gut	*Streptomyces*	chitin	RB25	E135
Mn160	*Macrotermes natalensis*	gut	*Streptomyces*	chitin	RB26	E22/E23
Mn160	*Macrotermes natalensis*	gut	*Streptomyces*	chitin	RB27	E26/E27
Mn160	*Macrotermes natalensis*	gut	*Streptomyces*	chitin	RB28	E67
Mn160	*Macrotermes natalensis*	gut	*Actinomadura*	chitin	RB29	E62
Mn160	*Macrotermes natalensis*	gut	*Streptomyces*	chitin	RB30	E40
Mn160	*Macrotermes natalensis*	gut	*Streptomyces*	chitin	RB31	E42
Mn160	*Macrotermes natalensis*	gut	*Mycobacterium*	chitin	RB32	E43
Mn160	*Macrotermes natalensis*	gut	*Mycobacterium*	chitin	RB33	E44
Mn160	*Macrotermes natalensis*	gut	*Streptomyces*	chitin	RB34	E80
Mn160	*Macrotermes natalensis*	gut	*Streptomyces*	chitin	RB36	E63
Mn160	*Macrotermes natalensis*	gut	*Streptomyces*	chitin	RB38	E54
Mn160	*Macrotermes natalensis*	gut	*Streptomyces*	chitin	RB40	E56
Mn160	*Macrotermes natalensis*	gut	*Sphaerisporangium*	chitin	RB41	E113
Mn160	*Macrotermes natalensis*	gut	*Streptomyces*	chitin	RB42	E70
Mn160	*Macrotermes natalensis*	gut	*Streptomyces*	chitin	RB44	E75
Mn160	*Macrotermes natalensis*	gut	*Streptomyces*	chitin	RB45	E86
Mn160	*Macrotermes natalensis*	gut	*Streptomyces*	chitin	RB47	E136
Mn160	*Macrotermes natalensis*	gut	*Streptomyces*	chitin	RB48	E76
Mn160	*Macrotermes natalensis*	gut	*Streptomyces*	chitin	RB50	E88
Mn160	*Macrotermes natalensis*	gut	*Streptomyces*	chitin	RB52	E91
Mn160	*Macrotermes natalensis*	gut	*Streptomyces*	chitin	RB53	E93
Mn160	*Macrotermes natalensis*	gut	*Streptomyces*	chitin	RB54	E94
Mn160	*Macrotermes natalensis*	gut	*Streptomyces*	chitin	RB55	E95
Mn160	*Macrotermes natalensis*	gut	*Nocardia*	chitin	RB56	E104
Mn160	*Macrotermes natalensis*	gut	*Streptomyces*	chitin	RB60	E105
Mn160	*Macrotermes natalensis*	gut	*Streptomyces*	chitin	RB62	E109
Mn160	*Macrotermes natalensis*	gut	*Streptomyces*	chitin	RB63	E96
Mn160	*Macrotermes natalensis*	gut	*Microbispora*	chitin	RB64	E97
Mn160	*Macrotermes natalensis*	gut	*Actinomadura*	chitin	RB66	E98
Mn160	*Macrotermes natalensis*	gut	*Actinomadura*	chitin	RB68	E99
Mn160	*Macrotermes natalensis*	gut	*Streptomyces*	chitin	RB72	E100
Mn160	*Macrotermes natalensis*	gut	*Streptomyces*	MC	RB78	E119
Mn160	*Macrotermes natalensis*	gut	*Streptomyces*	MC	RB79	E120
Mn160	*Macrotermes natalensis*	gut	*Streptomyces*	MC	RB80	E121
Mn160	*Macrotermes natalensis*	gut	*Actinomadura*	MC	RB81	E122
Mn160	*Macrotermes natalensis*	gut	*Curtobacterium*	chitin	RB129	E41
Mn162	*Macrotermes natalensis*	gut	*Leifsonia*	ISP2	RB71	E110
Mn162	*Macrotermes natalensis*	gut	*Streptomyces*	MC	RB74	E102
Mn162	*Macrotermes natalensis*	gut	*Streptomyces*	chitin	RB76	E112/E113
Mn162	*Macrotermes natalensis*	gut	*Aeromicrobium*	chitin	RB77	E118
Mn162	*Macrotermes natalensis*	gut	*Streptomyces*	chitin	RB82	E123
Mn162	*Macrotermes natalensis*	gut	*Streptomyces*	chitin	RB83	E124

Mn162	*Macrotermes natalensis*	gut	*Leifsonia*	chitin	RB84	E125
Mn162	*Macrotermes natalensis*	gut	*Streptomyces*	chitin	RB85	E126
Mn162	*Macrotermes natalensis*	gut	*Streptomyces*	chitin	RB86	E127
Mn162	*Macrotermes natalensis*	gut	*Streptomyces*	chitin	RB87	E128
Mn162	*Macrotermes natalensis*	gut	*Streptomyces*	chitin	RB88	E129
Mn162	*Macrotermes natalensis*	gut	*Leifsonia*	chitin	RB89	E130
Mn163	*Macrotermes natalensis*	abdomen	*Streptomyces*	chitin	RB113	E35
Mn163	*Macrotermes natalensis*	abdomen	*Streptomyces*	chitin	RB114	E78
Mn163	*Macrotermes natalensis*	comb	*Streptomyces*	chitin	RB115	E36
Mn163	*Macrotermes natalensis*	comb	*Streptomyces*	chitin	RB116	E37

Table S3. Identities of isolated actinobacterial strains, including the top three hits resulting from BLASTn searches against the NCBI database (https://blast.ncbi.nlm.nih.gov/Blast.cgi, last visit 26.07.2018, 00:58 AM).

Strain ID	Accession number	length [nt]	Hit	Max Score	Similarity[a]	Accession Number
**RB2**	KX344916	1374	*Streptomyces mirabilis* NBRC 13450<br>*Streptomyces mirabilis* CSSP107<br>*Streptomyces mirabilis* ATCC 27447	2519<br>2518<br>2495	100%<br>100%<br>99.93%	NR_041137.1<br>NR_115357.1<br>NR_114494.1
**RB3**	KX344917	1363	*Streptomyces puniciscabiei* S77<br>*Streptomyces durhamensis* NBRC 13441<br>*Streptomyces durhamensis* CSSP538	2462<br>2451<br>2451	99.56%<br>99.34%<br>99.34%	NR_025156.1<br>NR_112399.1<br>NR_043352.1
**RB4**	KX344918	1365	*Streptomyces yanglinensis* 1307<br>*Streptomyces paucisporeus* 1413<br>*Streptomyces polygonati* NEAU-G9	2427<br>2383<br>2355	99.04%<br>99.19%<br>98.02%	NR_043244.1<br>NR_043247.1<br>NR_148782.1
**RB5**	KY558696	1374	*Streptomyces aureoverticillatus* NBRC 12742<br>*Streptomyces aureoverticillatus* CSSP526<br>*Streptomyces variegatus* NBRC 15462	2390<br>2390<br>2381	98.54%<br>98.54%<br>98.32%	NR_112580.1<br>NR_043346.1<br>NR_112473.1
**RB6**	KY558697	1351	*Micromonospora tulbaghiae* TVU1<br>*Micromonospora echinospora* ATCC<br>*Micromonospora chalcea* ATCC 12452	2490<br>2447<br>2431	100%<br>99.63%<br>99.26%	NR_116241.1<br>NR_118843.1<br>NR_118842.1
**RB7**	KY558698	1368	*Streptomyces cacaoi* NBRC 12748<br>*Streptomyces violaceoruber* CSSP679<br>*Streptomyces smyrnaeus* SM3501	2505<br>2473<br>2381	99. 85%<br>99.63%<br>98.68%	NR_041061.1<br>NR_115407.1<br>NR_134201.1
**RB8**	KY312017	1362	*Micromonospora yasonensis* DS3186<br>*Micromonospora olivasterospora* MK-70<br>*Micromonospora viridifaciens* DSM 43909	2459<br>2420<br>2410	99.34%<br>98.825%<br>98.751%	NR_149817.1<br>NR_036966.1<br>NR_044889.1
**RB9**	MH044507	863	*Streptomyces hoynatensis* S1412<br>*Streptomyces specialis* GW41-1564<br>*Streptomyces hainanensis* YIM 47672	1500<br>1474<br>1474	98.49%<br>97.91%<br>97.56.%	NR_133871.1<br>NR_042689.1<br>NR_042561.1
**RB10**	KY558699	1367	*Streptomyces yanglinensis* 1307<br>*Streptomyces paucisporeus* 1413<br>*Streptomyces polygonati* NEAU-G9	2433<br>2394<br>2361	99.05<br>99.26%<br>98.09%	NR_043244.1<br>NR_043247.1<br>NR_148782.1

**RB11**	KY558700	1370	*Streptomyces yanglinensis* 1307<br>*Streptomyces paucisporeus* 1413<br>*Streptomyces polygonati* NEAU-G9	2425<br>2381<br>2359	98.90%<br>99.04%<br>98.02%	NR_043244.1<br>NR_043247.1<br>NR_148782.1
**RB13**	KY558735	1366	*Streptomyces violaceolatus* NBRC 13101<br>*Streptomyces humiferus* NBRC 12244<br>*Streptomyces violaceolatus* DSM 40438	2507<br>2507<br>2507	99.85%<br>99.85%<br>99.85%	NR_112370.1<br>NR_112243.1<br>NR_027223.1
**RB14**	KY558701	1361	*Streptomyces flavidovirens* NBRC 13039<br>*Streptomyces longisporus* NBRC 12885<br>*Streptomyces longisporus* ISP 5166	2420<br>2420<br>2420	99.34%<br>99.34%<br>99.34%	NR_041099.1<br>NR_112319.1<br>NR_025492.1
**RB15**	KY558702	1366	*Streptomyces rameus* NBRC 3782<br>*Streptomyces coacervatus* IFM 11055<br>*Streptomyces galbus* DSM 40089	2468<br>2440<br>2427	99.27%<br>98.90%<br>99.12%	NR_112497.1<br>NR_112916.1<br>NR_026178.1
**RB16**	KY558703	1359	*Streptomyces rameus* NBRC 3782<br>*Streptomyces coacervatus* IFM 11055<br>*Streptomyces galbus* DSM 40089	2449<br>2416<br>2409	99.19%<br>98.75%<br>99.04%	NR_112497.1<br>NR_112916.1<br>NR_026178.1
**RB17**	KY558704	1373	*Streptomyces lanatus* NBRC 12787<br>*Streptomyces echinatus* NBRC 12763<br>*Streptomyces lanatus* ISP 5090	2409<br>2409<br>2409	99.12%<br>99.12%<br>99.12%	NR_041220.1<br>NR_112264.1<br>NR_114826.1
**RB18**	KY558705	1371	*Cellulosimicrobium funkei* W6122<br>*Cellulosimicrobium aquatile* 3bp<br>*Cellulosimicrobium cellulans* DSM 43879	2507<br>2497<br>2488	99.78%<br>99.78%<br>99.49%	NR_042937.1<br>NR_146008.1<br>NR_119095.1
**RB20**	KY558706	1352	*Nocardia miyunensis*<br>*Nocardia miyunensis* 117<br>*Nocardia niigatensis* W8186	2318<br>2318<br>2313	98.44%<br>98.44%<br>97.63%	NR_117333.1<br>NR_043059.1<br>NR_117402.1
**RB21**	KY558707	1362	*Nocardia miyunensis*<br>*Nocardia miyunensis* 117<br>*Nocardia niigatensis* W8186	2394<br>2394<br>2388	98.82%<br>98.82%<br>98.31%	NR_117333.1<br>NR_043059.1<br>NR_117402.1
**RB22**	KY558708	1369	*Luteimicrobium album* RI148-Li105<br>*Luteimicrobium xylanilyticum* W-15<br>*Luteimicrobium subarcticum* R19-04	2460<br>2385<br>2333	99.41%<br>98.54%<br>97.88%	NR_108122.1<br>NR_126237.1<br>NR_112891.1
**RB23**	KY558709	1362	*Micromonospora chokoriensis* 2-19<br>*Micromonospora violae* NEAU-P5<br>*Micromonospora saelicesensis* Lupac 09	2508<br>2492<br>2475	100%<br>99.85%<br>99.71%	NR_041349.1<br>NR_134089.1<br>NR_042312.1

**RB24**	KY312018	1369	*Mycobacterium parafortuitum* DSM 43528	2459	99.27%	NR_026285.1
			Mycobacterium psychrotolerans WA101	2438	99.12%	NR_028998.1
			Mycobacterium sphagni Sph 38	2436	99.27%	NR_104774.1
**RB25**	KY558710	1364	*Streptomyces rameus* NBRC 3782	2470	99.41 %	NR_112497.1
			Streptomyces coacervatus IFM 11055	2436	98.97%	NR_112916.1
			Streptomyces galbus DSM 40089	2431	99.27%	NR_026178.1
**RB26**	KY558711	1374	*Streptomyces humidus* NBRC 12877	2447	98.83%	NR_112316.1
			Streptomyces resistomycificus NBRC 12814	2442	98.91%	NR_112287.1
			Streptomyces caniferus NRRL B-16358	2440	98.83%	NR_116095.1
**RB27**	KY558712	1375	*Streptomyces lanatus* NBRC 12787	2420	99.12%	NR_041220.1
			Streptomyces shenzhenensis 172115	2418	98.69%	NR_118018.1
			Streptomyces lanatus ISP 5090	2418	99.12%	NR_114826.1
**RB28**	KY558713	1357	*Streptomyces durhamensis* NBRC 13441	2431	99.264%	NR_112399.1
			Streptomyces durhamensis CSSP538	2431	99.26%	NR_043352.1
			Streptomyces yaanensis Z4	2414	98.89%	NR_132305.1
**RB29**	KY312019	1355	*Parvopolyspora pallida* IFO 14788	2409	99.63%	NR_112013.1
			Actinomadura rayongensis RY35-68	2379	99.19%	NR_134688.1
			Actinomadura atramentaria DSM 43919	2368	98.52%	NR_114850.1
**RB30**	KY558714	1375	*Streptomyces regensis* NBRC 13448	2519	100%	NR_112402.1
			Streptomyces regensis NRRL B-11479	2514	99.93%	NR_043495.1
			Streptomyces rameus NBRC 3782	2447	99.05%	NR_112497.1
**RB31**	KY558715	1369	*Streptomyces chattanoogensis* NBRC 12754	2468	99.20%	NR_112260.1
			Streptomyces sioyaensis NRRL B-5408	2462	99.12%	NR_043498.1
			Streptomyces sioyaensis NBRC 12820	2462	99.12%	NR_112289.1
**RB32**	KY558716	1352	*Mycobacterium obuense* 47001	2433	99.48%	NR_029218.1
			Mycobacterium chubuense ATCC 27278	2425	99.04%	NR_041902.1
			Mycobacterium houstonense ATCC 49403	2423	99.33%	NR_042913.1
**RB33**	KY558717	1357	*Mycobacterium madagascariense* P2	2370	98.60%	NR_104690.1
			Mycobacterium barrassiae CIP 108545	2361	98.60%	NR_115330.1
			Mycobacterium smegmatis ATCC 19420	2355	98.67%	NR_115233.1
**RB34**	KY312020	1367	*Streptomyces echinatus* NBRC 12763	2484	99.56%	NR_112264.1
			Streptomyces echinatus ISP 5013	2475	99.49%	NR_114823.1
			Streptomyces durhamensis NBRC 13441	2418	99.56%	NR_112399.1

**RB36**	KY558718	1349	*Streptomyces coeruleorubidus* ISP 5145	2322	98.64%	NR_114791.1
			Streptomyces bellus NBRC 12844	2320	98.14%	NR_041222.1
			Streptomyces coerulescens CSSP046	2320	98.35%	NR_043337.1
**RB38**	KY558719	1365	*Streptomyces misionensis* NBRC 13063	2503	99.85%	NR_112355.1
			Streptomyces misionensis JCM 4497	2499	99.85%	NR_044138.1
			Streptomyces phaeoluteichromatogenes NRRL B-5799	2486	99.56%	NR_042096.1
**RB40**	KY558720	1365	*Streptomyces thermocarboxydus* NBRC 16323	2516	100%	NR_112585.1
			Streptomyces thermocarboxydus AT37	2510	99.93%	NR_026072.1
			Streptomyces lusitanus NBRC 13464	2486	99.63%	NR_041143.1
**RB41**	KY558721	1341	*Sphaerisporangium corydalis* NEAU-YHS15	2302	98.05	NR_148314.1
			Sphaerisporangium cinnabarinum DSM 44094	2290	98.05%	NR_044874.1
			Sphaerisporangium rufum R10-82	2261	98.05%	NR_125470.1
**RB42**	KY312021	1355	*Streptomyces phaeochromogenes* NRRL B-1248	2492	99.85%	NR_116382.1
			Streptomyces phaeochromogenes ATCC 3338	2492	99.85%	NR_116381.1
			Streptomyces phaeochromogenes NBRC 3180	2492	99.85%	NR_041200.1
**RB44**	KY558722	1364	*Streptomyces misionensis* NBRC 13063	2512	100%	NR_112355.1
			Streptomyces misionensis JCM 4497	2508	100%	NR_044138.1
			Streptomyces phaeoluteichromatogenes NRRL B-5799	2495	99.78%	NR_042096.1
**RB45**	KY558723	1371	*Streptomyces misionensis* NBRC 13063	2532	100%	NR_112355.1
			Streptomyces misionensis JCM 4497	2527	100%	NR_044138.1
			Streptomyces phaeoluteichromatogenes NRRL B-5799	2516	99.78%	NR_042096.1
**RB47**	KY558724	1371	*Streptomyces atriruber* NRRL B-24165	2427	99.12%	NR_116447.1
			Streptomyces chartreusis ISP 5085	2405	98.83%	NR_114825.1
			Streptomyces bobili NBRC 16166	2405	98.69%	NR_112584.1
**RB48**	KY558725	1365	*Streptomyces costaricanus* NBRC 100773	2514	100%	NR_041414.1
			Streptomyces murinus NBRC 14802	2514	100%	NR_112445.1
			Streptomyces murinus NBRC 12799	2514	100%	NR_041072.1
**RB50**	KY558726	1372	*Streptomyces thermocarboxydus* NBRC 16323	2534	100%	NR_112585.1
			Streptomyces thermocarboxydus AT37	2529	99.93%	NR_026072.1
			Streptomyces lusitanus NBRC 13464	2501	99.56%	NR_041143.1
**RB52**	KY558727	1372	*Streptomyces shenzhenensis* 172115	2499	99.64%	NR_118018.1
			Streptomyces graminisoli JR-19	2494	99.56%	NR_125577.1
			Streptomyces jiujiangensis JXJ 0074	2466	99.20%	NR_125706.1

**RB53**	KY558728	1365	*Streptomyces yanglinensis* 1307<br>*Streptomyces paucisporeus* 1413<br>*Streptomyces polygonati* NEAU-G9	2442<br>2398<br>2375	99.05%<br>99.19%<br>98.17%	NR_043244.1<br>NR_043247.1<br>NR_148782.1
**RB54**	MH044508	953	*Streptomyces paucisporeus* 1413<br>*Streptomyces yanglinensis* 1307<br>*Streptomyces yogyakartensis* DSM 41766	1709<br>1692<br>1688	99.26%<br>98.95%<br>98.74%	NR_043247.1<br>NR_043244.1<br>NR_117958.1
**RB55**	KY558729	1369	*Streptomyces shenzhenensis* 172115<br>*Streptomyces graminisoli* JR-19<br>*Streptomyces jiujiangensis* JXJ 0074	2501<br>2495<br>2468	99.63%<br>99.56%<br>99.20%	NR_118018.1<br>NR_125577.1<br>NR_125706.1
**RB56**	KY558730	1362	*Nocardia pseudobrasiliensis* ATCC 51512<br>*Nocardia pseudobrasiliensis* DSM 44290<br>*Nocardia rayongensis* RY45-3	2368<br>2368<br>2362	98.16%<br>98.16%<br>98.05%	NR_117336.1<br>NR_041864.1<br>NR_149226.1
**RB60**	KY558731	1368	*Streptomyces purpurascens* NBRC 13077<br>*Streptomyces purpurascens* JCM 4509<br>*Streptomyces spinoverrucosus* NBRC 14228	2464<br>2464<br>2451	99.41%<br>99.41%<br>99.27%	NR_112521.1<br>NR_104281.1<br>NR_041159.1
**RB62**	KY312022	1358	*Streptomyces rubrogriseus* NBRC 15455<br>*Streptomyces rubrogriseus* DSM 41477<br>*Streptomyces albogriseolus* DSM 40003	2479<br>2473<br>2473	99.71%<br>99.63%<br>99.63%	NR_041188.1<br>NR_114667.1<br>NR_042760.1
**RB63**	KY558732	1365	*Streptomyces rubrogriseus* NBRC 15455<br>*Streptomyces rubrogriseus* DSM 41477<br>*Streptomyces albogriseolus* DSM 40003	2512<br>2507<br>2507	100%<br>99.93%<br>99.93%	NR_041188.1<br>NR_114667.1<br>NR_042760.1
**RB64**	KY558733	1369	*Microbispora corallina* DF-32<br>*Microbispora hainanensis* 211020<br>*Microbispora bryophytorum* NEAU-TX2-2	2494<br>2442<br>2377	99.71%<br>99.71%<br>99.04%	NR_028605.1<br>NR_116600.1<br>NR_134769.1
**RB66**	MH044509	1366	*Actinomadura nitritigenes* NBRC 15918<br>*Actinomadura nitritigenes* L 46<br>*Actinomadura montaniterrae*	2507<br>2495<br>2449	99.93%<br>99.78%<br>99.71%	NR_112736.1<br>NR_025652.1<br>NR_151939.1
**RB68**	MH044510	1356	*Actinomadura hibisca* DSM 44148<br>*Actinomadura nitritigenes* NBRC 15918<br>*Actinomadura hibisca* IMSNU 22185	2338<br>2327<br>2326	98.30%<br>97.79%<br>97.08%	NR_114849.1<br>NR_112736.1<br>NR_042031.1
**RB71**	KY558734	1374	*Leifsonia shinshuensis* DB 102<br>*Leifsonia xyli* subsp. *cynodontis* JCM 9733<br>*Leifsonia lichenia* 2Sb	2468<br>2440<br>2418	99.27%<br>99.34%<br>98.61%	NR_043663.1<br>NR_125442.1<br>NR_112644.1

**RB72**	MH044511	1366	*Streptomyces alni* D65	2453	99.34%	NR_043866.1
			Streptomyces abietis A191	2392	98.39%	NR_114347.1
			Streptomyces cocklensis BK168	2364	98.02%	NR_108501.1
**RB74**	MH044512	975	*Streptomyces flaveolus* NRRL B-1334	1751	99.28%	NR_116094.1
			Streptomyces glaucescens NRRL B-2706	1751	99.28%	NR_115773.1
			Streptomyces viridochromogenes NBRC 13347	1751	99.28%	NR_112526.1
**RB76**	KY558736	1376	*Streptomyces griseoaurantiacus* NBRC 15440	2510	99.78%	NR_041186.1
			Streptomyces griseoaurantiacus DSM 40430	2510	99.78%	NR_115231.1
			Streptomyces jietaisiensis FXJ46	2479	99.56%	NR_042836.1
**RB77**	KY558737	1363	*Aeromicrobium massiliense* JC14	2495	99.71%	NR_125588.1
			Aeromicrobium erythreum NRRL B-3381	2254	96.99%	NR_024846.1
			Aeromicrobium alkaliterrae KSL-107	2248	96.70%	NR_043207.1
**RB78**	KY558738	1365	*Streptomyces rameus* NBRC 3782	2460	99.49%	NR_112497.1
			Streptomyces coacervatus IFM 11055	2433	98.67%	NR_112916.1
			Streptomyces galbus DSM 40089	2420	99.34%	NR_026178.1
**RB79**	KY558739	1364	*Streptomyces cocklensis* BK168	2422	99.34%	NR_108501.1
			Streptomyces bryophytorum NEAU-HZ10	2425	99.12%	NR_146707.1
			Streptomyces yanglinensis 1307	2401	98.53%	NR_043244.1
**RB80**	MH044513	1365	*Streptomyces cocklensis* BK168	2444	99.34%	NR_108501.1
			Streptomyces bryophytorum NEAU-HZ10	2427	99.12%	NR_146707.1
			Streptomyces yanglinensis 1307	2403	98.53%	NR_043244.1
**RB81**	MH044514	1356	*Actinomadura nitritigenes* NBRC 15918	2488	99.85%	NR_112736.1
			Actinomadura nitritigenes L 46	2477	99.70%	NR_025652.1
			Actinomadura montaniterrae	2449	99.63%	NR_151939.1
**RB82**	KY558740	1376	*Streptomyces gilvifuscus* T113	2447	99.49%	NR_137389.1
			Streptomyces sasae JR-39	2440	98.98%	NR_137361.1
			Streptomyces panaciradicis 1MR-8	2425	98.76%	NR_134200.1
**RB83**	KY558742	1370	*Streptomyces actinomycinicus* RCU-197	2438	98.98%	NR_147753.1
			Streptomyces sasae JR-39	2436	99.27%	NR_137361.1
			Streptomyces neopeptinius KNF 2047	2427	99.20%	NR_116261.1
**RB84**	KY558743	1365	*Leifsonia xyli* subsp. *cynodontis* JCM 9733	2494	99.71%	NR_125442.1
			Leifsonia shinshuensis DB 102	2494	99.19%	NR_043663.1
			Leifsonia naganoensis DB103	2455	99.19%	NR_043662.1

**RB85**	MH044515	962	*Streptomyces nogalater* NBRC 13445	1744	99.48%	NR_112401.1
			Streptomyces lavenduligriseus NBRC 13405	1744	99.48%	NR_112391.1
			Streptomyces nogalater JCM 4799	1744	99.48%	NR_112122.1
**RB86**	KY558741	1360	*Streptomyces filipinensis* NBRC 12860	2453	99.19%	NR_041083.1
			Streptomyces durhamensis NBRC 13441	2447	99.12%	NR_112399.1
			Streptomyces durhamensis CSSP538	2447	99.12%	NR_043352.1
**RB87**	KY558744	1366	*Streptomyces glaucescens* NRRL B-2706	2440	98.97%	NR_115773.1
			Streptomyces glaucescens NBRC 12774	2440	98.97%	NR_041219.1
			Streptomyces minutiscleroticus NBRC 13361	2407	98.53%	NR_112379.1
**RB88**	KY558745	1375	*Streptomyces sasae* JR-39	2501	99.71%	NR_137361.1
			Streptomyces panaciradicis 1MR-8	2497	99.64%	NR_134200.1
			Streptomyces capoamus NBRC 13411	2460	99.20%	NR_112394.1
**RB89**	KY558746	1365	*Leifsonia xyli* subsp. *cynodontis* JCM 9733	2492	99.71%	NR_125442.1
			Leifsonia shinshuensis DB 102	2453	99.19%	NR_043663.1
			Leifsonia naganoensis DB103	2442	99.19%	NR_043662.1
**RB90**	KY558671	1365	*Streptomyces californicus* NBRC 12750	2514	100%	NR_112257.1
			Streptomyces californicus NBRC 3386	2514	100%	NR_112486.1
			Streptomyces californicus CSSP711	2514	100%	NR_115432.1
**RB91**	KY558672	1359	*Streptomyces rameus* NBRC 3782	2416	98.75%	NR_112497.1
			Streptomyces coacervatus IFM 11055	2383	98.31%	NR_112916.1
			Streptomyces levis NRRL B-16370	2375	98.31%	NR_115778.1
**RB92**	KY558669	1366	*Streptomyces pratensis* ch24	2516	100%	NR_125616.1
			Streptomyces pratensis ch24	2516	100%	NR_125619.1
			Streptomyces anulatus NBRC 13369	2516	100%	NR_112527.1
**RB93**	KY558670	1364	*Streptomyces griseus* NBRC 15744	2510	100%	NR_112475.1
			Streptomyces erumpens NBRC 15403	2510	100%	NR_112455.1
			Streptomyces griseus NBRC 12875	2510	100%	NR_112314.1
**RB94**	KY558673	1361	*Streptomyces regensis* NBRC 13448	2481	99.56%	NR_112402.1
			Streptomyces regensis NRRL B-11479	2475	99.49%	NR_043495.1
			Streptomyces rameus NBRC 3782	2409	98.60%	NR_112497.1
**RB95**	KY558678	1377	*Streptomyces bungoensis* NBRC 15711	2436	99.12%	NR_041191.1
			Streptomyces galbus NBRC 12864	2422	99.05%	NR_026178.1
			Streptomyces longwoodensis NBRC 14251	2425	98.98%	NR_041161.1

**RB96**	KY558680	1370	*Streptomyces virginiae* NBRC 3729	2518	100%	NR_112496.1
			Streptomyces cinnamonensis NBRC 15873	2518	100%	NR_041194.1
			Streptomyces virginiae NBRC 12827	2518	100%	NR_041078.1
**RB97**	KY558679	1372	*Streptomyces humidus* NBRC 12877	2435	98.83%	NR_112316.1
			Streptomyces resistomycificus NBRC 12814	2429	98.91%	NR_112287.1
			Streptomyces caniferus NRRL B-16358	2427	98.83%	NR_116095.1
**RB98**	KY558683	1370	*Streptomyces achromogenes* subsp. *rubradiris* NBRC 14000	2473	99.42%	NR_112428.1
			Streptomyces achromogenes subsp. *rubradiris* CSSP713	2499	99.42%	NR_043365.1
			Streptomyces nodosus NBRC 12895	2455	99.27%	NR_112324.1
**RB99**	KY558684	1368	*Actinomadura nitritigenes* NBRC 15918	2512	99.93%	NR_112736.1
			Actinomadura nitritigenes L 46	2501	99.78%	NR_025652.1
			Actinomadura montaniterrae	2473	99.71%	NR_151939.1
**RB100**	KY558685	1376	*Streptomyces collinus* NBRC 12759	2420	98.83%	NR_041063.1
			Streptomyces collinus DSM 40129	2420	98.83%	NR_114792.1
			Streptomyces viridochromogenes NBRC 13347	2416	99.20%	NR_112526.1
**RB101**	KY558686	1373	*Streptomyces collinus* NBRC 12759	2423	98.91%	NR_041063.1
			Streptomyces collinus DSM 40129	2423	98.91%	NR_114792.1
			Streptomyces viridochromogenes NBRC 13347	2420	99.27%	NR_112526.1
**RB102**	KY558674	1375	*Streptomyces coelicolor* DSM 40233	2525	100%	NR_116633.1
			Streptomyces sampsonii NBRC 13083	2525	99.93%	NR_112362.1
			Streptomyces coelicolor NBRC 12854	2525	99.93%	NR_112305.1
**RB103**	KY558677	1367	*Streptomyces jiujiangensis* JXJ 0074	2457	99.27%	NR_125706.1
			Streptomyces graminisoli JR-19	2446	99.41%	NR_125577.1
			Streptomyces hyaluromycini MB-PO13	2429	98.90%	NR_146364.1
**RB104**	KY558682	1366	*Streptomyces graminisoli* JR-19	2460	99.27%	NR_125577.1
			Streptomyces actinomycinicus RCU-197	2435	99.56%	NR_147753.1
			Streptomyces shenzhenensis 172115	2433	98.90%	NR_118018.1
**RB105**	KY558689	1365	*Streptomyces pseudogriseolus* NRRL B	2510	100%	NR_043835.1
			Streptomyces pseudogriseolus NBRC 12902	2510	100%	NR_112329.1
			Streptomyces gancidicus NBRC 15412	2507	100%	NR_041179.1
**RB106**	KY558690	1360	*Streptomyces thermocarboxydovorans* AT52	2398	98.75%	NR_026071.1
			Streptomyces glomeratus NBRC 15898	2390	99.04%	NR_041409.1
			Streptomyces somaliensis CSSP138	2388	98.60%	NR_115358.1

**RB107**	KY558691	1373	*Streptomyces seoulensis* NBRC 16668<br>*Streptomyces recifensis* NBRC 12813<br>*Streptomyces griseoluteus* CSSP409	2512<br>2510<br>2501	99.75%<br>99.93%<br>99.71%	NR_041432.1<br>NR_041074.1<br>NR_043341.1
**RB108**	KY558675	1365	*Streptomyces pulveraceus* NBRC 3855<br>*Streptomyces atratus* NRRL B-16927<br>*Streptomyces gelaticus* NRRL B-2928	2501<br>2479<br>2479	99.85%<br>99.56%<br>99.56%	NR_041213.1<br>NR_043490.1<br>NR_043488.1
**RB109**	KY558681	1372	*Streptomyces cocklensis* BK168<br>*Streptomyces bryophytorum* NEAU-HZ10<br>*Streptomyces yanglinensis* 1307	2436<br>2420<br>2407	99.63%<br>99.12%<br>98.69%	NR_108501.1<br>NR_146707.1<br>NR_043244.1
**RB110**	KY558688	1374	*Streptomyces californicus* NBRC 12750<br>*Streptomyces californicus* NBRC 3386<br>*Streptomyces californicus* CSSP711	2523<br>2523<br>2523	100%<br>100%<br>100%	NR_112257.1<br>NR_112486.1<br>NR_115432.1
**RB111**	KY558676	1362	*Streptomyces costaricanus* NBRC 100773<br>*Streptomyces murinus* NBRC 14802<br>*Streptomyces murinus* NBRC 12799	2451<br>2451<br>2451	99.27%<br>99.27%<br>99.27%	NR_041414.1<br>NR_112445.1<br>NR_041072.1
**RB112**	KY558678	1368	*Arthrobacter luteolus* CF-25<br>*Arthrobacter koreensis* CA15-8<br>*Arthrobacter gandavensis* R 5812	2481<br>2466<br>2444	99.49%<br>99.34%<br>99.12%	NR_025362.1<br>NR_025665.1<br>NR_025475.1
**RB113**	KY558692	1371	*Streptomyces ramulosus* NRRL B-2714<br>*Streptomyces ramulosus* NBRC 15798<br>*Streptomyces ramulosus* NBRC 12812	2521<br>2521<br>2521	100%<br>100%<br>100%	NR_043503.1<br>NR_112476.1<br>NR_112286.1
**RB114**	KY558695	1374	*Streptomyces lannensis* TA4-8<br>*Streptomyces chiangmaiensis* TA4-1<br>*Streptomyces glomeratus* NBRC 15898	2521<br>2429<br>2390	100%<br>98.83%<br>98.40%	NR_113181.1<br>NR_113180.1<br>NR_041409.1
**RB115**	KY558694	1360	*Streptomyces lincolnensis* NRRL 2936<br>*Streptomyces cyaneus* NBRC 13346<br>*Streptomyces lincolnensis* NBRC 13054	2374<br>2368<br>2368	98.82%<br>98.45%<br>98.82%	NR_119101.1<br>NR_112525.1<br>NR_041104.1
**RB116**	KY558693	1373	*Streptomyces chattanoogensis* NBRC 12754<br>*Streptomyces sioyaensis* NRRL B-5408<br>*Streptomyces sioyaensis* NBRC 12820	2466<br>2460<br>2460	99.20%<br>99.13%<br>99.13%	NR_112260.1<br>NR_043498.1<br>NR_112289.1
**RB129**	MH044516	993	*Curtobacterium oceanosedimentum* ATCC 31317<br>*Curtobacterium oceanosedimentum* ATCC 31317<br>*Curtobacterium luteum* DSM 20542	1790<br>1790<br>1783	99.80%<br>99.80%<br>99.80%	NR_116064.1<br>NR_104839.1<br>NR_026157.1

[a] Sequence similarities were calculated using method recommended by Meier-Koltfoff [1].

Table S4. Ecologically relevant fungal s used as targets in the bioactivity tests.

Strain #	Strain affiliation; and strain number including GenBank accession number of 18S rRNA gene of the closest relative from BLASTn search	GenBank	Growth on PDA[a]	Inoculation
#1	*Cladosporium* sp.;<br>*C .perangustum* CPC18229(JF499836	KJ817314	Slow	method B
#2	*Cladosporium* sp.;<br>*C. perangustum* CPC18229 (JF499836)	KJ817319	slow	method B
#4	*Pleosporales* sp.;<br>*P.* sp. LH222 (HQ832821)	KJ817321	slow	method B
#5	*Fusarium sp.;*<br>*Fusarium lateritium strain BBA 63665* (AF310982.1)	KJ817322	medium	method B
#7	*Coriolopsis* sp.;<br>*C.* sp. aff17 (EU863193)	KJ817324	medium	method B
#8	*Coriolopsis* sp.;<br>*C.* sp. aff17 (EU863193)	KJ817325	slow	method B
#10	*Fusarium* sp.;<br>*F.equiseti* ATT040 (HQ607811)	KJ817326	slow	method B
#12	*Cunninghamella* sp.;<br>*C. echinulata* var. *nodosa* (AF346407)	KJ817330	Fast (aerial hyphae)	method A
#13	*Cladosporium* sp.;<br>*C.* sp. CLJ-2 (LC373145)	KJ817329	slow	method B
#14	*Umbelopsis* sp.;<br>*Umbelopsis isabellina* UI1 (JF303862)	KJ817309	Fast (aerial hyphae)	method A
#15	*Alternaria* sp.;<br>*A. alternata* ATCC MYA-4642 (HQ263343)	KJ817328	medium	method B
#17	*Trichoderma* sp.;<br>*T.* sp. ATT151 (HQ607860)	KJ817310	medium	method B
#22	*Trichoderma* sp.;<br>*T.* sp. TPK IMEA5 (JF831494)	KJ817316	fast	method A
#24	*Hypocrea* sp.;<br>*H. virens* (anamorph of *Trichoderma virens*) TR039 (HQ608079)	KJ817318	fast	method A
T112	*Termitomyces* sp. from *Odontototermes* cf. *badius* colony Od112	KX250316	slow	method B
T115	*Termitomyces* sp. from *Macrotermes natalensis* colony Mn115	N/A	slow	method B
T153	*Termitomyces* sp. from *Macrotermes natalensis* colony Mn153	KX097054	slow	method B
P5	*Termitomyces* sp. from *Macrotermes natalensis* colony Mn105	JDCH00000000	slow	method B
BB	*Beauveria bassiana* ST 17960 (JMRC)	N/A	slow	method B
MA	*Metarhizium anisopliae* ATCC 24942 (JMRC)	N/A	fast	method B

[a]slow: fungus requires > 7 days to overgrow agar plate; medium: fungus requires 3-7 days to overgrow agar plate; fast: fungus requires 1-3 days to overgrow agar plate.

Table S5. Antimicrobial assay results of extracts of isolated bacteria against eight medically relevant bacteria and fungi (average zone of inhibition given in mm).

Strain ID	Extract ID	cultivation medium	*Bacillus subtilis* ATCC 6633	*Staphylococcus aureus* IMET 10760	*Escherichia coli* SG 458	*Pseudomonas aeruginosa* K799/61	*Mycobacterium vaccae* IMET 10670	*Sporobolomyces salmonicolor* SBUG-549	*Candida albicans* BMSY 212	*Penicillium notatum* JP36
**RB2**	E58	ISP2	0	0	0	0	0	0	0	0
**RB3**	E47	ISP2	15	17	0	0	13	0	0	0
**RB4**	E74	ISP2	0	0	0	0	0	0	0	0
**RB5**	E1	ISP2	10	0	0	10	0	0	0	0
**RB6**	E4	ISP2	0	0	0	0	0	0	0	0
**RB7**	E3	ISP2	14	13	14	0	12	22	22	18
**RB8**	E5	ISP2	0	0	0	0	0	0	0	0
**RB9**	E103	ISP2	10	11	0	10	20	0	0	0
**RB10**	E8	ISP2	0	0	0	0	0	0	0	0
**RB11**	E9	ISP2	0	0	0	0	0	15	0	0
**RB13**	E11	ISP2	12	12	0	0	0	21	22.5	20
**RB14**	E12	ISP2	11	0	0	0	16	0	0	0
**RB14**	E13	PDB	12	14	19	0	19	17.5	0	19
**RB15**	E65	ISP2	0	0	0	0	14	12	15	13
**RB16**	E66	ISP2	0	0	0	0	12	0	0	12
**RB17**	E38	ISP2	19	18.5	14	12	21	13	0	16.5
**RB18**	E68	ISP2	0	0	0	0	0	0	0	0
**RB19**	E69	ISP2	0	0	0	0	0	0	0	0
**RB20**	E16	ISP2	0	0	0	0	0	0	0	0
**RB21**	E17	ISP2	0	0	0	0	0	0	0	0
**RB22**	E18	ISP2	0	0	0	0	0	0	0	0
**RB22**	E19	PDB	0	0	0	0	0	0	0	0
**RB23**	E50	ISP2	12	15	0	0	13	0	0	0
**RB24**	E20	ISP2	0	0	0	0	0	0	0	0
**RB24**	E21	PDB	0	0	0	0	0	0	0	0
**RB25**	E135	ISP2	10	0	0	0	12	11	0	0
**RB26**	E22	ISP2	10	0	0	0	0	0	0	0
**RB26**	E23	PDB	10	0	0	0	0	0	0	0

**RB27**	E26	ISP2	14	13	13	0	13	12	0	14.5
**RB27**	E27	PDB	13	13.5	16	0	21	19	0	15.5
**RB28**	E67	ISP2	0	0	0	0	13	0	0	0
**RB29**	E62	ISP2	14	0	0	0	0	0	0	0
**RB30**	E40	ISP2	20	22	15	14	27	14	0	16
**RB31**	E42	ISP2	0	0	0	0	0	31	28	30
**RB32**	E43	ISP2	0	0	0	0	0	0	0	0
**RB33**	E44	ISP2	0	0	0	0	0	0	0	0
**RB34**	E80	ISP2	0	0	0	0	0	0	0	0
**RB36**	E63	ISP2	0	0	0	0	0	0	0	0
**RB38**	E54	ISP2	11	0	0	0	25	0	0	0
**RB39**	E55	ISP2	0	0	0	0	0	0	0	0
**RB40**	E56	ISP2	13	11	0	0	19	0	0	0
**RB41**	E113	ISP2	19	17.5	14	0	14	0	0	0
**RB42**	E70	ISP2	0	0	0	0	0	0	0	0
**RB43**	E72	ISP2	10	0	0	0	0	0	0	0
**RB44**	E75	ISP2	10	0	21	0	18	21	0	0
**RB45**	E86	ISP2	10	0	13	0	13	18	0	0
**RB47**	E136	ISP2	12	12	0	0	0	0	0	0
**RB48**	E76	ISP2	11	15	0	0	30	24	18	21
**RB50**	E88	ISP2	17	17.5	13	0	28	15	0	0
**RB51**	E106	ISP2	0	0	0	11	0	0	0	0
**RB52**	E91	ISP2	0	0	0	0	12	0	0	0
**RB53**	E93	ISP2	0	0	0	0	0	0	0	0
**RB54**	E94	ISP2	0	0	0	0	0	0	0	0
**RB55**	E95	ISP2	24	22	14	11	36	15	12	0
**RB56**	E104	ISP2	12	13	0	10	28	0	0	0
**RB58**	E114	ISP2	0	0	0	0	0	0	0	0
**RB59**	E107	ISP2	0	0	0	0	0	0	0	0
**RB60**	E105	ISP2	0	0	0	0	0	0	0	0
**RB62**	E109	ISP2	0	0	0	0	0	0	0	0
**RB63**	E96	ISP2	0	0	0	0	0	0	0	0
**RB64**	E97	ISP2	0	0	0	0	0	0	0	0

**RB66**	E98	ISP2	16	27	0	11	20	25	15	21.5
**RB68**	E99	ISP2	16	17	0	0	25.5	0	0	0
**RB71**	E110	ISP2	0	0	0	0	0	0	0	0
**RB72**	E100	ISP2	22	21	0	11	23	48	48	37
**RB74**	E102	ISP2	11	16	0	10	32	0	0	0
**RB76**	E112	ISP2	15.5	16	0	0	16	14	0	0
**RB77**	E118	ISP2	0	0	0	0	0	0	0	0
**RB78**	E119	ISP2	12	12	0	0	0	0	0	0
**RB79**	E120	ISP2	12	13	0	0	0	0	0	0
**RB80**	E121	ISP2	13	13	0	0	0	0	0	0
**RB81**	E122	ISP2	13	11	0	0	12	0	0	0
**RB82**	E123	ISP2	11	11	0	0	24	0	0	0
**RB83**	E124	ISP2	11	10	0	0	12	0	0	0
**RB85**	E126	ISP2	12	11	10	0	13	0	0	0
**RB86**	E127	ISP2	11	10	15	0	17	17	0	0
**RB87**	E128	ISP2	11	11	0	0	14	0	0	0
**RB88**	E129	ISP2	10	0	0	0	0	13	0	0
**RB89**	E130	ISP2	10	0	0	0	0	0	0	0
**RB90**	E77	ISP2	20	20	12	0	20	13	12	15
**RB91**	E49	ISP2	12	11	0	0	32	21	0	0
**RB92**	E144	ISP2	11	11	0	0	0	13	0	12
**RB93**	E148	ISP2	14.5	13.5	0	16	20	0	0	0
**RB94**	E61	ISP2	33.5	32	13	21	40	15	15	13
**RB95**	E149	ISP2	11	11	0	0	18	0	0	0
**RB96**	E150	ISP2	11	0	0	10	21	0	0	0
**RB97**	E151	ISP2	10	0	0	0	0	0	0	0
**RB98**	E57	ISP2	12	12.5	0	10	24	16	0	11
**RB99**	E60	ISP2	11	11	0	10	13	23	0	0
**RB100**	E48	ISP2	19	20	23	10	22	24	13	12
**RB101**	E152	ISP2	12	13.5	0	10	18	12	0	0
**RB102**	E143	ISP2	20	19	0	0	20	19	24	17.5
**RB103**	E153	ISP2	11	12	0	0	28	0	0	0
**RB104**	E59	ISP2	11	11	0	10	18	20	0	12

**RB105**	E145	ISP2	18	15	0	0	33	0	0	11
**RB106**	146	ISP2	12	11.5	0	0	33	0	0	11
**RB107**	E46	ISP2	11	12	0	10	19	0	0	0
**RB108**	E45	ISP2	0	0	0	0	11	19	19	19
**RB109**	E147	ISP2	15	15	0	0	26.5	0	0	20
**RB110**	E142	ISP2	18	16	0	21	17	15	14	14.5
**RB111**	E154	ISP2	10	13	0	0	11	0	0	0
**RB112**	E155	ISP2	0	0	0	0	0	0	0	0
**RB113**	E35	ISP2	12	15	13	10	21.5	0	0	0
**RB114**	E78	ISP2	10	11	0	0	0	0	0	0
**RB115**	E36	ISP2	0	0	0	0	0	0	0	0
**RB116**	E37	ISP2	0	0	15	0	17	24	18	23.5
**RB129**	E41	ISP2	0	0	0	0	15	0	0	0

Table S6. Antifungal assay results of extracts of isolated bacteria against ecologically-relevant fungi # 1 - #24 (T115: *Termitomyces* sp. from *Macrotermes natalensis* (Mn) colony Mn115, T112: *Termitomyces* sp. from *Odontototermes badius* colony Od112, T153: *Termitomyces* sp. from Mn colony Mn153, P5: *Termitomyces* sp. from Mn colony Mn105, MA - *Metarhizium anisopliae* ATCC 24942, BB *Beauveria bassiana* ST 17960- (I= very weak inhibition, without measurable zone of inhibition SI= strong inhibition, inhibition zone not measurable; average in mm given).

**Extract ID**	#1	#2	#4	#5	#8	#10	#12	#13	#15	#17	#22	#24	**T115**	**T112**	**T153**	**P5**	**MA**	**BB**
**E3**	0	0	8	9	0	0	0	0	5	0	0	0	5	23	23	16	13	9
**E9**	0	0	0	0	0	0	0	0	0	0	0	0	0	0	0	0	0	0
**E11**	6	0	9	10	0	0	0	0	5	0	0	0	13	2	2	17	12	10
**E12**	0	0	0	0	0	0	0	0	0	0	0	0	0	0	0	0	0	0
**E13**	0	0	0	0	0	0	0	0	0	0	0	0	0	0	0	0	0	0
**E26**	0	0	0	0	0	0	0	0	0	0	0	0	0	0	0	0	0	0
**E27**	0	0	0	0	0	0	0	0	0	0	0	0	0	0	0	0	0	0
**E37**	0	0	0	0	0	0	0	0	0	0	0	0	0	0	0	0	0	0
**E38**	0	0	0	0	0	0	0	0	0	0	0	0	0	0	0	0	0	0
**E40**	0	0	0	0	0	0	0	0	0	0	0	0	0	0	0	0	0	0
**E42**	6	0	15	13	0	13	0	10	9	0	0	0	15	15	12	12	12	13
**E45**	0	0	0	0	0	0	0	0	0	0	0	0	0	0	0	0	0	0
**E48**	0	0	0	0	0	0	0	0	0	0	0	0	0	0	0	0	0	0
**E49**	0	0	0	0	0	0	0	0	0	0	0	0	0	0	0	0	0	0
**E57**	0	0	0	0	0	0	0	0	0	0	0	0	0	0	0	0	0	0
**E59**	0	0	0	0	0	0	0	0	0	0	0	0	0	0	0	0	0	0
**E60**	0	0	0	0	0	0	0	0	0	0	0	0	0	0	0	0	0	0
**E61**	0	0	0	0	0	0	0	0	I	0	0	0	0	0	0	0	0	0
**E65**	0	0	0	0	0	0	0	0	0	0	0	0	0	0	0	0	0	0
**E66**	0	0	0	0	0	0	0	0	0	0	0	0	0	0	0	0	0	0
**E75**	0	0	0	0	0	0	0	0	0	0	0	0	0	0	0	0	0	0
**E76**	0	0	0	0	0	0	0	0	0	0	0	0	0	0	0	0	0	0
**E77**	0	0	0	0	0	0	0	0	0	0	0	0	0	0	0	0	0	0
**E86**	0	0	0	0	0	0	0	0	0	0	0	0	0	0	0	0	0	0
**E88**	0	0	0	0	0	0	0	0	0	0	0	0	0	0	0	0	0	0
**E95**	0	0	0	0	0	0	0	0	0	0	0	0	0	0	0	0	0	0
**E98**	0	0	0	0	0	0	0	0	0	0	0	0	0	0	0	0	0	0

**E100**	18	0	31	8	0	0	0	0	10	5	0	0	0	23	10	I	I	I
**E127**	0	0	0	0	0	0	0	0	0	0	0	0	0	0	0	0	0	0
**E129**	0	0	0	0	0	0	0	0	0	0	0	0	0	0	0	0	0	0
**E131**	0	0	0	0	0	0	0	0	0	0	0	0	0	0	0	0	0	0
**E135**	0	0	0	0	0	0	0	0	0	0	0	0	0	0	0	0	0	0
**E136**	0	0	0	0	0	0	0	0	0	0	0	0	0	0	0	0	0	0
**E142**	0	0	0	0	0	0	0	0	0	0	0	0	0	0	0	0	0	0
**E143**	0	0	0	0	0	0	0	0	0	0	0	0	0	0	0	0	0	0
**E144**	0	0	0	0	0	0	0	0	0	0	0	0	0	0	0	0	0	0
**E145**	0	0	0	0	0	0	0	0	0	0	0	0	0	0	0	0	0	0
**E146**	0	0	0	0	0	0	0	0	0	0	0	0	0	0	0	0	0	0
**E147**	0	0	0	0	0	0	0	0	0	0	0	0	0	0	0	0	0	0
**Amp.B 1**	SI	0	SI	10	0	0	I	0	11	13	0	I	I	I	I	I	10	11
**Amp.B 2**	SI	0	SI	16	0	0	I	0	11	10	0	I	I	I	I	I	9	8
**Cyclo. 1**	SI	0	SI	33	0	23	I	28	24	17	0	I	I	I	I	I	15	15
**Cyclo. 2**	SI	0	SI	32	0	0	I	31	25	20	0	I	I	I	I	I	15	18

Amp.: amphotericin B; Cyclo.: cycloheximide

Table S7. Antimicrobial activity of *cyclo*(*N*Me-L-3,5-dichlorotyrosine-Dhb) (**11**, 0.1 mg/mL in MeOH), ciprofloxacin (cip.) and amphotericin B (amp.) towards Gram (+) and Gram (–) bacteria and fungi.[a]

**compd.**	***B.subtilis*** **ATCC 6633**	***S. aureus*** **IMET 10760**	***E.coli*** **SG 458**	***P. aeruginosa*** **SG137**	***P. aeruginosa*** **K799/61**	**MRSA** ***S.sureus*** **134/93**	**VRSA** ***E. faecalis*** **1528**	***M.vaccae*** **IMET 10670**	***S. salmonic.*** **SBUG 549**	***C.albicans*** **SBSY 212**	***P. notatum*** **JP36**
**11**	10	0	0	0	10	0	10	10	0	0	0
cip.	29	19	24/32p	26	26/34p	0	17F	21p			
amp.									18p	21p	19p

[a]: The value indicated the diameter of inhibition zone (in mm).

Table S8. Representative images of antifungal paper disc assay against co-isolated fungi, the fungal cultivar and insectpathogenic fungi.

**Fungus**	**Extracts E3 – E38**	**Negative control**[a]	**Positive control**[b]
**#1** *Cladosporium sp.* (KJ817314)	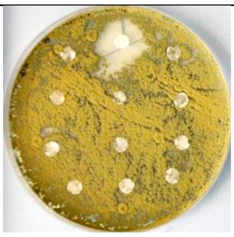	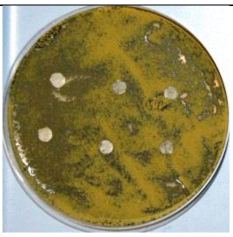	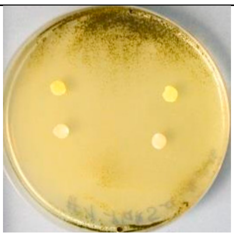
**#2** *Cladosporium* sp.(KJ817319)	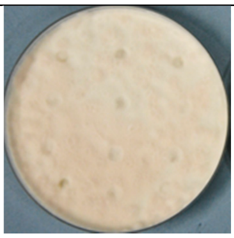	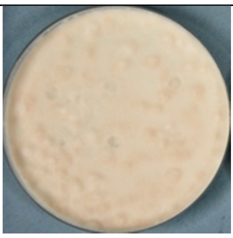	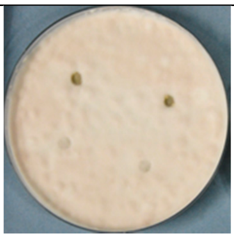
**#4** *Pleosporales* sp. (KJ817321)	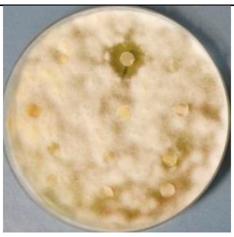	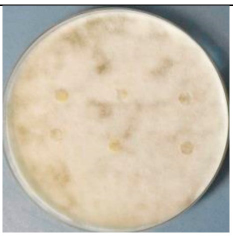	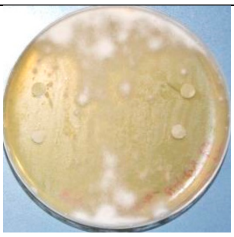
**#5** *Fusarium* sp. (KJ817322)	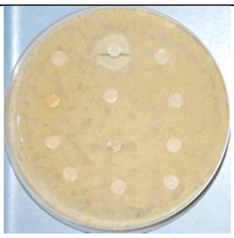	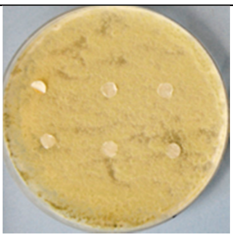	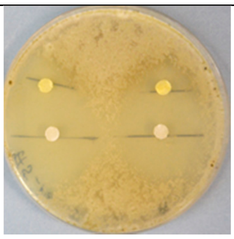
**#8** *Coriolopsis* sp. (KJ817325)	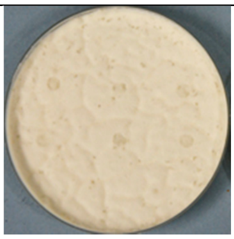	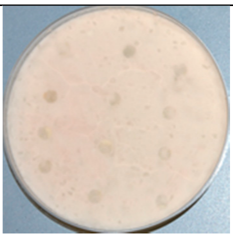	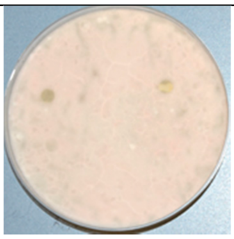
**#10** *Fusarium* sp. (KJ817326)	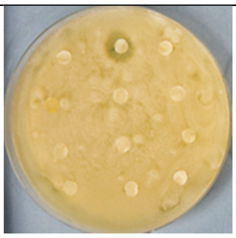	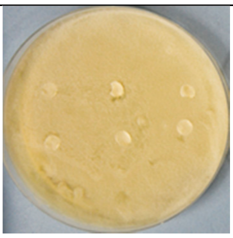	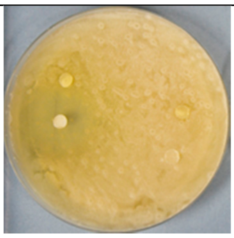

**#12**<br>*Cunninghamella* sp. (KJ817330)	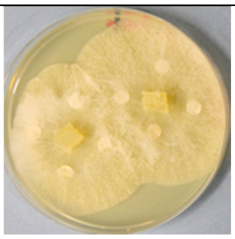	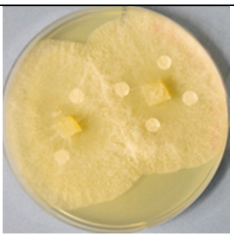	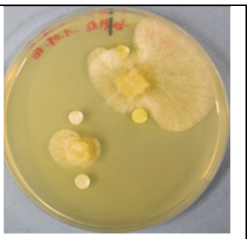
**#13**<br>*Cladosporium* sp. CLJ-2 (KJ817329)	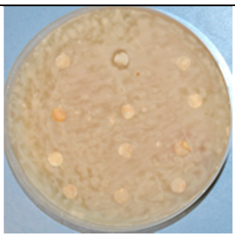	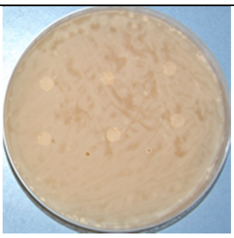	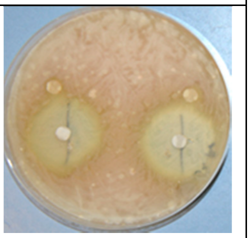
**#15**<br>*Alternaria* sp. (KJ817328)	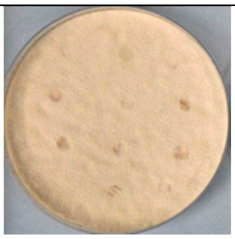	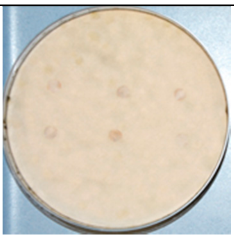	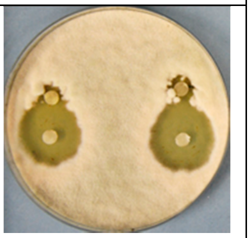
**#17**<br>*Trichoderma* sp. (KJ817310)	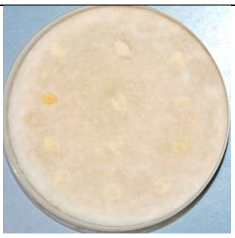	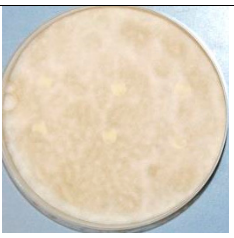	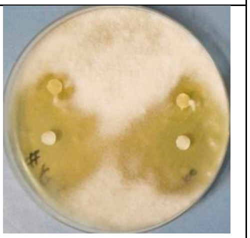
**#22**<br>*Trichoderma* sp. (KJ817316)	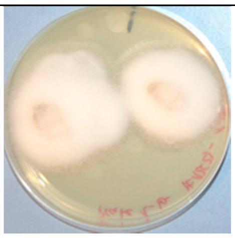	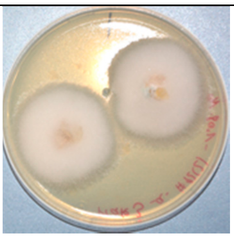	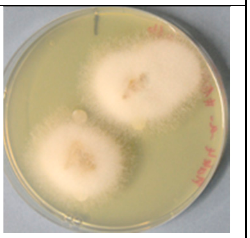
**#24**<br>*Hypocrea* sp. (KJ817318)	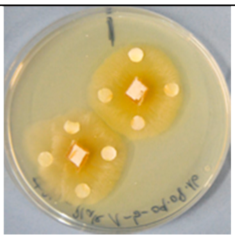	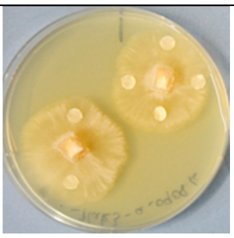	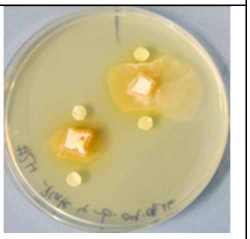
**BB**<br>*Beauveria bassiana* ST 17960	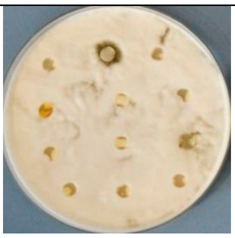	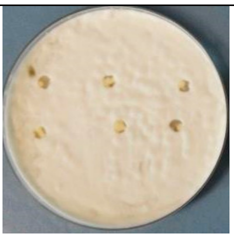	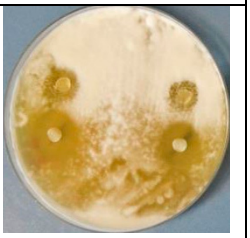

**MA**<br><br>*Metarhizium anisopliae* ATCC 24942	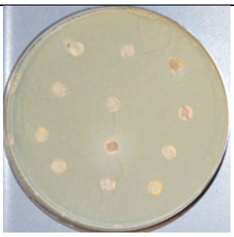	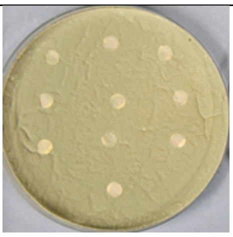	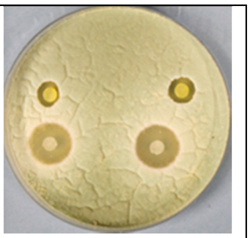
**P5**<br><br>*Termitomyces* sp. from *Macrotermes natalensis* colony Mn105	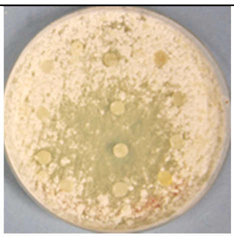	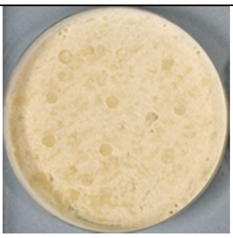	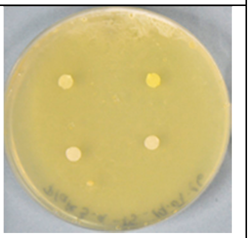
**T112**<br><br>*Termitomyces* sp. from *Odontototermes* cf. *badius* colony Od112	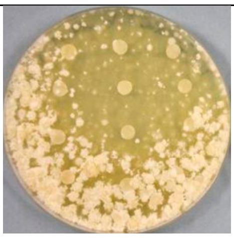	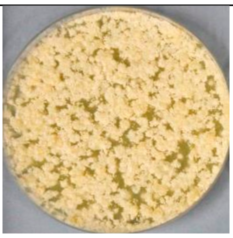	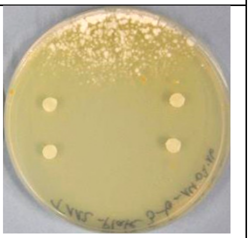
**T115**<br><br>*Termitomyces* sp. from *Macrotermes natalensis* colony Mn115	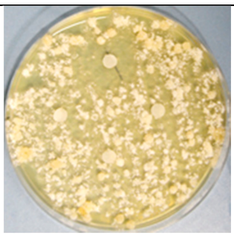	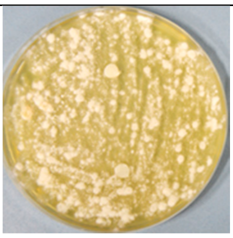	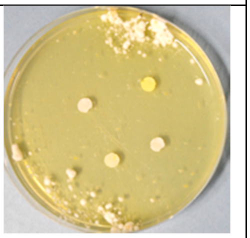
**T153**<br><br>*Termitomyces* sp. from *Macrotermes natalensis* colony Mn153	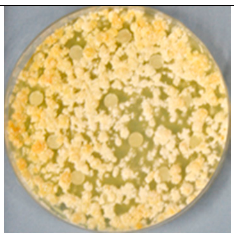	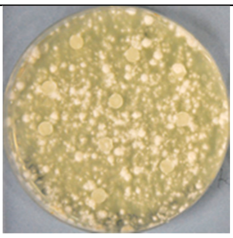	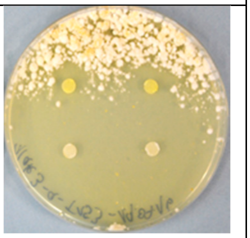

[a)] Negative control (paper discs + MeOH); [b)] positive control (Amphotericin B (upper row) and cycloheximide (lower row).

Table S9. Representative co-cultivation of *Streptomyces* sp. RB108 against co-isolated fungi.

Strain	Inhibition	Control	Co-culture	Method	Time
**#1** *Cladosporium sp.* (KJ817314)	moderate	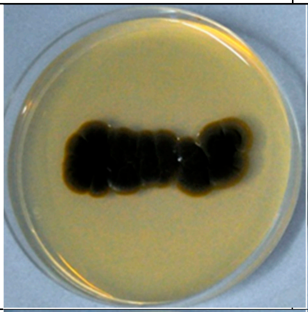	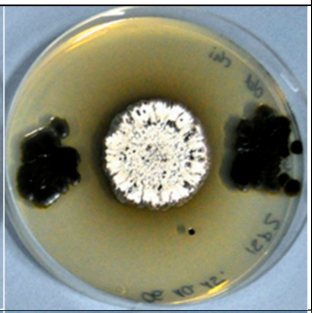	B	7-day
**#4** *Pleosporales sp.* (KJ817321)	moderate	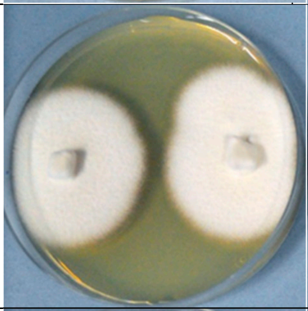	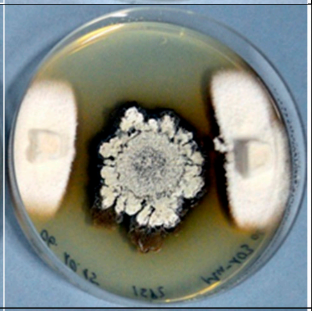	B	7-day
**#7** *Coriolopsis sp.* (KJ817324)	moderate	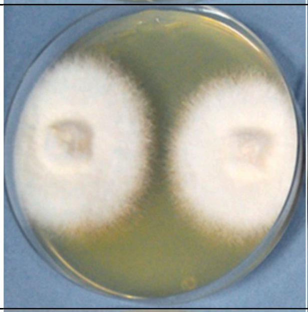	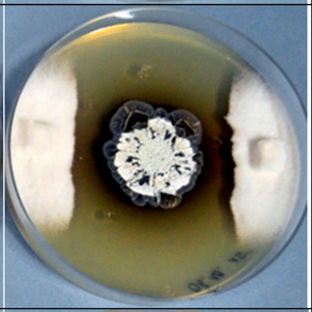	B	7-day
**#8** *Coriolopsis sp.* (KJ817325)	weak	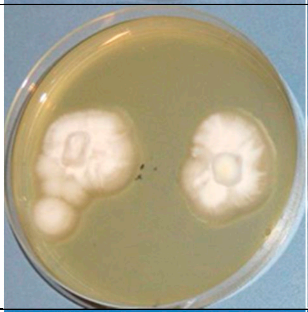	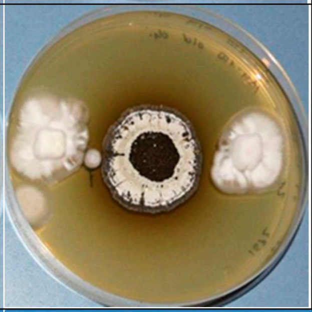	B	7-day
**#12** *Cunninghamella* sp. (KJ817330)	no	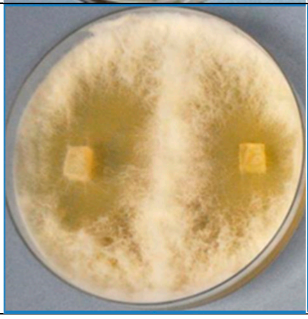	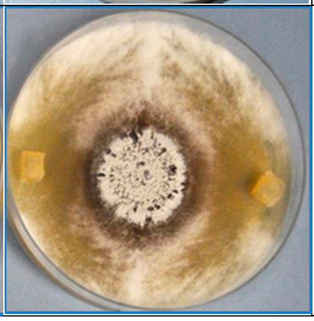	B	7-day

**#13**<br>*Cladosporium sp.*<br>CLJ-2 (KJ817329)	moderate	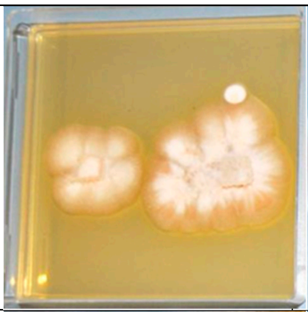	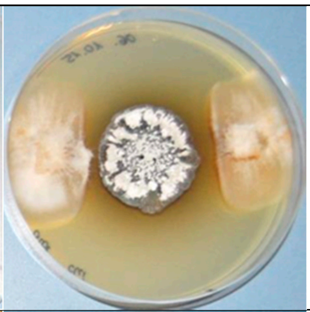	B	7-day
**#14**<br>*Umbelopsis* sp.<br>(KJ817309)	weak	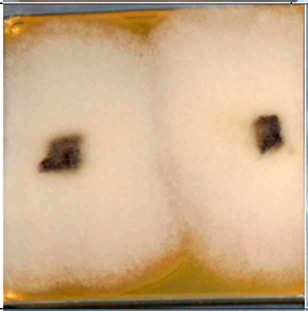	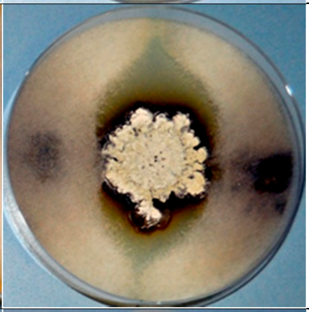	B	7-day
**#15**<br>*Alternaria* sp.<br>(KJ817328)	moderate	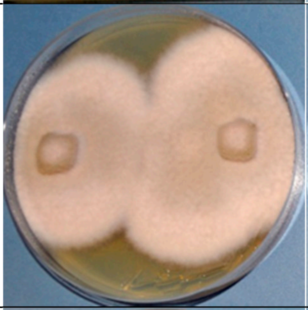	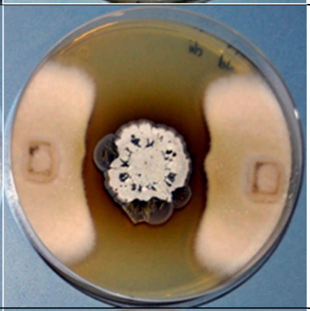	B	7-day
**#17**<br>*Trichoderma* sp.<br>(KJ817310)	moderate	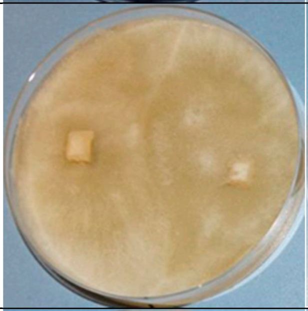	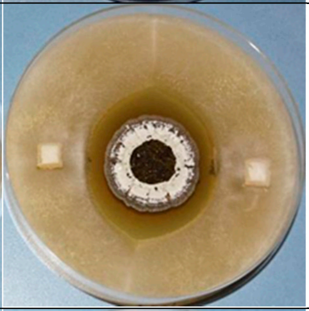	B	7-day
**#22**<br>*Trichoderma sp.*<br>(KJ817316)	weak	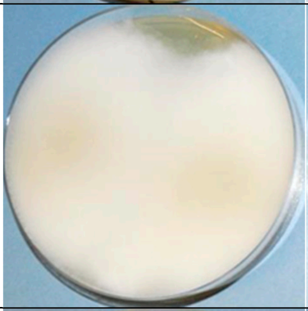	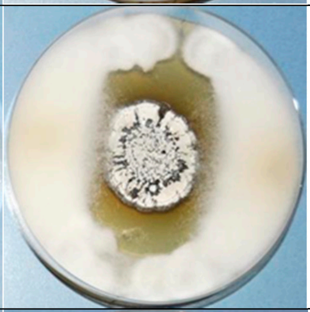	B	7-day
**BB**<br>*Beauvaria bassiana*<br>ST 17960	no	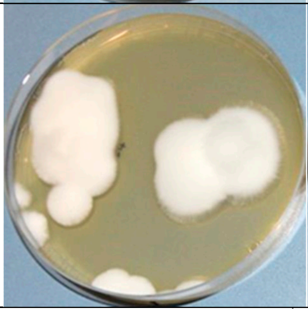	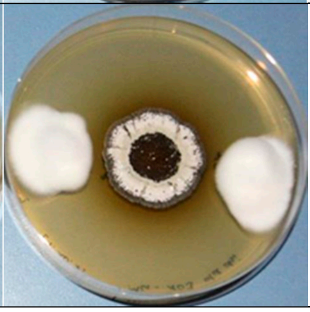	B	7-day

**MA**<br>*Metarhizium anisopliae* ATCC 24942	weak	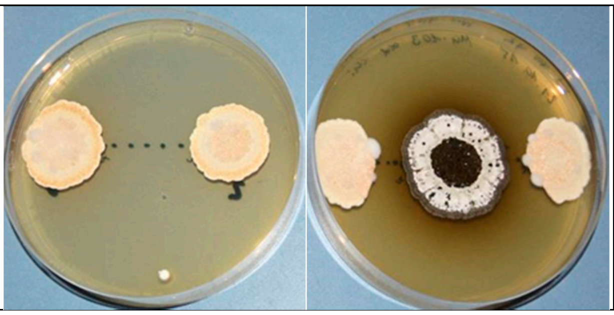	B	7-days

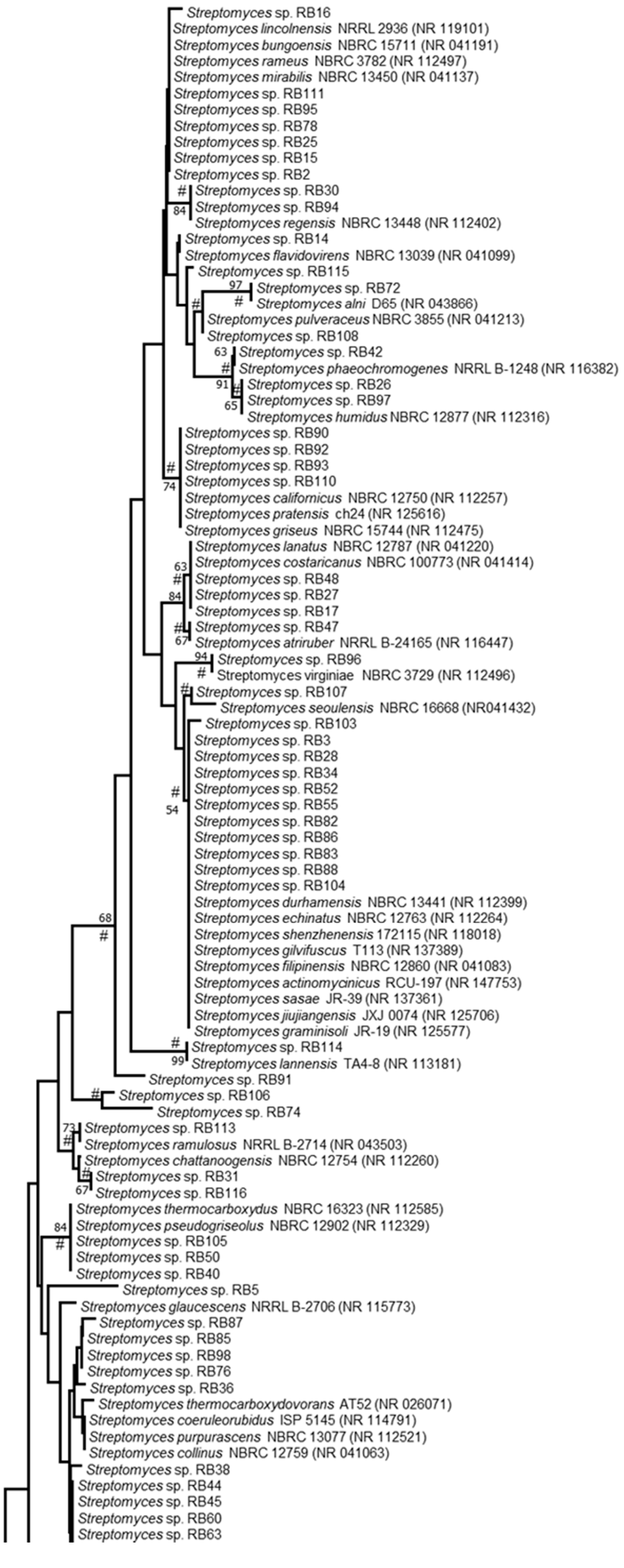
Streptomyces sp. RB16
Streptomyces lincolnensis NRRL 2936 (NR 119101)
Streptomyces bungoensis NBRC 15711 (NR 041191)
Streptomyces rameus NBRC 3782 (NR 112497)
Streptomyces mirabilis NBRC 13450 (NR 041137)
Streptomyces sp. RB111
Streptomyces sp. RB95
Streptomyces sp. RB78
Streptomyces sp. RB25
Streptomyces sp. RB15
Streptomyces sp. RB2
Streptomyces sp. RB30
Streptomyces sp. RB94
Streptomyces regensis NBRC 13448 (NR 112402)
Streptomyces sp. RB14
Streptomyces flavidovirens NBRC 13039 (NR 041099)
Streptomyces sp. RB115
Streptomyces sp. RB72
Streptomyces alni D65 (NR 043866)
Streptomyces pulveraceus NBRC 3855 (NR 041213)
Streptomyces sp. RB108
Streptomyces sp. RB42
Streptomyces phaeochromogenes NRRL B-1248 (NR 116382)
Streptomyces sp. RB26
Streptomyces sp. RB97
Streptomyces humidus NBRC 12877 (NR 112316)
Streptomyces sp. RB90
Streptomyces sp. RB92
Streptomyces sp. RB93
Streptomyces sp. RB110
Streptomyces californicus NBRC 12750 (NR 112257)
Streptomyces pratensis ch24 (NR 125616)
Streptomyces griseus NBRC 15744 (NR 112475)
Streptomyces lanatus NBRC 12787 (NR 041220)
Streptomyces costaricanus NBRC 100773 (NR 041414)
Streptomyces sp. RB48
Streptomyces sp. RB27
Streptomyces sp. RB17
Streptomyces sp. RB47
Streptomyces atriruber NRRL B-24165 (NR 116447)
Streptomyces sp. RB96
Streptomyces virginiae NBRC 3729 (NR 112496)
Streptomyces sp. RB107
Streptomyces seoulensis NBRC 16668 (NR041432)
Streptomyces sp. RB103
Streptomyces sp. RB3
Streptomyces sp. RB28
Streptomyces sp. RB34
Streptomyces sp. RB52
Streptomyces sp. RB55
Streptomyces sp. RB82
Streptomyces sp. RB86
Streptomyces sp. RB83
Streptomyces sp. RB88
Streptomyces sp. RB104
Streptomyces durhamensis NBRC 13441 (NR 112399)
Streptomyces echinatus NBRC 12763 (NR 112264)
Streptomyces shenzhenensis 172115 (NR 118018)
Streptomyces gilvifuscus T113 (NR 137389)
Streptomyces filipinensis NBRC 12860 (NR 041083)
Streptomyces actinomycinicus RCU-197 (NR 147753)
Streptomyces sasae JR-39 (NR 137361)
Streptomyces jiujiangensis JXJ 0074 (NR 125706)
Streptomyces graminisoli JR-19 (NR 125577)
Streptomyces sp. RB114
Streptomyces lannensis TA4-8 (NR 113181)
Streptomyces sp. RB91
Streptomyces sp. RB106
Streptomyces sp. RB74
Streptomyces sp. RB113
Streptomyces ramulosus NRRL B-2714 (NR 043503)
Streptomyces chattanoogensis NBRC 12754 (NR 112260)
Streptomyces sp. RB31
Streptomyces sp. RB116
Streptomyces thermocarboxydus NBRC 16323 (NR 112585)
Streptomyces pseudogriseolus NBRC 12902 (NR 112329)
Streptomyces sp. RB105
Streptomyces sp. RB50
Streptomyces sp. RB40
Streptomyces sp. RB5
Streptomyces glaucescens NRRL B-2706 (NR 115773)
Streptomyces sp. RB87
Streptomyces sp. RB85
Streptomyces sp. RB98
Streptomyces sp. RB76
Streptomyces sp. RB36
Streptomyces thermocarboxydovorans AT52 (NR 026071)
Streptomyces coeruleorubidus ISP 5145 (NR 114791)
Streptomyces purpurascens NBRC 13077 (NR 112521)
Streptomyces collinus NBRC 12759 (NR 041063)
Streptomyces sp. RB38
Streptomyces sp. RB44
Streptomyces sp. RB45
Streptomyces sp. RB60
Streptomyces sp. RB63
84
97
63
91
65
74
63
84
67
94
54
68
99
73
67
84

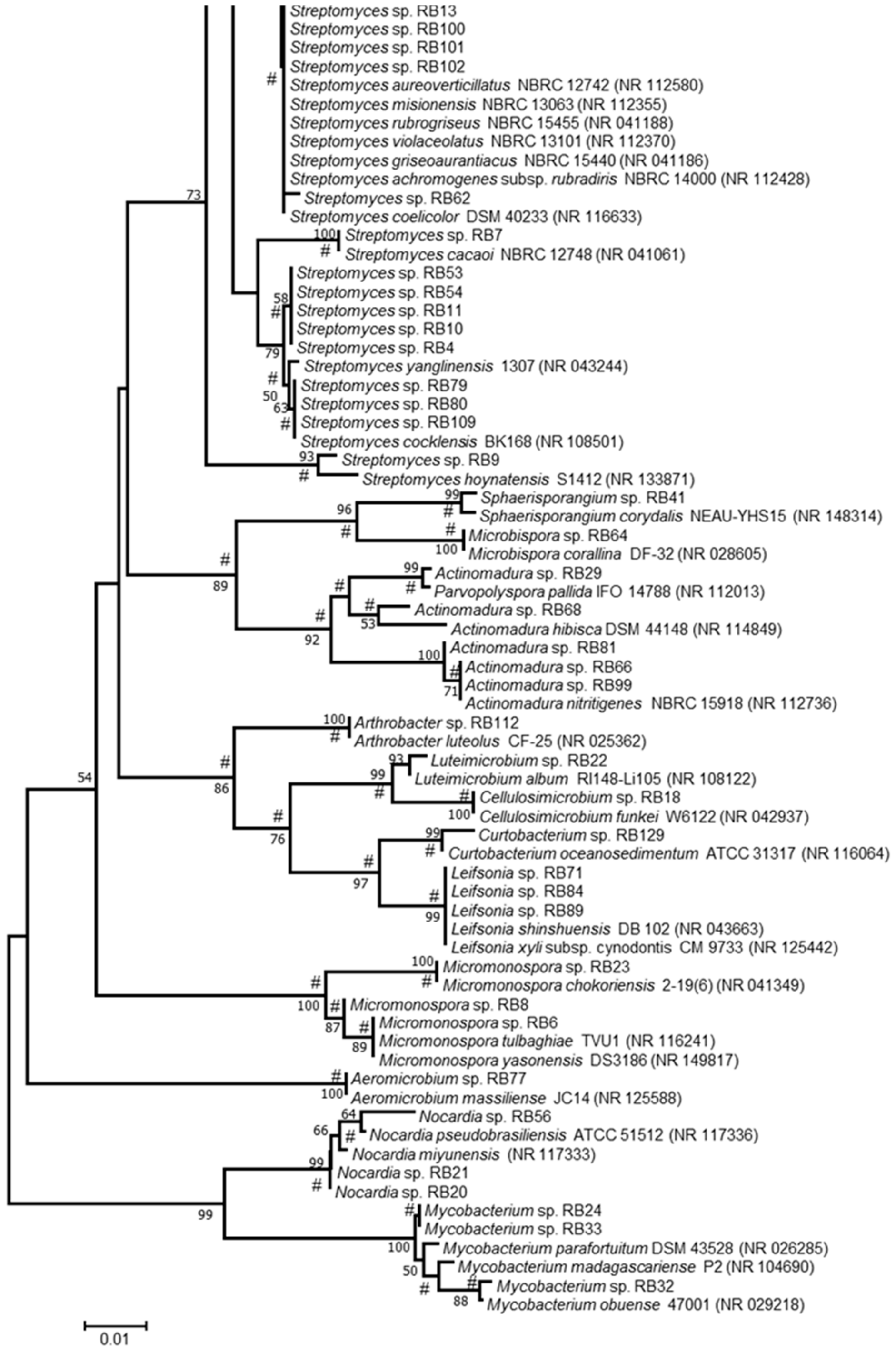

Figure S1. Unrooted Neighbour-joining tree based on near-complete 16S rRNA gene sequences showing relationships between isolated Actinobacteria and closest relatives. Hashtags indicate branches that were also recovered in the maximum-likelihood tree. Only bootstrap values above 50% (based on 1000 pseudoreplicates) are shown. The scale bar indicates 0.01 substitutions per nucleotide position.

Table S10. Presence/absence of transcripts identified in gut microbiome metatranscriptome data from major old worker guts of *M. natalensis* Mn156 and *Odontotermes* sp. Od127.[a]

Gene	Function	Mn156	Od127
alaS	Alanyl-tRNA synthetase	+	+
aspS	Aspartyl-tRNA synthetase	+	-
dnaX	DNA polymerase III gamma-tau subunits	+	-
topA	DNA topoisomerase I	+	-
rpoB	DNA-directed RNA polymerase beta chain	-	-
fus	Elongation factor G	-	-
tufA	Elongation factor Tu	+	+
infB	Initiation factor 2	+	+
glnS	Glutamyl-tRNA synthetase	+	+
metS	Methionyl-tRNA synthetase	+	+
rpl2	Ribosomal protein L2	+	+
rpsE	Ribosomal protein S5	+	+
rpsH	Ribosomal protein S8	+	+
rpsK	Ribosomal protein S11	+	+
zhrS	Threonyl-tRNA synthetase	+	+

a) Transcriptomic analysis: Due to the high isolation rate of Actinobacteria from the gut fluid of termite workers, a mostly microaerophilic or anaerobic gut environment, we analyzed the transcript levels of fifteen Actinobacteria-specific house-keeping genes in our gut microbiome metatranscriptome data from the termite species *Macrotermes natalensis* and *Odontotermes* sp. Gut microbiome transcripts were collected from old major worker gut metatranscriptome data of *M. natalensis* colony Mn156 and *Odontotermes* sp. colony Od127. Transcripts were aligned to the Pfam database (v30.0) by HMMER (v3.1b2) [2] with e-value < 1e-6 and the best matched domain were taken, followed the extraction of transcripts matched to protein domain of the bacterial housekeeping gene Alanyl-tRNA synthetase (PF00152), Aspartyl-tRNA synthetase (PF11734), Glutamyl-tRNA synthetase (PF00749), Methionyl-tRNA synthetase (PF09334), Threonyl-tRNA synthetase (PF00587), Initiation factor 2 (PF11987), Elongation factor G (PF07299), Elongation factor Tu (PF03144), Ribosomal protein L2 (PF00181, PF03947), Ribosomal protein S5 (PF03719, PF00333), Ribosomal protein S8 (PF00410), Ribosomal protein S11 (PF00411), DNA-directed RNA polymerase beta chain (PF03431), DNA polymerase III gamma-tau subunits (PF12169) and DNA topoisomerase I (PF01131). Extracted transcripts were aligned to Nucleotide collection database (nt) of NCBI by BLASTn (e-value < 1e-5) and taken as transcripts of housekeeping gene coded by Actinobacteria if the best matched sequence was from the phylum Actinobacteria. The absence/presence of Actinobacteria housekeeping genes domain were then counted.

Overall, thirteen of the fifteen analyzed house-keeping genes were identified in the *M. natalensis* transcriptome and ten identified in *Odontotermes* sp. transcriptome data. However transcript levels were particularly low indicating towards very only low metabolic activity within the gut.

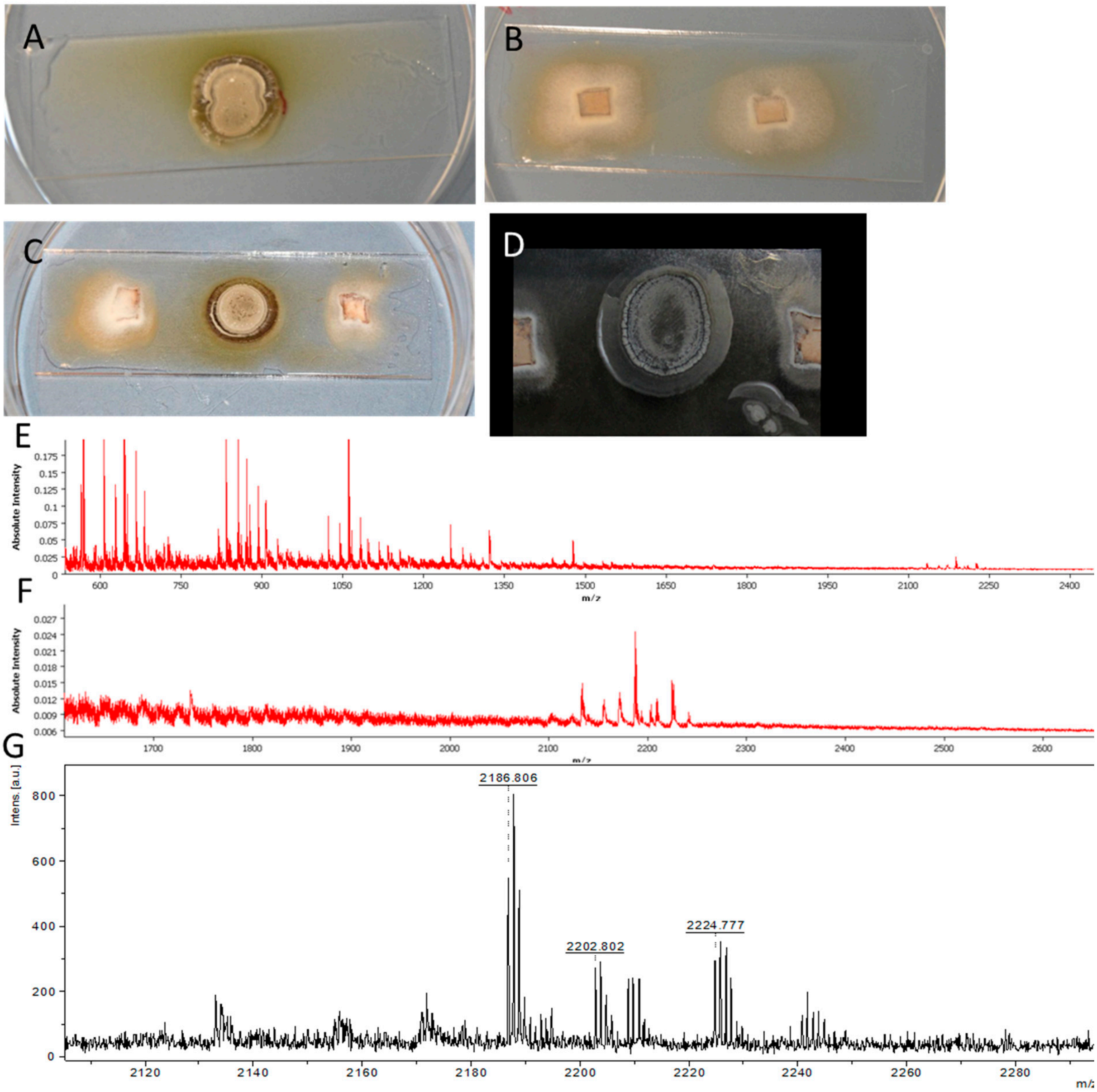

Figure S2. Indium tin oxide (ITO) coated glass slide with 1 ml of ISP2 medium and inoculated with A) *Streptomyces* sp. RB108 (after 7 days); B) *Pleosporales* sp. #4; C). co-culture of *Streptomyces* sp. RB108 and *Pleosporales* sp. #4 ; D) optical picture of the challenge assay between *Streptomyces* sp. RB108 and *Pleosporales* sp. #4 after drying. E) Average MS spectra of the MALDI Imaging MS analysis (TIC normalization). F) Zoom in the region of the average MS spectra between *m/z* 1700-2500 showing the up-regulated RiPPs. G) Zoom in the region of the average MS spectra between *m/z* 2100-2300 showing the up-regulated RiPPs.

Table S11. Rubromidin biosynthetic protein annotations based on sequence homology.

locus tag	Protein	size (aa)	short name	proposed function	closest homolog(s)[a]	identity (%) / alignment length	accession number
**RS25505**	WP_103565560	234		TetR/AcrR family transcriptional regulator	TetR/AcrR family transcriptional regulator	70/78	WP019631302
**RS25510**	WP_103565561	277		TIGR02452 family protein	TIGR02452 family protein	71/99	WP_075898259
**RS25515**	WP_103565562	381		DUF3152 domain-containing protein	DUF3152 domain-containing protein	64/95	WP_051107817
**RS25520**	WP_103565563	455		3' terminal RNA ribose 2'-O-methyltransferase Hen1	3' terminal RNA ribose 2'-O-methyltransferase Hen1	76/100	WP_026400644
**RS25525**	WP_103565564	846		polynucleotide kinase-phosphatase	polynucleotide kinase-phosphatase	85/100	WP_019631305
**RS25530**	WP_103565565	87		hypothetical protein			
**RS25535-50**	WP_103565566	613		ATP-binding cassette domain-containing protein	daunorubicin resistance protein DrrA family ABC transporter ATP-binding protein	68/52	WP_030367886
**RS25555**	WP_103565567	295	RumX	Nif11 family protein	G339_RS0112020[c]<br>CinX	66/98<br>50/98	WP_019631330<br>CAD60522
**RS25560**	WP_103565568	1066	RumM	type 2 lantipeptide synthetase	G339_RS0112025[c]<br>CinM	76/99<br>58/99	WP_019631331<br>CAD60521
**RS25565**	WP_103565569	80	RumA	cinnamycin family lantibiotic	G339_RS0112030<br>CinA[c]	80/98<br>51(98	WP_019631332<br>CAD60520
**RS25570**	WP_103565570	123	RumN	hypothetical protein	G339_RS0112035[c]<br>Cinorf7<br>DivN	74/99<br>49/97<br>37/86	WP_019631333<br>CAD60519<br>ARD09209
**RS25575**	WP_103565571	113		hypothetical protein			
**RS25580**	WP_103565572	274		carbohydrate ABC transporter permease	carbohydrate ABC transporter permease	68/97	WP_026313950
**RS25585**	WP_103565891	295		sugar ABC transporter permease	cellobiose transport system permease protein	66/99	SNR90611
**RS25590**	WP_103565573	414		carbohydrate ABC transporter substrate-binding protein	Maltose-binding periplasmic proteins/domains	60/94	CND69635
**RS25595**	WP_103565574	528		DUF3459 domain-containing protein	alpha-amylase	73/98	PKK14679
**RS25600**	WP_103565575	221		nucleotidyltransferase	hypothetical protein SAMN05443665_1007127	72/96	SNS66748
**RS25605**	WP_103565893	253		putative nucleotidyltransferase	Predicted nucleotidyltransferase	68/98	SPT49826
**RS25610**		102		hypothetical protein			
**RS25615**	WP_103565576	384		hypothetical protein			
**RS25620**	WP_103565577	213		hypothetical protein			

[a] For RS25555–70, only homologs from biosynthetic gene clusters encoding for characterized compounds were considered. Origin of gene clusters: Cin= *Streptomyces cinnamoneous*; Div= *Prochloron didemni*, cinnamycin B cluster from *Actinomadura atramentaria* DSM43919

[b] Percent alignment and identify were determined using BLASTp, following default parameters. Percent alignment is the proportion of the Rum query sequence that aligns to each homolog. [c] Locus taq of cinnamycin B cluster from *Actinomadura atramentaria* DSM43919 as already described [3].

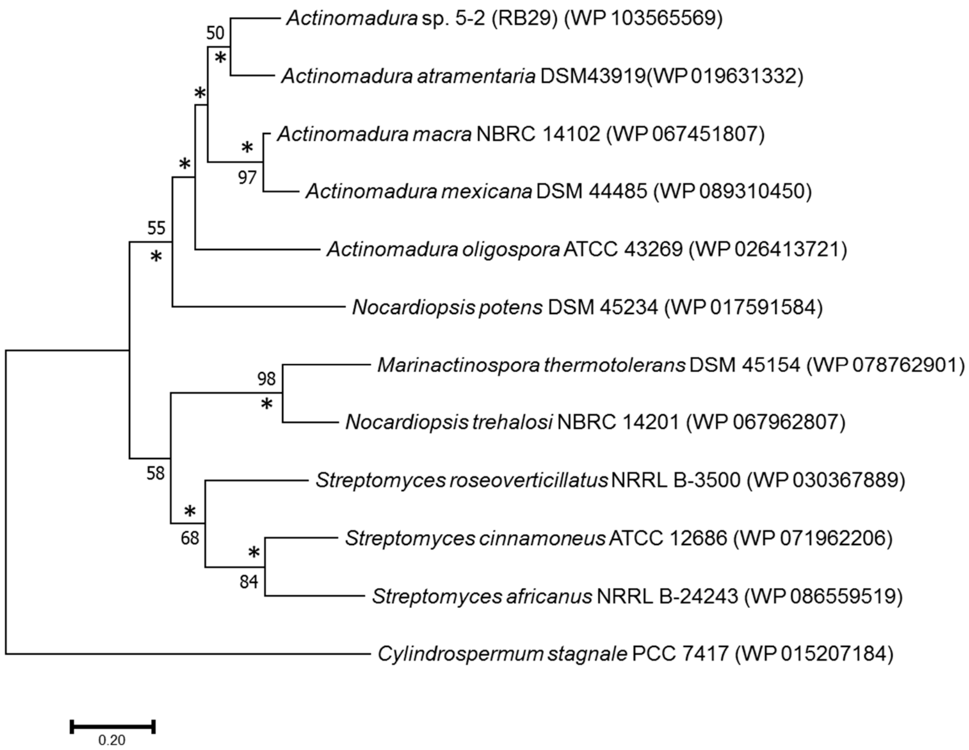

Figure S3. Neighbour-joining tree based on peptide sequence of precursor peptide sequence (RumA, WP 103565569).Star indicate branches that were also recovered in the maximum-likelihood tree. Only bootstrap values above 50% (based on 1000 pseudoreplicates) are shown. The scale bar indicates 0.2 substitutions per amino acid position.

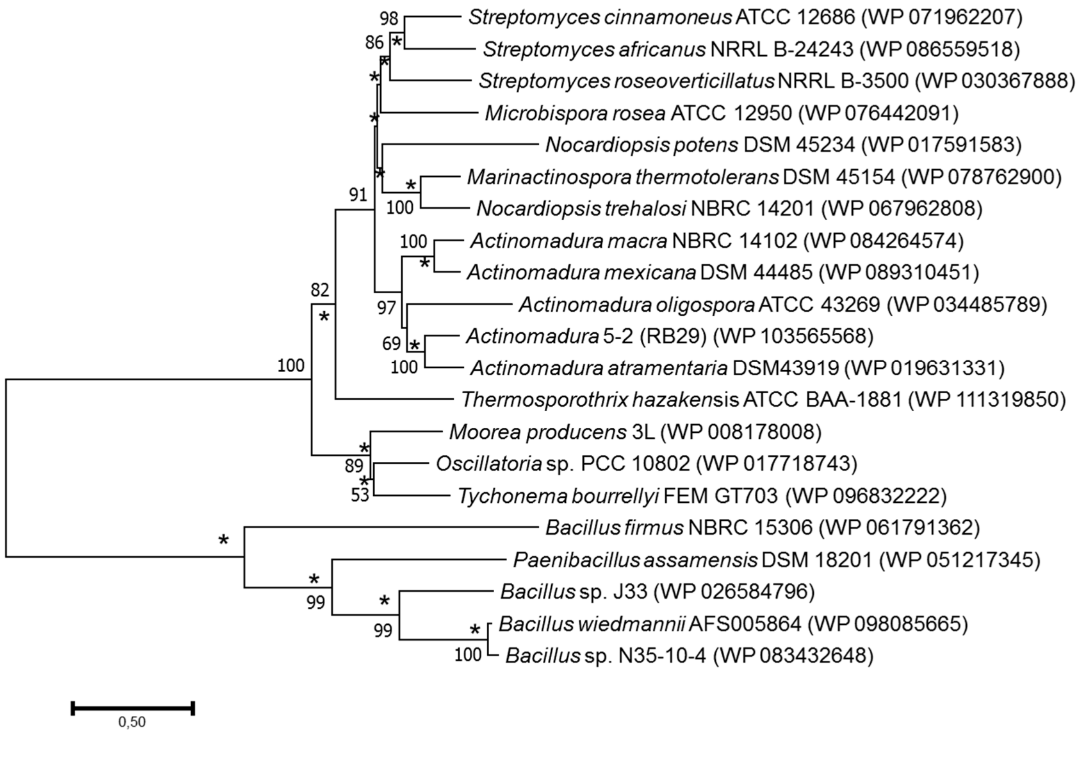

Figure S4. Neighbour-joining tree based on type 2 lantipeptide synthetase sequence RumM. Star indicates branches that were also recovered in the maximum-likelihood tree. Only bootstrap values above 50% (based on 1000 pseudoreplicates) are shown. The scale bar indicates 0.5 substitutions per amino acid position.

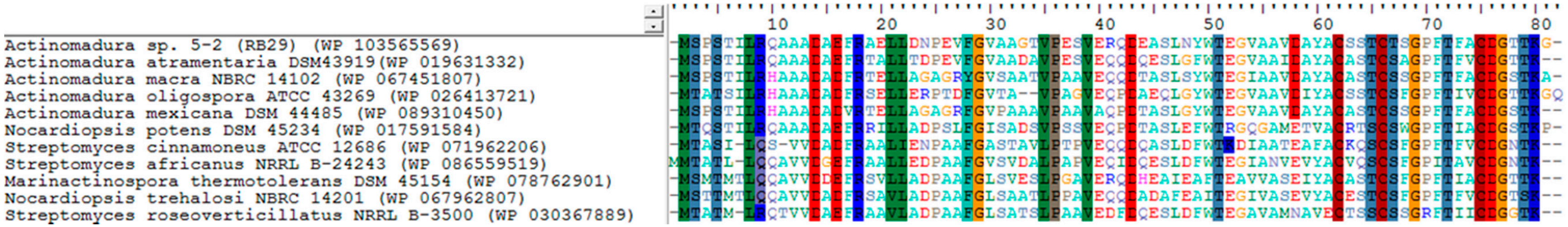

Figure S5. Comparative sequence alignment of precursor peptide sequence (RumA, WP_103565569).

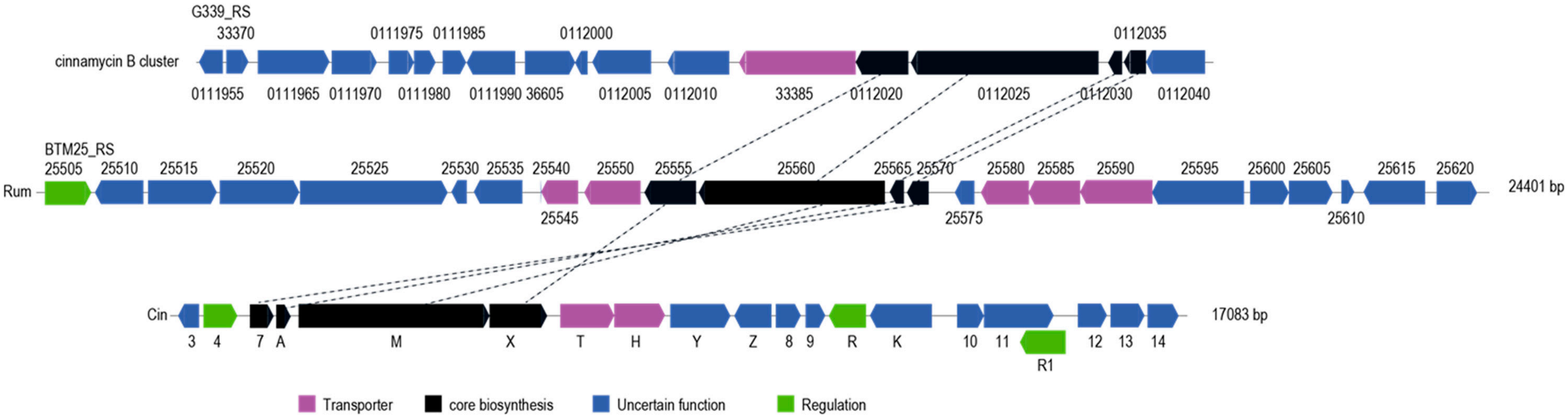

Figure S6. Comparative gene maps of *rum* and *cin* biosynthetic clusters and proposed cinnamycin B biosynthetic cluster..

Table S12. NMR Data (500 MHz, DMSO-d_6, at 300 K) for barceloneic acid A (**7**).

	barceloneic acid A (**7**)			
positio	δC, mult.[a]	δH, mult. (*J* in Hz)	COSY	HMBC
1	171.7, qC			
2	n.d.			
3	157.4, qC			
4	107.9, CH	5.95, br s		1, 3, 5, 6, 8
5	140.6, qC			
6	110.9, CH	6.26, br s		1, 3, 4, 7, 8
7	161.0, qC			
8	21.2, CH_3	2.07, s		4, 5, 6
9	136.2, qC			
10	151.6, qC			
11	101.1, CH	6.23, d (2.7)		9, 10, 12, 13
12	156.1, qC			
13	102.0, CH	6.44, d (2.7)		12, 14, 15
14	136.4, qC			
15	58.3, CH_2	4.51, br s		9, 10, 12, 13, 14
16	54.9, CH_3	3.68, s		12
		7.00, br s		

[a]:^{13}C chemical shifts were extracted from HSQC and HMBC spectra.

Table S13. NMR Data comparison with reported and isolated barceloneic acid A (**7**).

Position	barceloneic acid A[a]		barceloneic acid A[b]	
	δH, mult. (J in Hz)	δC, mult.	δH, mult. (J in Hz)	δC, mult.
1		171.86, qC		171.7, qC
2		100.91, qC		n.d.
3		159.14, qC		157.4, qC
4	6.03, br s	105.31, CH	5.95, br s	107.9, CH
5		147.85, qC		140.6, qC
6	6.50, br s	112.64, CH	6.26, br s	110.9, CH
7		164.40, qC		161.0, qC
8	2.18, s	22.00, CH_3	2.07, s	21,2, CH_3
9		132.36, qC		136.2, qC
10		151.03, qC		151.6, qC
11	6.54, d (3.0)	102.41, CH	6.23, d (2.7)	101.1, CH
12		159.25, qC		156.1, qC
13	6.68, d (3.0)	105.51, CH	6.44, d (2.7)	102.0, CH
14		137.38, qC		136.4, qC
15	4.53, br s	60.31, CH_2	4.51, br s	58.3, CH_2
16	3.80, s	55.74, CH_3	3.68, s	54.9, CH_3

[a] NMR data (acetone-d_6) from literature reported in ref. 4.
[b] NMR data (DMSO-d_6) of isolate

Table S14. NMR Data comparison with reported and isolated banegasine (**10**).

Position	banegasine[a]		banegasine[b]
	δ_H, mult. (J in Hz)	δ_C, mult.	δ_H, mult. (J in Hz)
1			
2	3.84, dd (9.3, 3.9)	56.8, CH	3.48, dd (8.6, 3.9)
3	3.13, dd (15.3, 9.6)	28.5, CH_2	2.99, dd (15.1, 8.9)
	3.50, dd (15.3, 3.9)		3.31, dd (15.1, 3.7)
4		109.7, qC	
5	7.18, s	125.1, CH	7.22, d (1.2)
6		174.5, qC	
1′		128.5, qC	
2′		138.4, qC	
3′	7.35, d (8.1)[c]	112.4, CH	7.35, d (8.1)
4′	7.11, td (8.4, 1.5)[c]	122.7, CH	7.06, t (7.8)
5′	7.03, td (7.8, 1.2)[c]	120.1, CH	6.97, t (7.2)
6′	7.68, d (8.1)[c]	119.3, CH	7.56, d (7.9)
			10.97, br s

[a] NMR data (MeOD-d_4) from literature reported in ref. 5.
[b] NMR data (600 MHz, DMSO-d_6, 300 K) of isolate.

Table S15. NMR Data (DMSO-d_6, at 300 K) for *cyclo*(*N*Me-L-3,5-dichlorotyrosine-Dhb) **(11).**[a]

position	*cyclo*(*N*Me-L-3,5-dichlorotyrosine-Dhb) (**11**)				
	δ_C, mult.[b]	δ_H, mult. (*J* in Hz)	COSY	HMBC	NOESY
1	165.5, qC				
2	62.5, CH	4.29, dd (5.0, 3.4)	3a, 3b	1, 3, 4, 8, 9	8
3	35.2, CH_2	3.03, dd (14.0, 5.0)	2	1, 2, 4, 5	2, 3b, 5
		2.92, dd (14.0, 3.4)	2	1, 2, 4, 5	2, 3a, 5
4	127.2, qC				
5	129.7, 2 CH	6.94, s, 2H		2, 3, 6, 7	3a, 3b, 8
6	121.9, 2 qC				
7	148.8, qC				
8	32.1, CH_3	2.94, s, 3H		2, 9	2, 5
9	159.0, qC				
10	126.9, qC				
11	112.0, CH	5.41, q, 7.5	12	9, 12	12
12	10.7, CH_3	1.46, d, 7.5	11	9, 10, 11	11, *N*H
*N*H		9.92, br s		1, 2, 9, 10	12

[a] 600 MHz for ^{1}H NMR and 150 MHz for ^{13}C NMR
[b] numbers of attached protons were determined by analysis of 2D spectra.

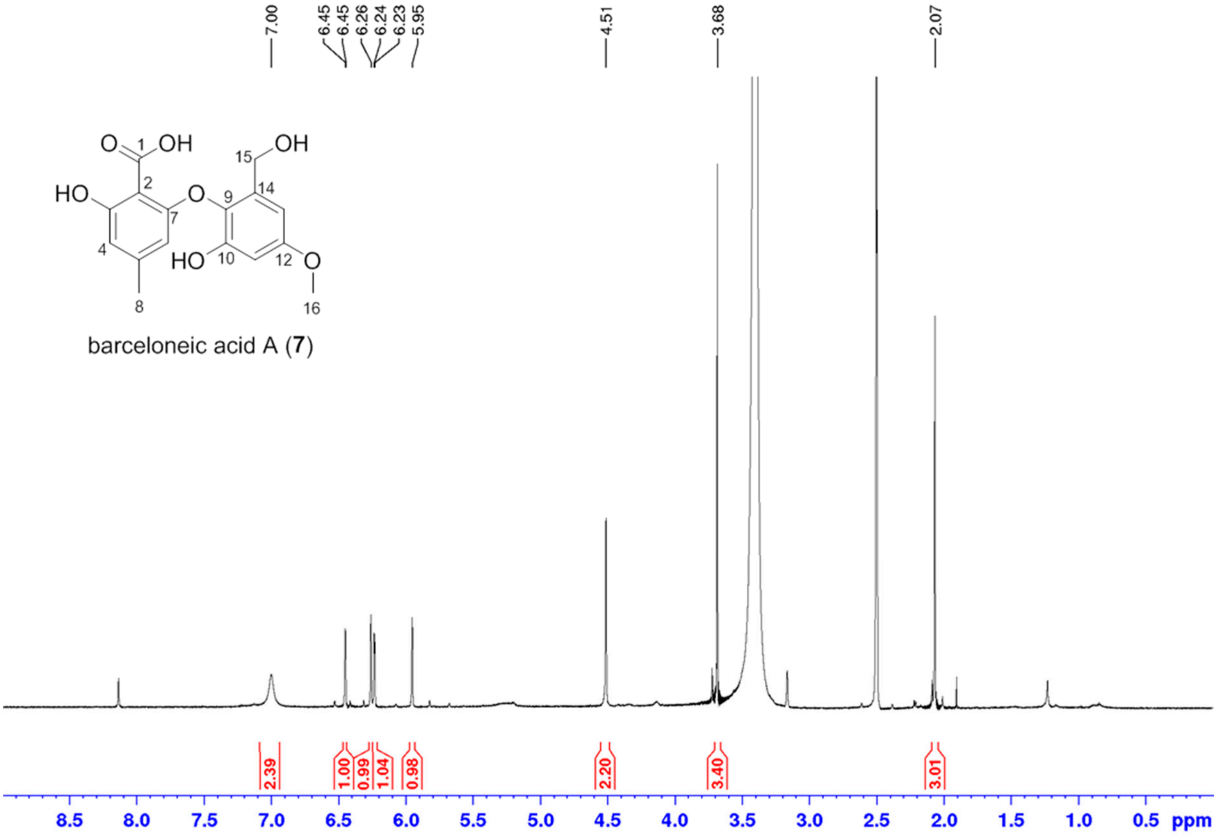

Figure S7. ^{1}H NMR spectrum of barceloneic acid A (**7**) (DMSO-d_6, 300K, 500 MHz).

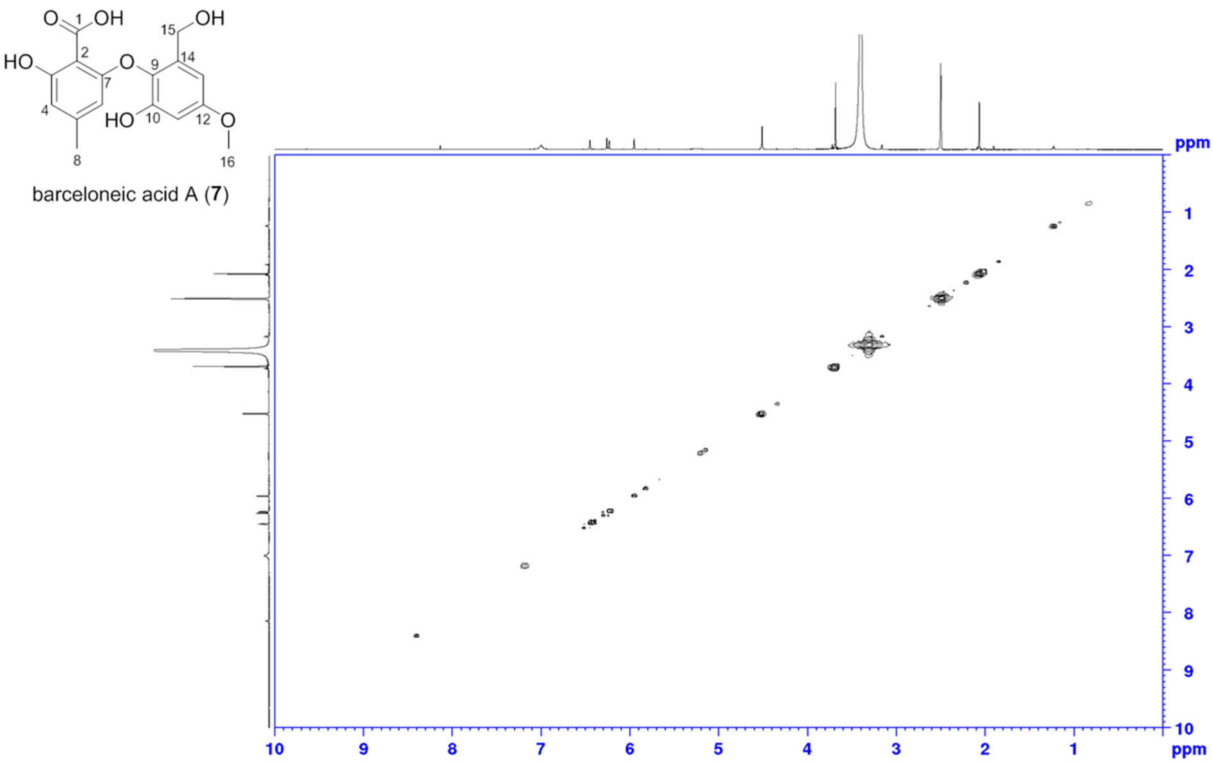

Figure S8. COSY spectrum of barceloneic acid A (**7**) (DMSO-d_6, 300K, 500 MHz).

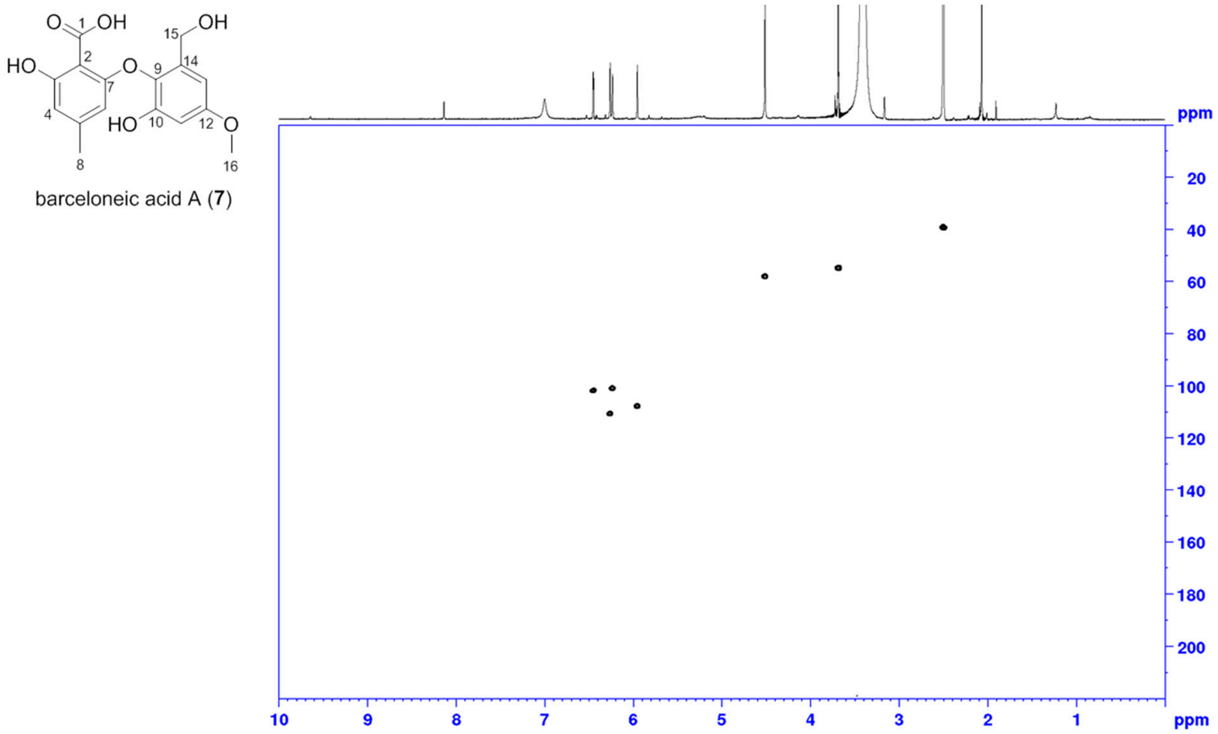

Figure S9. HSQC NMR spectrum of barceloneic acid A (**7**) (DMSO-d_6, 300K, 500 MHz).

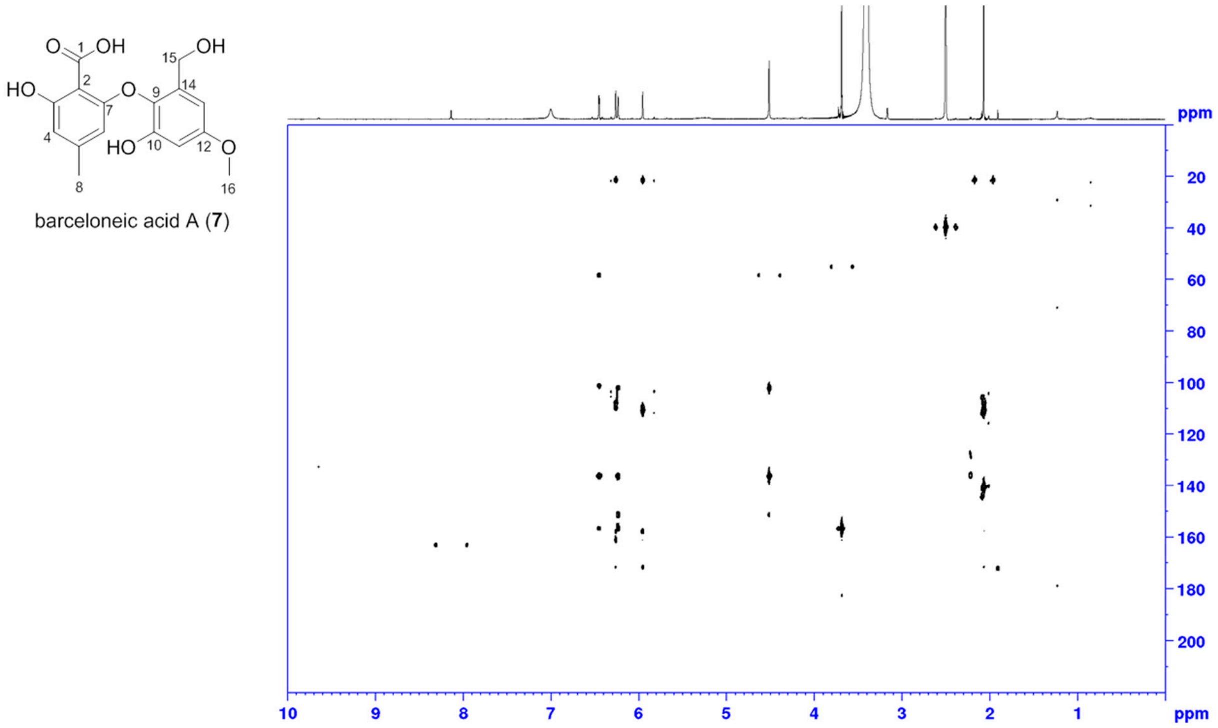

Figure S10. HMBC NMR spectrum of barceloneic acid A (**7**) (DMSO-d_6, 300K, 500 MHz).

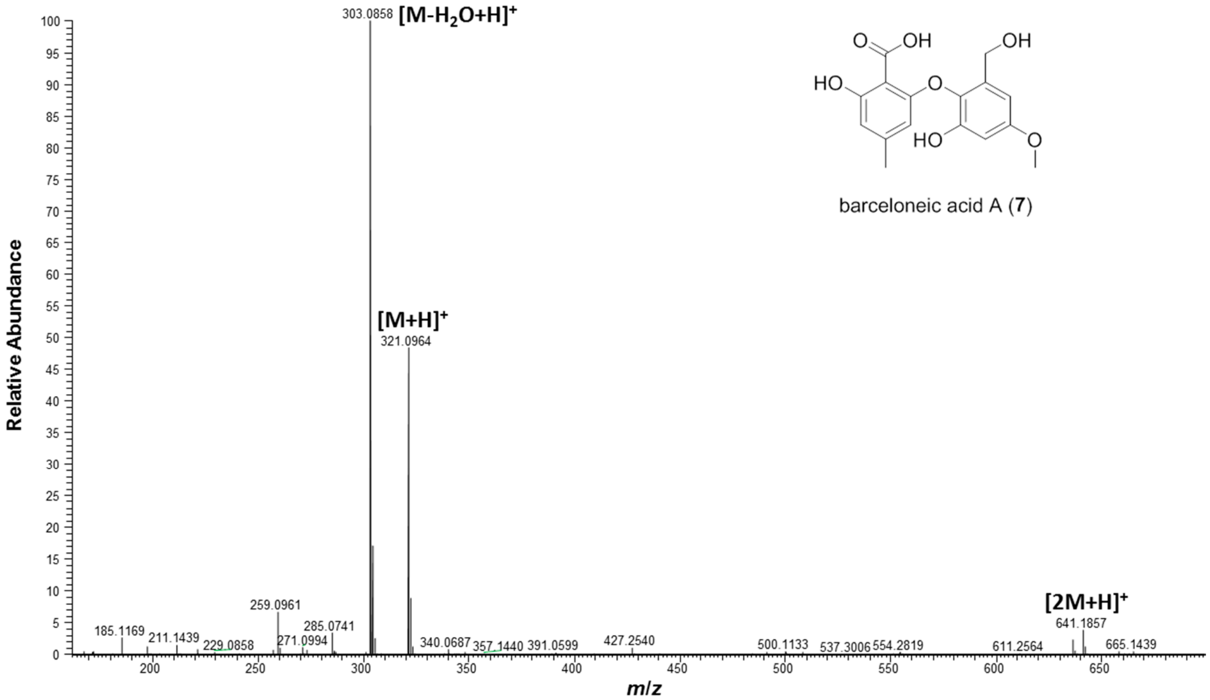

Figure S11. ESI-HRMS spectrum of barceloneic acid A (**7**) under the positive mode.

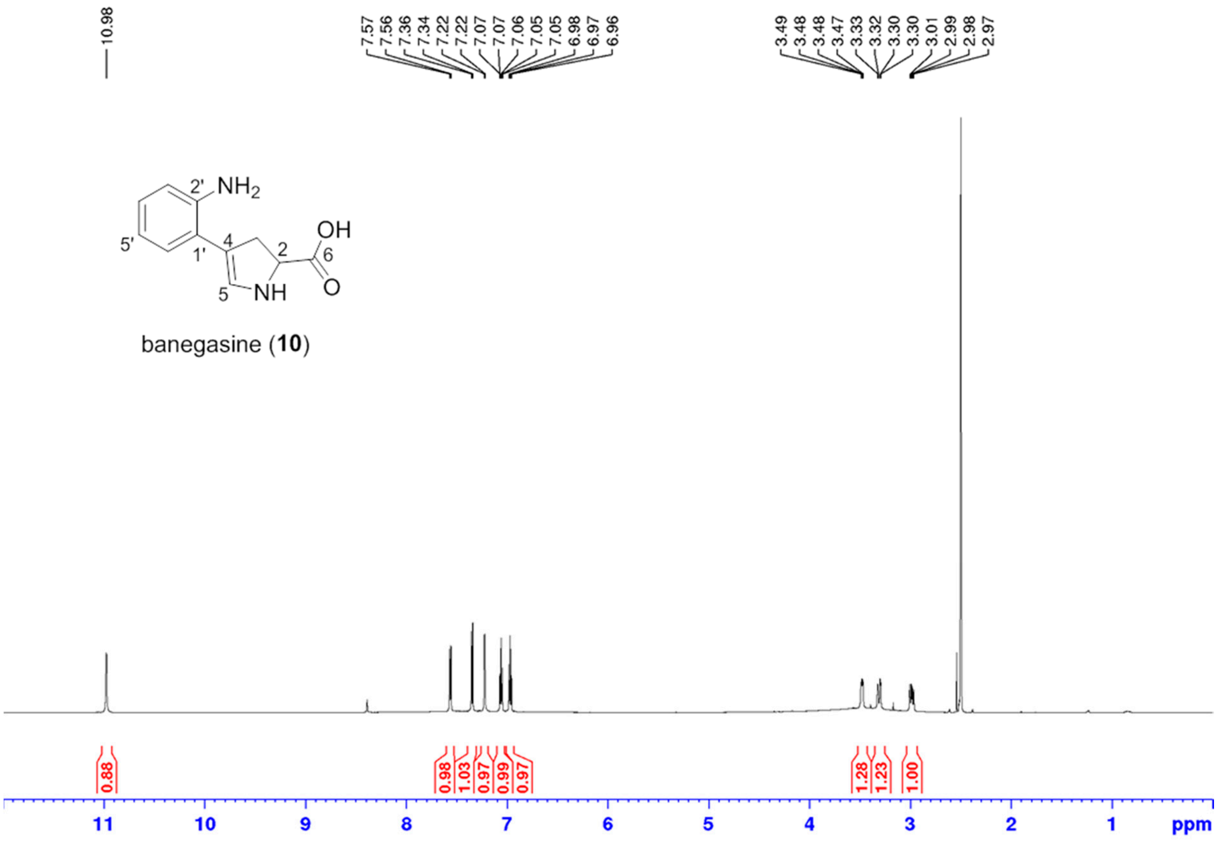

Figure S12. ^{1}H NMR spectrum of banegasine (**10**) (DMSO-*d*6, 300K, 600 MHz).

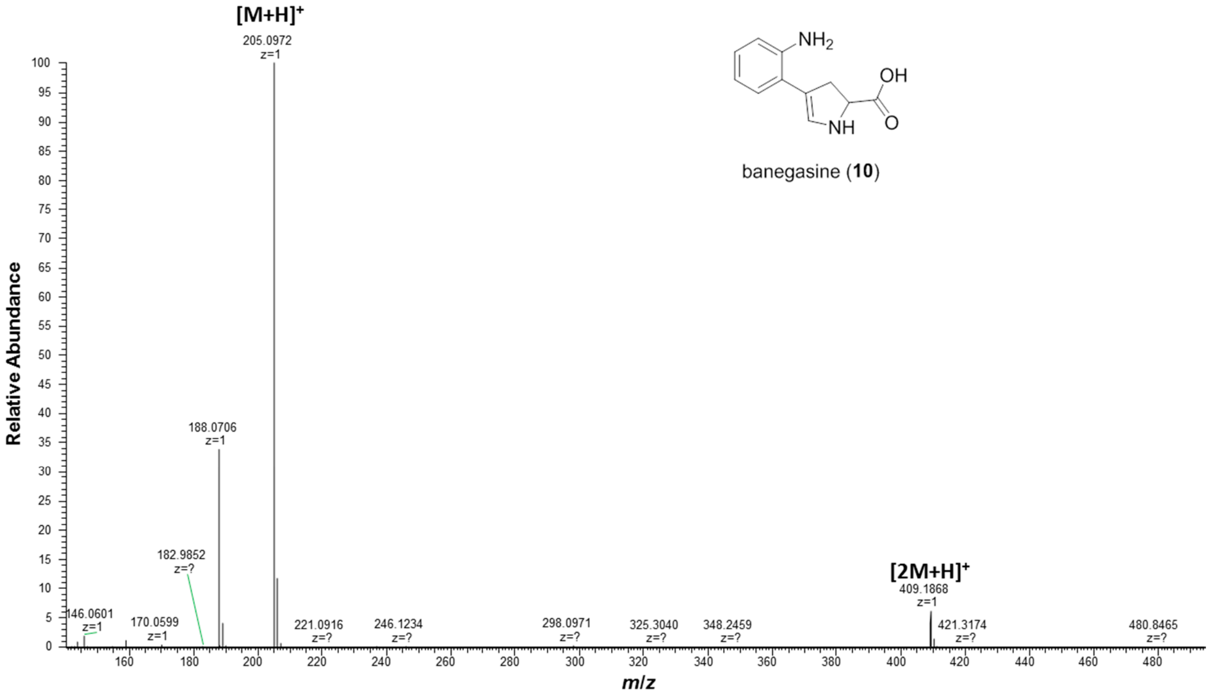

Figure S13. ESI-HRMS spectrum of banegasine (**10**) under the positive mode.

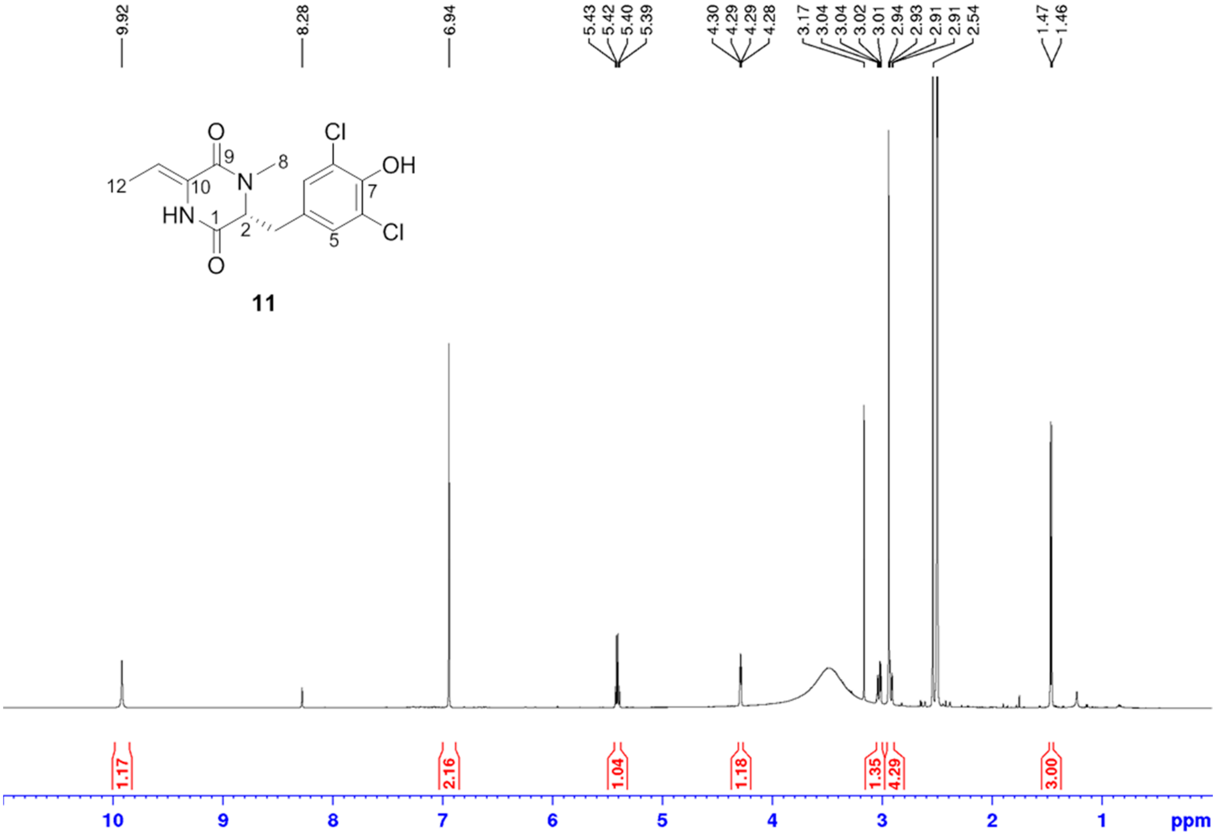

Figure S14. ^{1}H NMR spectrum of *cyclo*(*N*Me-L-3,5-dichlorotyrosin-Dhb) (**11**) (600 MHz, DMSO-d_6, 300 K).

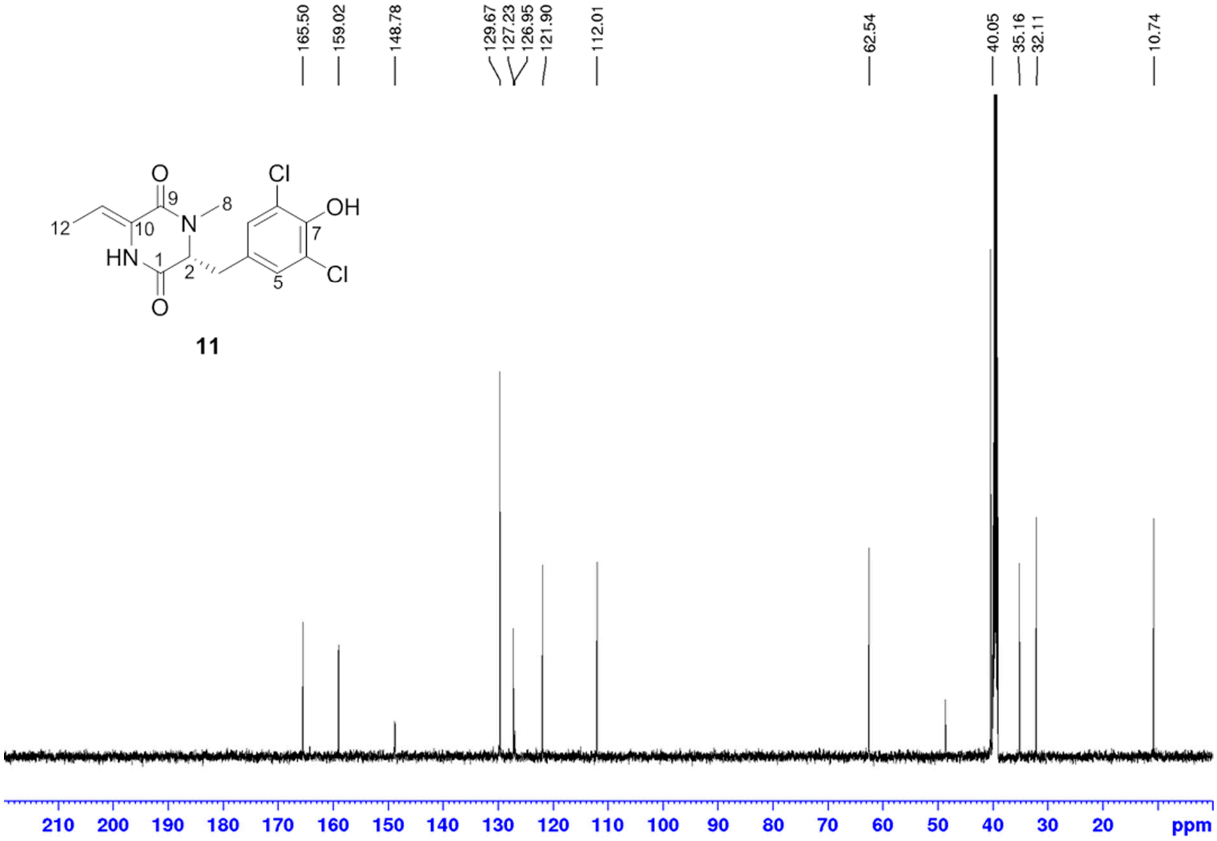

Figure S15. ^{13}C NMR spectrum of *cyclo*(*N*Me-L-3,5-dichlorotyrosin-Dhb) (**11**) (150 MHz, DMSO-d_6, 300 K).

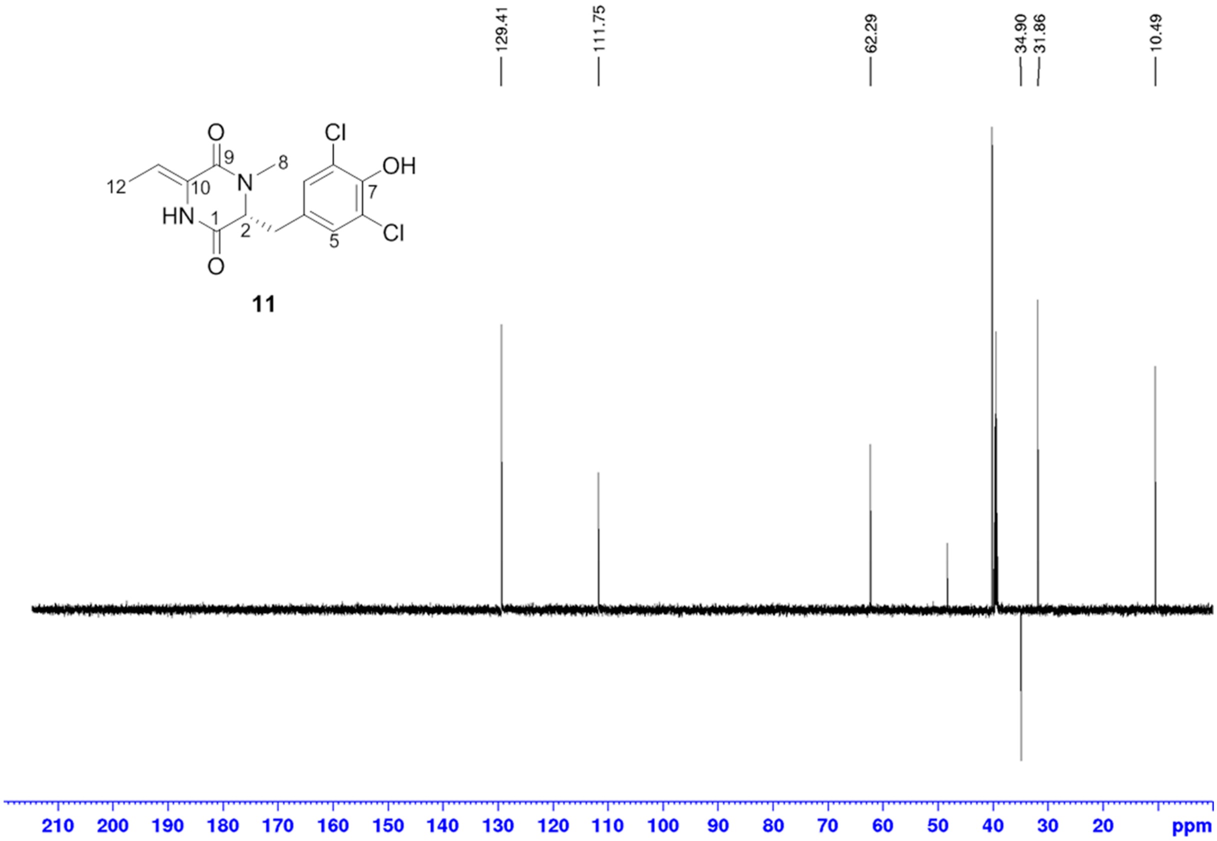

Figure S16. DEPT135 spectrum of *cyclo*(*N*Me-L-3,5-dichlorotyrosin-Dhb) (**11**) (600 MHz, DMSO-d_6, 300 K).

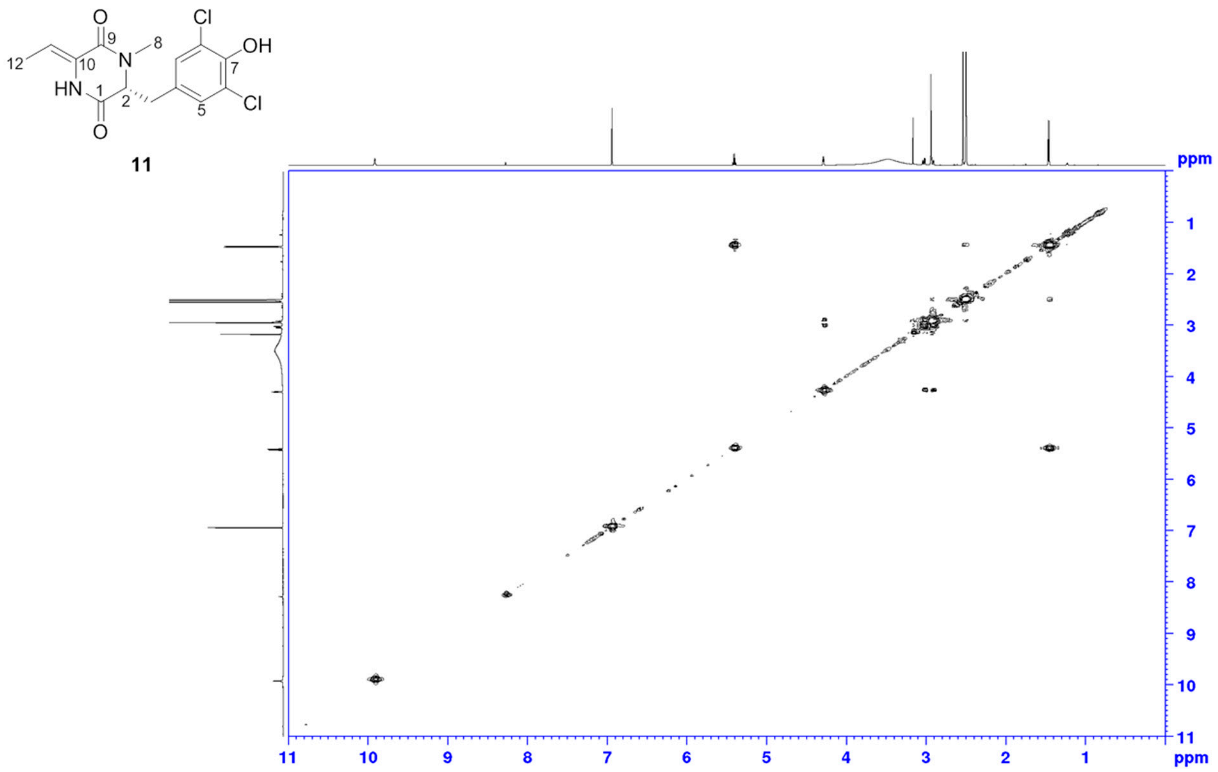

Figure S17. COSY spectrum of *cyclo*(*N*Me-L-3,5-dichlorotyrosin-Dhb) (**11**) (600 MHz, DMSO-d_6, 300 K).

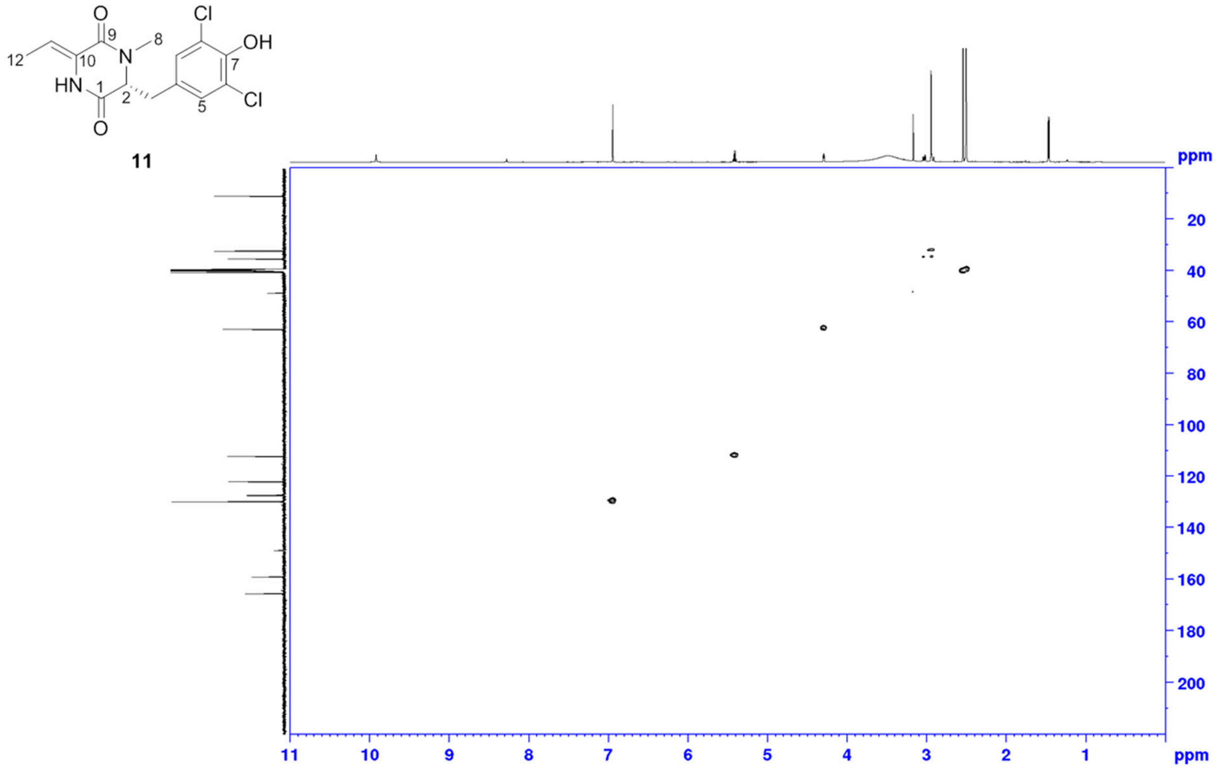

Figure S18. HSQC spectrum of *cyclo*(*N*Me-L-3,5-dichlorotyrosin-Dhb) (**11**) (600 MHz, DMSO-d_6, 300 K).

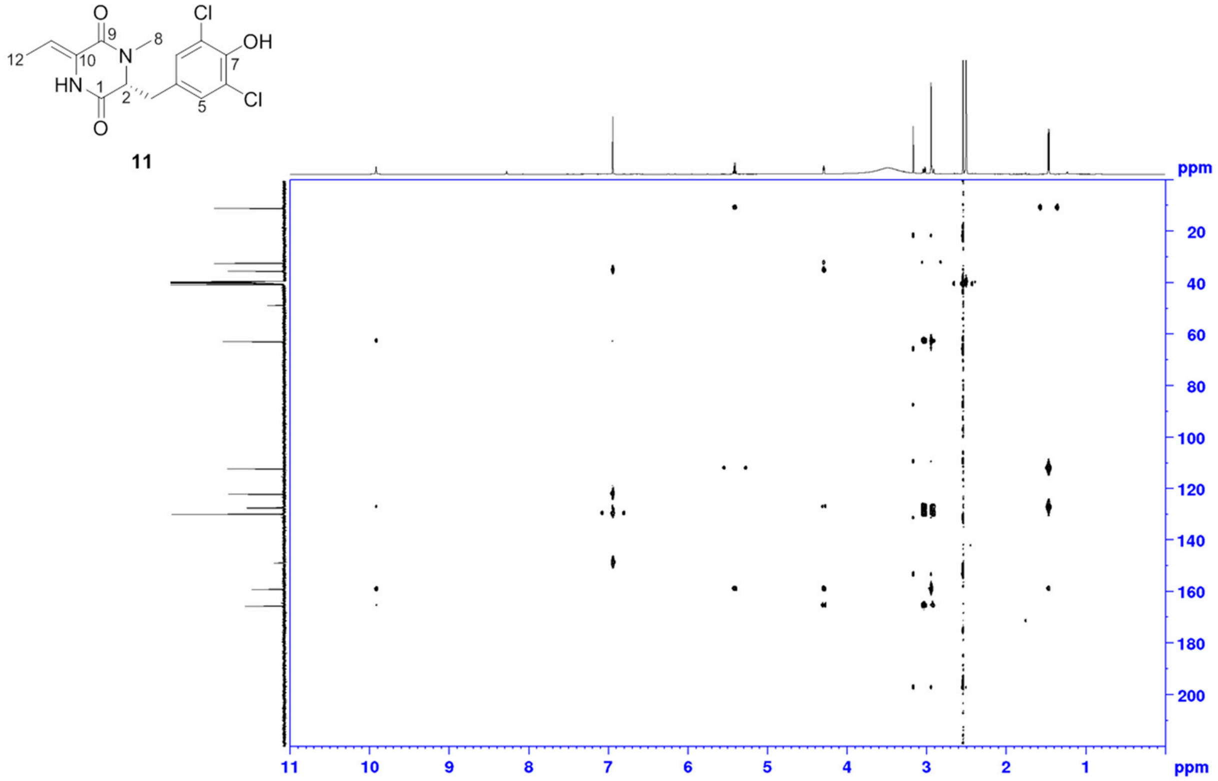

Figure S19. HMBC spectrum of *cyclo*(*N*Me-L-3,5-dichlorotyrosin-Dhb) (**11**) (600 MHz, DMSO-d_6, 300 K).

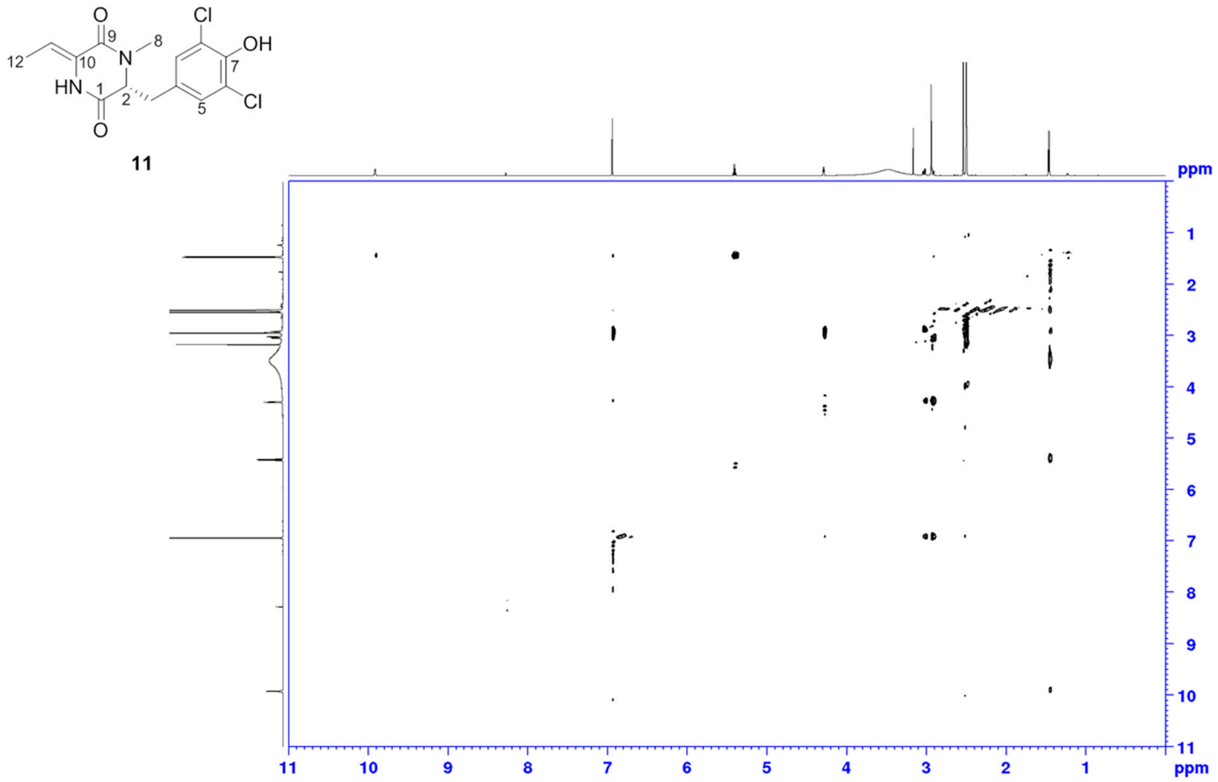

Figure S20. NOESY spectrum of *cyclo*(*N*Me-L-3,5-dichlorotyrosin-Dhb) (**11**) (600 MHz, DMSO-d_6, 300 K).

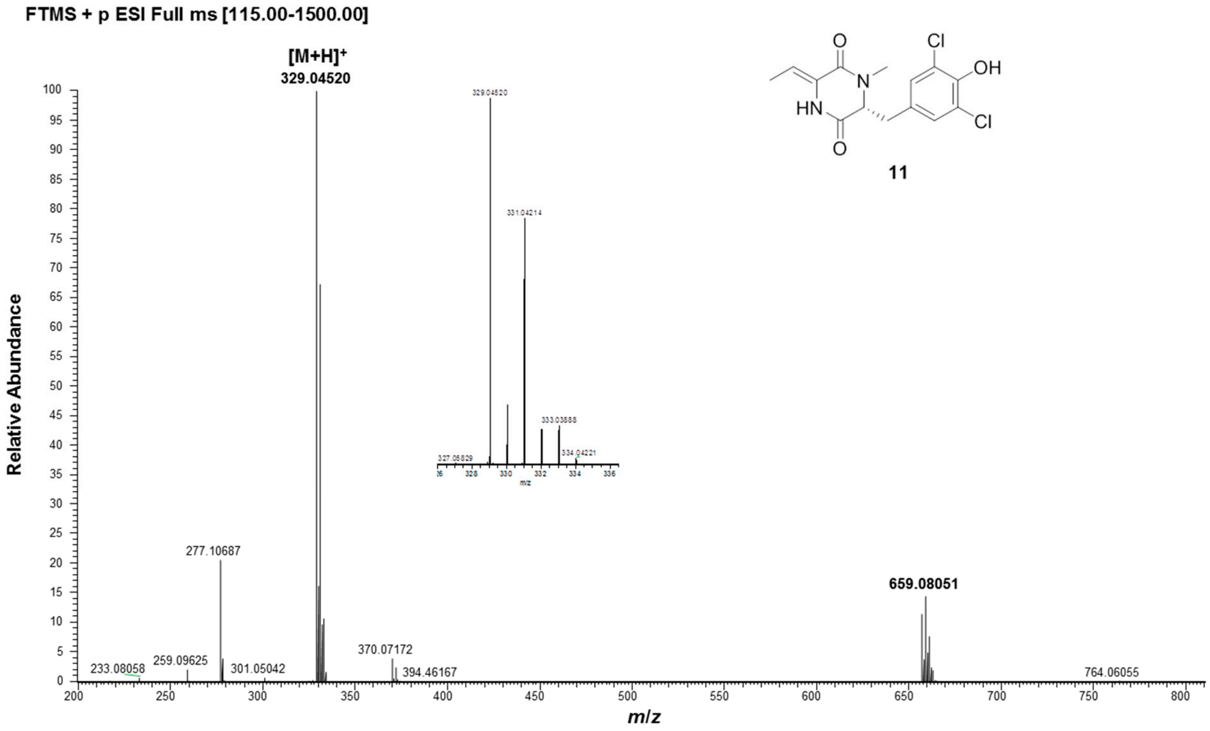

Figure S21. ESI-HRMS spectrum of *cyclo*(*N*Me-L-3,5-dichlorotyrosin-Dhb) (**11**).

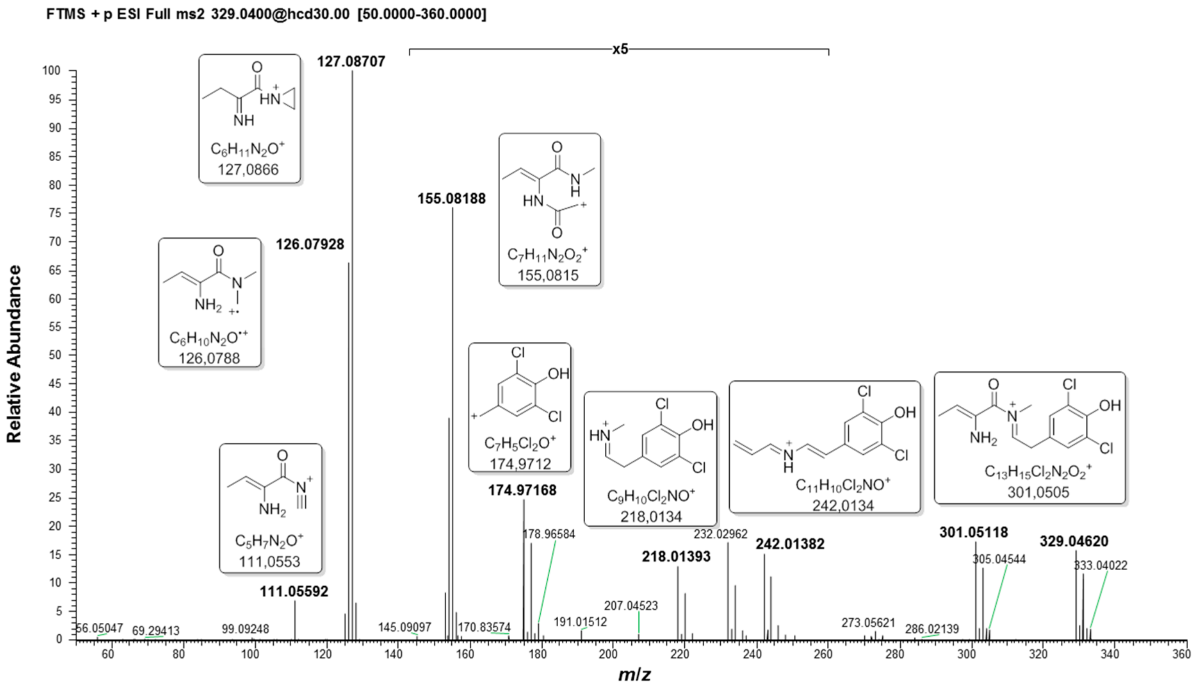

Figure S22. ESI-HRMS^2 spectrum of *cyclo*(*N*Me-L-3,5-dichlorotyrosin-Dhb) (**11**).

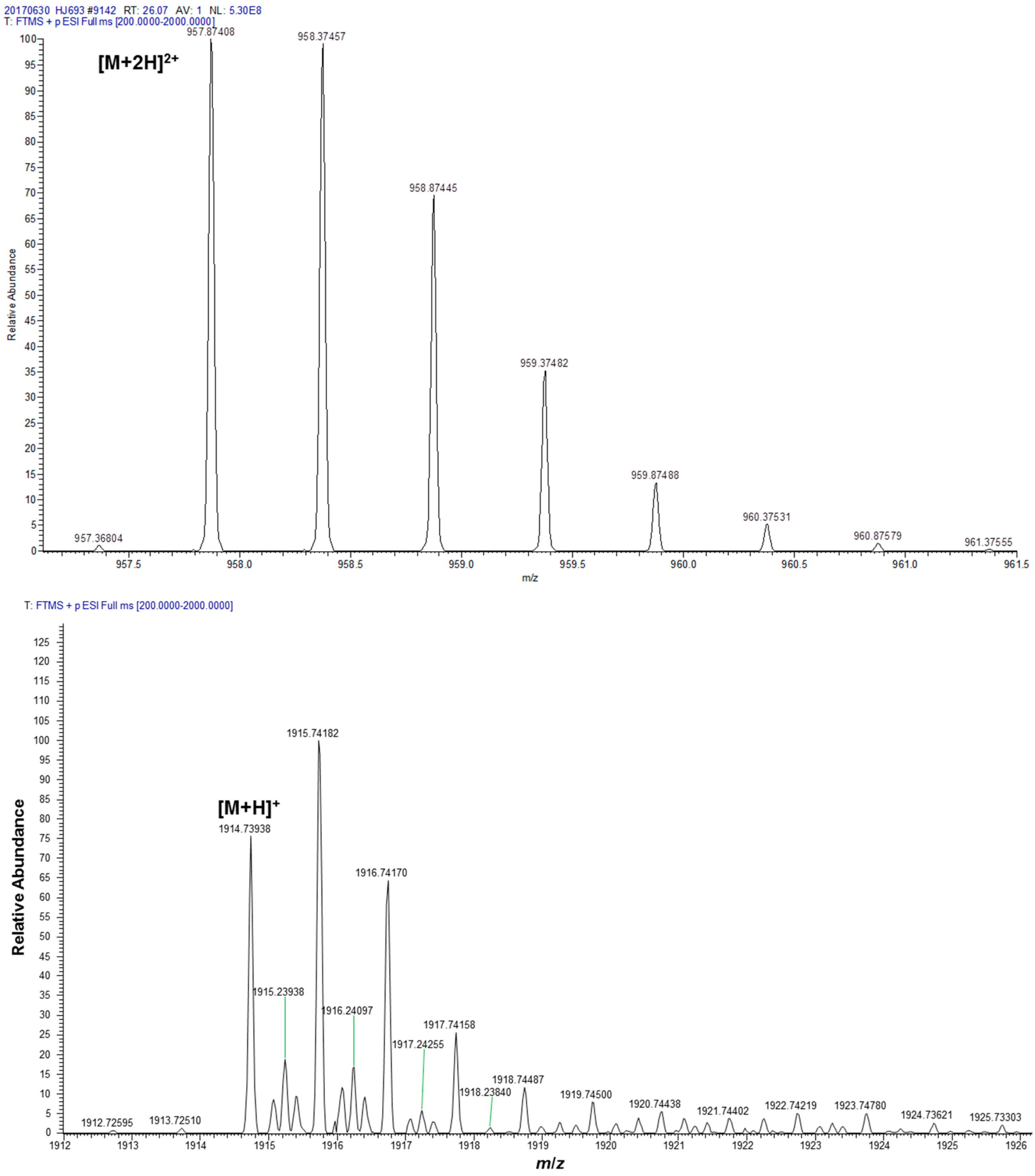

Figure S23. ESI-HRMS spectrum of rubrominin A (**12**).

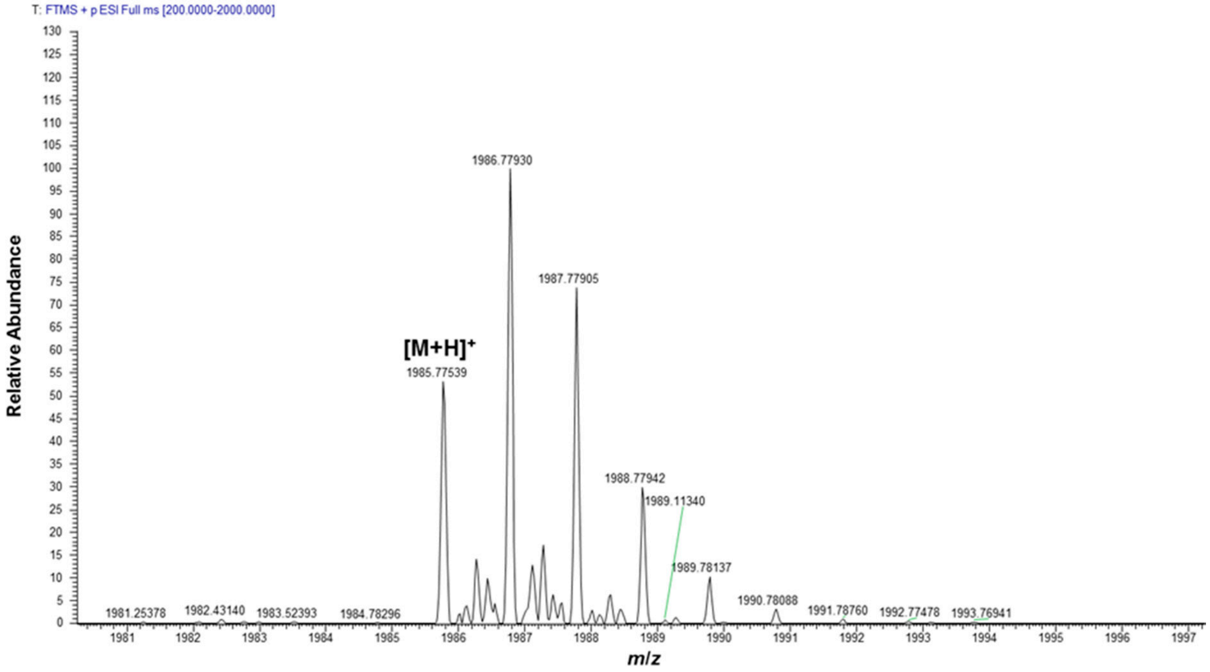

Figure S24. ESI-HRMS spectrum of rubrominin B (**13**).

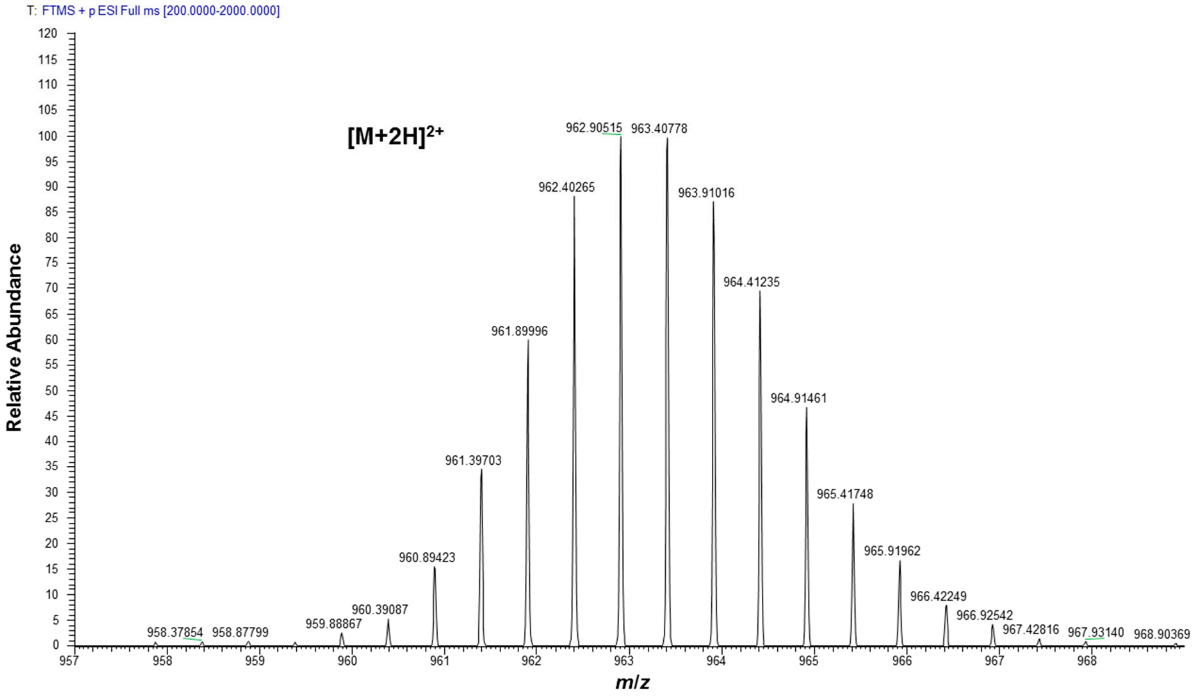

Figure S25. ESI-HRMS spectrum of rubrominin A (**12**) labled with L-Serine-2,3,3-d3.

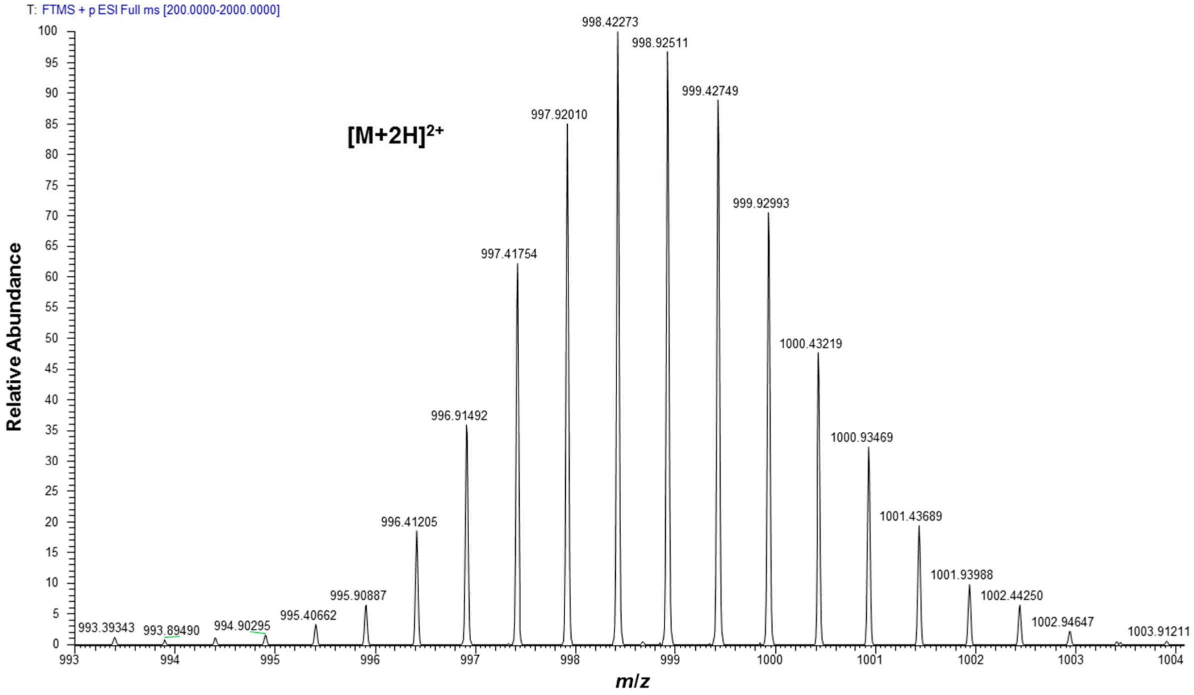

Figure S26. ESI-HRMS spectrum of rubrominin B (**13**) labled with L-serine-2,3,3-D3.

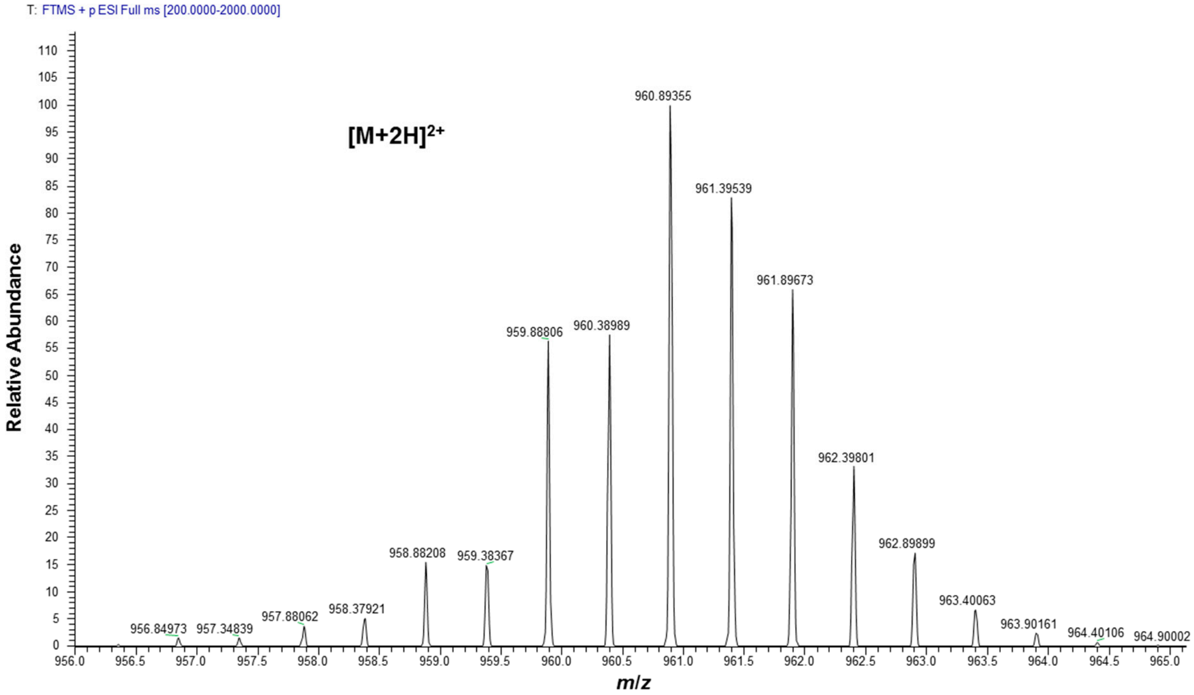

Figure S27. ESI-HRMS spectrum of rubrominin A (**12**) labled with DL-cystein-3,3-D2.

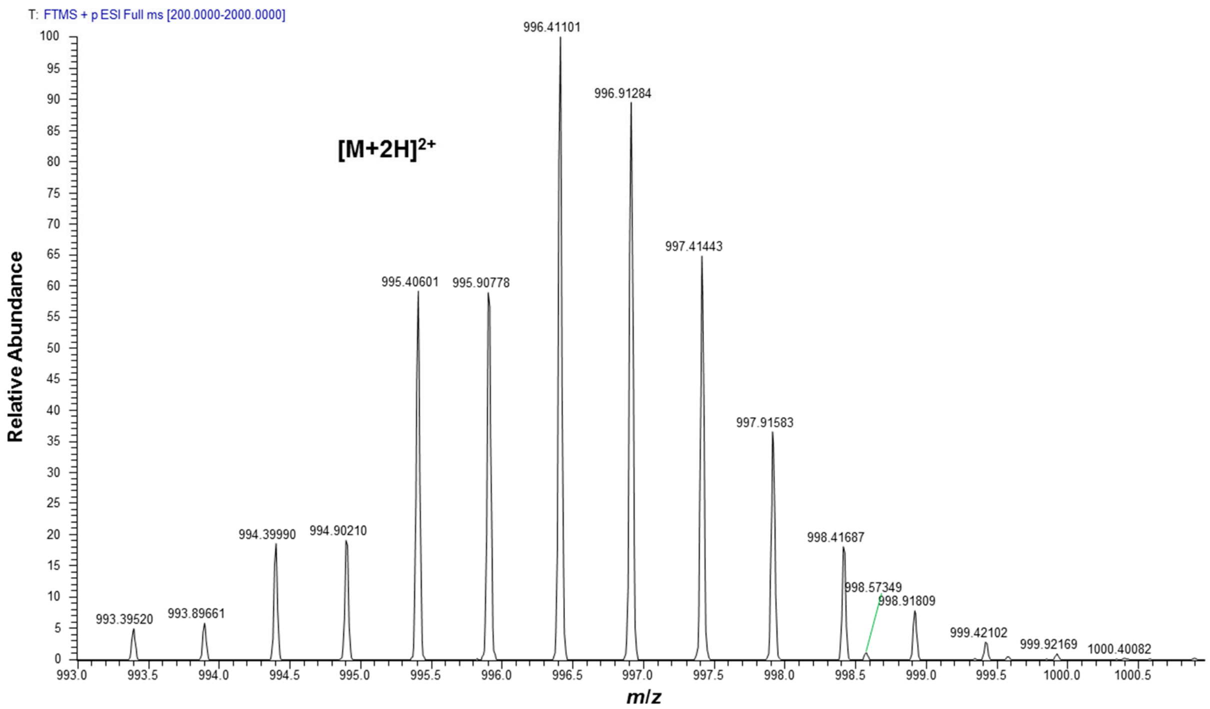

Figure S28. ESI-HRMS spectrum of rubrominin B (**13**) labled with DL-cystein-3,3-D2.

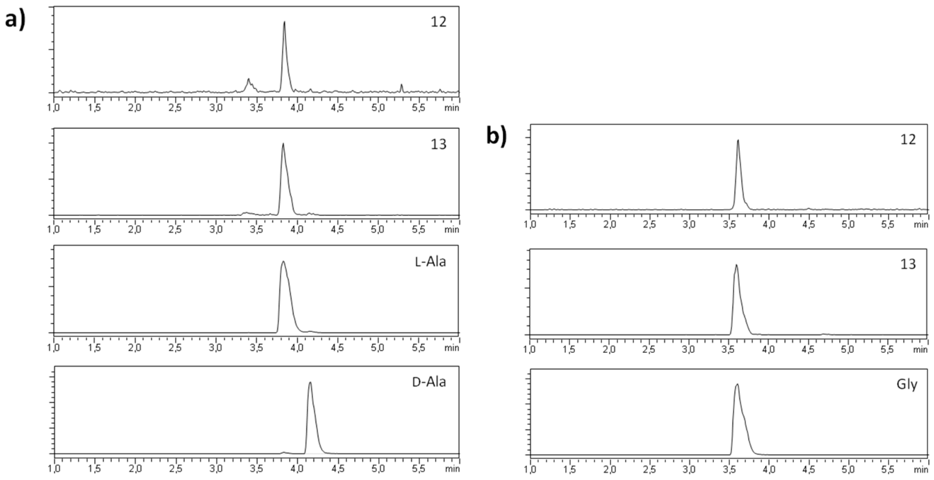

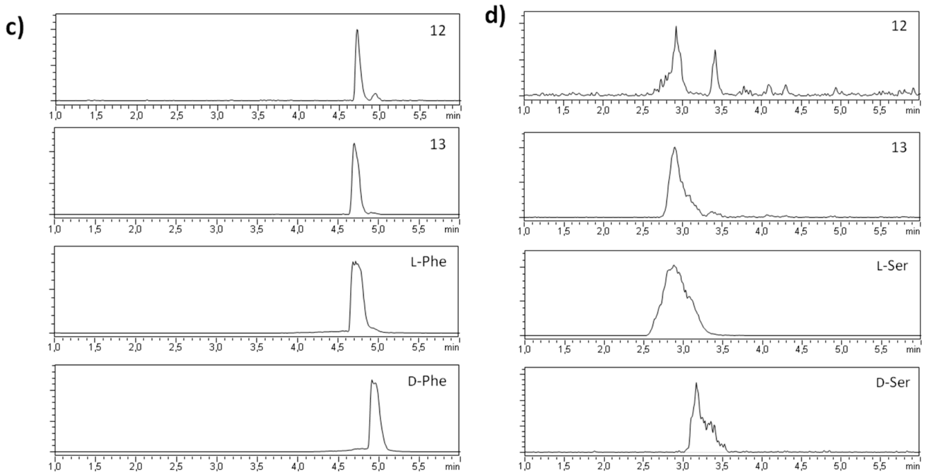

Figure S29. UHPLC-MS chromatogram of Marfey’s reaction (1): a) extracted ion chromatograms of Marfey’s derivative of standard L-alanine (m/z 342.00, tR = 3.83 min), D-alanine (m/z 342.00, tR = 4.17 min); b) extracted ion chromatograms of Marfey’s derivative of standard glycine (m/z 328.00, tR = 3.60 min); c) extracted ion chromatograms of Marfey’s derivative of standard L-phenylalanine (m/z 418.00, tR = 4.73 min), D-phenylalanine (m/z 418.00, tR = 4.93 min); d) extracted ion chromatograms of Marfey’s derivative of standard L-serine (m/z 358.00, tR = 2.94 min), D-serine (m/z 358.00, tR = 3.15 min).

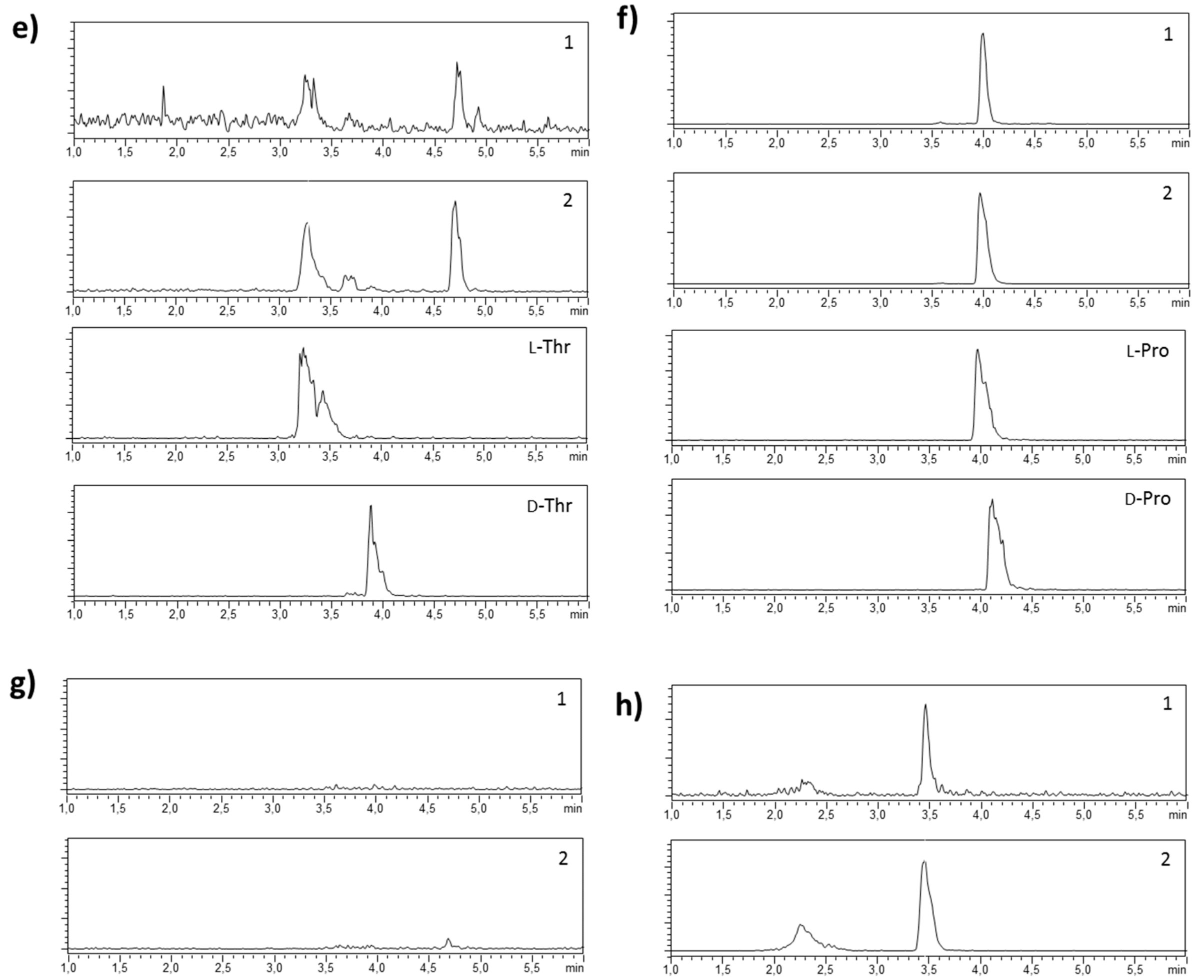

Figure S30. UHPLC-MS chromatogram of Marfey's reaction (2): e) extracted ion chromatograms of Marfey's derivative of standard L-theorine (m/z 372.00, tR = 3.26 min), D-theorine (m/z 372.00, tR = 3.87 min); f) extracted ion chromatograms of Marfey's derivative of standard L-proline (m/z 368.00, tR = 3.98 min), D-proline (m/z 368.00, tR = 4.12 min); g) extracted ion chromatograms of proposed Marfey's derivative of lanthionine (m/z 461.0); h) extracted ion chromatograms of proposed Marfey's derivative of methyllanthionine (m/z 475.0) .

REFERENCES

[1] Meier-Kolthoff, J.P.; Goker, M.; Sproer, C.; Klenk, H.P. When should a DDH experiment be mandatory in microbial taxonomy? *Arch. Microbiol.* **2013**, *195*, 413–418.

[2] Eddy, S.R. A probabilistic model of local sequence alignment that simplifies statistical significance estimation. *PLoS Computational Biology* **2008**, *4*, e1000069

[3] Kodani, S.; Komaki, H.; Ishimura, S.; Hemmi, H.; Ohnishi-Kameyama, M. Isolation and structure determination of a new lantibiotic cinnamycin b from *Actinomadura atramentaria* based on genome mining. *J. Ind. Microbiol. Biotechnol*. **2016**, *43*, 1159-1165.

[4] Jayasuriya, H.; Ball, R.G.; Zink, D.L.; Smith, J.L.; Goetz, M.A.; Jenkins, R.G.; Nallin-Omstead, M.; Silverman, K.C.; Bills, G.F.; Lingham, R.B.; Singh, S. B.; Pelaez, F.; Cascales, C. *J. Nat. Prod.* **1995**, *58*, 986–991.

[5] Cain, C.C.; Lee, D.; Waldo Iii, R.H.; Henry, A.T.; Casida, E.J.; Wani, M.C.; Wall, M.E.; Oberlies, N.H.; Falkinham Iii, J.O. Synergistic antimicrobial activity of metabolites produced by a nonobligate bacterial predator. *Antimicrob. Agents. Chemother.* **2003**, *47*, 2113–2117.
